# (Meth)acrylate-Free Three-Dimensional Printing of Bio-Derived Photocurable Resins with Terpene- and Itaconic Acid-Derived Poly(ester-thioether)s

**DOI:** 10.1021/acssuschemeng.3c04576

**Published:** 2023-11-28

**Authors:** Mirko Maturi, Chiara Spanu, Emanuele Maccaferri, Erica Locatelli, Tiziana Benelli, Laura Mazzocchetti, Letizia Sambri, Loris Giorgini, Mauro Comes Franchini

**Affiliations:** † Department of Industrial Chemistry “Toso Montanari”, University of Bologna, Viale Risorgimento 4, Bologna 40136, Italy; ‡ Interdepartmental Center for Industrial Research on Advanced Applications in Mechanical Engineering and Materials Technology, CIRI-MAM, University of Bologna, Viale Risorgimento 2, Bologna 40136, Italy

**Keywords:** poly(ester-thioether), itaconic acid, vat photopolymerization, biobased materials, photopolymerization

## Abstract

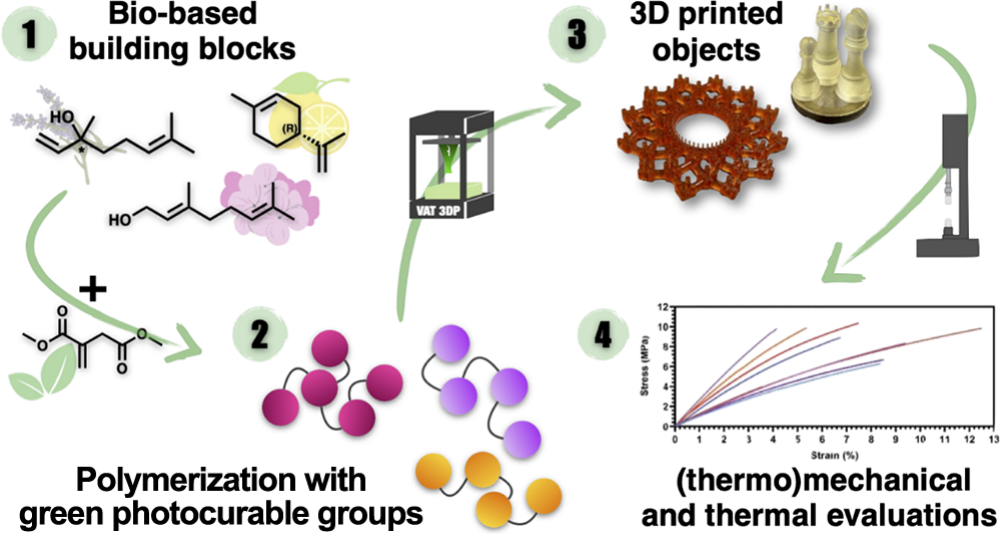

Vat photopolymerization, a very efficient and precise object manufacturing technique, still strongly relies on the use of acrylate- and methacrylate-based formulations because of their low cost and high reactivity. However, the environmental impact of using fossil fuel-based, volatile, and toxic (meth)acrylic acid derivatives is driving the scientific community toward the development of alternatives that can match the mechanical performance and three-dimensional (3D) printing processability of traditional photocurable mixtures but are made from environmentally friendly building blocks. Herein, itaconic acid is polymerized with polyols derived from naturally occurring terpenes to produce photocurable poly(ester-thioether)s. The formulation of such polymers using itaconic acid-based reactive diluents allows the preparation of a series of (meth)acrylate-free photocurable resins, which can be 3D printed into solid objects. Extensive analysis has been conducted on the properties of photocured polymers including their thermal, thermomechanical, and mechanical characteristics. The findings suggest that these materials exhibit properties comparable to those of traditional alternatives that are created using harmful and toxic blends. Notably, the photocured polymers are composed of biobased constituents ranging from 75 to 90 wt %, which is among the highest values ever recorded for vat photopolymerization applications.

## Abstract 2

Terpene-based polyols are polymerized with itaconic acid to achieve photocurable poly(ester-thioether)s and then formulated in biobased resins for stereolithographic 3D printing.

## Introduction

Three-dimensional (3D) printing refers to a set of computer-aided additive manufacturing techniques that allow the bottom-up assembly of 3D objects via a layer-by-layer approach using polymeric, metallic, or ceramic raw materials. The starting material is assembled by exploiting various physical or chemical transformations that depend on the manufacturing technology to construct solid 3D objects with higher versatility and complexity and minimum material wastage compared to traditional subtractive manufacturing techniques such as computer numerical control (CNC) machining, injection molding, plastic forming, and plastic joining.^1−3^ The entire additive manufacturing industry demands lower energetic and financial resources compared to traditional techniques, reducing the cost of the final object and overall CO_2_ emissions during object manufacturing. In fact, Gebler at al. assessed that from 2015 to 2025, 3D printing will reduce the manufacturing costs by USD 170–593 billion, total primary energy supply by 2.54–9.30 EJ, and CO_2_ emissions by 130.5–525.5 Mt.^4^ Among the different 3D printing technologies, vat photopolymerization has attracted attention from the scientific community and industrial manufacturing sector because it can be used to assemble numerous polymeric materials into complex shapes with very high resolutions, which have applications ranging from biomedicine to dentistry and jewelry.^5−8^ Vat photopolymerization is the spatially confined photopolymerization of a liquid resin placed in a transparent vat using a light source (usually ultraviolet (UV) or blue visible radiation). Vat photopolymerization is usually used for manufacturing polymeric thermoset or elastomeric materials and relies on the radical photopolymerization of acrylates,^9,10^ methacrylates,^11,12^ or thiol–ene^13,14^ multicomponent systems. Although (meth)acrylates photopolymerize with high efficiency to form 3D objects with tunable mechanical properties, their abundant use is attracting increasing concern because of their toxicity and environmental impact. For example, the main synthesis process of (meth)acrylates is the acetone cyanohydrin (ACH) route, where acetone and hydrocyanic acid are used as raw materials.^15^

In fact, most commercially available formulations for vat photopolymerization are fabricated using nonrenewable petrochemical resources. Consequently, part of the scientific community is directing its efforts toward replacing (meth)acrylates with photocurable compounds that can be derived from natural and renewable resources.^16^ However, in most studies, biomass-derived chemicals are exploited after functionalization it with meth(acrylic) acid, limiting the overall biobased content of photocurable formulations.^17−20^ Moreover, because nonrenewable source-derived polythiolated molecules such as pentaerythritol tetrakis(3-mercaptopropionate) are usually used in thiol–ene photopolymerization systems, the simple replacement of (meth)acrylate with free thiols or terminal alkenes does not address sustainability issues.^21^ To the best of our knowledge, to date, only one study has reported the use of (meth)acrylate- and thiol-free resins for vat photopolymerization.^22^ In this work, Pérocheau Arnaud et al. described the novel synthesis of itaconic acid esters as reactive diluents for vat photopolymerization, which were formulated using the appropriate photoinitiating system and poly(propylene itaconate-*co*-propylene sebacate) and 3D printed into materials with biobased contents as high as 72% and storage moduli of <200 MPa.

Itaconic acid or 2-methylenesuccinic acid is a photocurable dicarboxylic acid that was historically obtained via the distillation of citric acid; it is currently produced via the fermentation of biomasses.^23,24^ Like (meth)acrylates, itaconic acid can be reacted with alcohols to form liquid homodiesters or heterodiesters that can be efficiently 3D printed.^22^ However, unlike (meth)acrylates, itaconic acid has two carboxylic acid units that allow its prepolymerization into photocurable polyesters and poly(ester–amide)s with biobased diols and amidodiols, respectively, which are, as reported in our previous studies, major components of biobased photocurable resins for vat photopolymerization.^25,26^ Moreover, itaconic acid was recently included in the list of the top 12 building block chemicals by the US Department of Energy because of its sustainable production process, negligible toxicity, and extensive applicability.^27,28^

Terpenes are a wide class of unsaturated naturally abundant organic compounds composed of at least two isoprene units that are connected into linear (e.g., myrcene) or cyclic structures (e.g., limonene, pinene, and terpinene) and, in some cases, eventually oxidized (e.g., menthol, linalool, geraniol, and carvone).^29^ Their versatility has prompted their recent applications in photopolymerization-based 3D printing technologies.^30^ The presence of reactive C=C unsaturations and oxidized functional groups on the chemical structure of terpenes allows their chemical modification for the synthesis of biobased polymers. For example, Firdaus and co-workers reported the synthesis of diol and diester monomers via the thiol–ene addition of 2-mercaptoethanol or methyl 2-mercaptopropionate to the unsaturations of limonene and pinene, forming difunctional thioether monomers that can be used for the synthesis of poly(ester-thioether)s.^31^

This study reports the synthesis of three thioether polyols via the thiol–ene addition of 2-mercaptoethanol to three terpenes (i.e., limonene, linalool, and geraniol) and their polymerization with the dimethyl ester of itaconic acid to afford a set of photocurable poly(ester-thioether)s. After thorough characterization of synthesized monomers and polymers, photocurable poly(ester-thioether)s are formulated with a low-molecular-weight difunctional itaconic acid-derived cross-linker and an opportune photoinitiating system to afford biobased photocurable liquid resins that can be used for vat photopolymerization. In addition, this study quantitatively evaluates the maximum theoretical biobased content of each formulation according to the specifications of the OK BIOBASED labeling for plastics, assigned by TUV Austria.^32,33^ This approach is a modern and sustainable route to developing formulations for 3D printing. The thermal, thermomechanical, and mechanical properties of 3D-printed materials have been comprehensively characterized.

## Experimental Section

All chemicals were purchased from Sigma-Aldrich Co. and used as received. Grindsted Soft-N-Safe (9-hydroxystearic acid monoglyceride triacetate, SNS) was purchased from Danisco (Brabrand, Denmark). Hygroscopic 1,4-butanediol (BDO) was dried with sodium metal and distilled under a high vacuum before use.

### Synthesis of Thioether-Polyol Monomers **1**, **2**, and **3** from Terpenes

Liquid terpene (limonene for compound **1**, linalool for compound **2**, or geraniol for compound **3**, 0.5 mol) was placed in a 250 mL flat-bottomed flask equipped with a magnetic stirrer, followed by the addition of 97.7 g of 2-mercaptoethanol (1.25 mol, 87.7 mL). The mixture was cooled to 0 °C, and phenyl bis(2,4,6-trimethylbenzoyl) phosphine oxide (BAPO) was added as the radical photogenerator (1 mmol for **1** and **2**, 10 mmol for **3**). The addition of thiols to the unsaturations of the terpenes was performed by placing the cold and closed flask in a photoreactor equipped with blue LED light (400–450 nm, 18 W) until complete conversion was achieved, detected by testing the reaction mixture with NMR spectroscopy. The complete conversion was achieved after 6 h for **1**, 48 h for **2**, and 120 h for **3**. Purification of the viscous liquid product from the excess thiol was performed by vigorously stirring the reaction mixture with aqueous NaOH (1 M) until a stable alkaline pH was achieved. The product was recovered from the emulsion using ethyl acetate (3 × 200 mL), which was then first dried with brine (1 × 100 mL) and subsequently over Na_2_SO_4_. The liquid thioether-polyols were recovered as colorless liquids by rotary evaporation of the organic solvent and subjected to NMR, ESI-MS, and ATR-FTIR spectroscopies to assess the outcome of the reaction and the purity of the product. In particular, for product **1**, mass spectrometry (Figure S1) revealed the peak for the molecular ion as the most intense signal ([M + Na]^+^ = 315) and a few fragmentation peaks. ATR-FTIR spectra are reported (Figure S2), where marked peaks have been assigned to O–H stretching (3355 cm^–1^), C–H stretching (2920 cm^–1^), CH_2_ scissoring (1453 cm^–1^), O–H bending (1378 cm^–1^), and C–O stretching (1050 and 1008 cm^–1^). A low intensity band in the 650–700 cm^–1^ region can also be observed, and it has been attributed to the C–S–C thioether stretching mode. Similarly, for product **2**, mass spectrometry (Figure S3) and ATR-FTIR spectroscopy (Figure S4) revealed, respectively, the molecular ion at *m*/*z* = 333 (M+Na^+^) and infrared absorptions at 3355 cm^–1^ (O–H stretching), 2960 cm^–1^ (C–H stretching), 1462 cm^–1^ (CH_2_ scissoring), 1376 cm^–1^ (O–H bending), and 1043 and 1011 cm^–1^ (C–O stretching). Finally, for product **3**, the molecular ion peak in mass spectrometry (Figure S5) is equivalent to the one obtained for thioether-polyol **2** ([M + Na^+^] = 333) as expected, since they are structural isomers, while the infrared absorption profile (Figure S6) mostly resembles the other monomers, with absorption peaks at 3339 cm^–1^ (O–H stretching), 2928 cm^–1^ (C–H stretching), 1463 cm^–1^ (CH_2_ scissoring), 1380 cm^–1^ (O–H bending), and 1046 and 1008 cm^–1^ (C–O stretching). Yields = 97.5% (**1**), 98.1% (**2**), and 95.0% (3).

### Synthesis of Photocurable Polyesters by Bulk Poly Transesterification

Photocurable polyesters were prepared from the corresponding diols and dimethyl esters by tin-catalyzed bulk poly transesterification. In a typical procedure, dimethyl itaconate (DMI, 1 mol, 158 g) and polyols (2 mol of primary OH groups) were placed in a 500 mL round-bottomed flask equipped with a distillation apparatus and magnetic stirrer under an inert atmosphere. Then, dibutyltin(IV) oxide (DBTO, 5 mmol, 1.24 g) was added, and the mixture was heated to 190 °C for 5 h. Methanol was distilled off during the reaction to drive the poly transesterification equilibrium. Once the reaction was completed, the mixture was cooled to room temperature, dissolved in a minimal amount of ethyl acetate, and precipitated using methanol or ethanol at −80 °C. Alternatively, precipitation could be performed using 20 vol % water in methanol or ethanol at 0 °C, increasing the sedimentation times but improving the energy efficiency of the process. The precipitation step was repeated three times after which 0.1 wt % of 2,6-diterbutyl-4-methylphenol (BHT) was added as a stabilizer. After extensive drying by rotary evaporation and high vacuum, the viscous amorphous polymers were collected in dark containers and stored at +4 °C.

### Synthesis of the Cross-Linker I_2_B_1_–1,4-Butanediyl Bis(methyl itaconate)

The itaconic acid–based cross-linker was synthesized using a transesterification approach similar to the one employed for the synthesis of the photocurable polymers. Typically, dimethyl itaconate (DMI, 474 g, 3.0 mol) and 1,4-butanediol (132 mL, 1.5 mol) were placed in a 1 L round-bottomed flask equipped with a distillation apparatus and magnetic stirrer under an inert atmosphere. Then, dibutyltin(IV) oxide (21 mmol, 5.25 g) was added, and the mixture was heated to 150 °C for 1 h during which the methanol produced by the transesterification reaction was removed by distillation. At the end of the reaction, the mixture was cooled to room temperature, dissolved in 500 mL of ethyl acetate, and washed with water for three times. The organic phase was therefore dried over Na_2_SO_4_ and evaporated to afford 1,4-butanediyl bis(methyl itaconate) as a pale yellow liquid. The evaporated solvent might be collected and reused to increase the sustainability of the process. Yield = 88%. ESI-MS: [M + Na]^+^ = 365.

### Formulation of Photocurable Liquid Resins

All resins were formulated using the same proportions of the different components. One resin was prepared for each synthesized polymer. In a typical procedure, 250 g of resin was prepared by mixing 150 g of poly(ester-thioether), 71.25 g of I_2_B_1_, 25 g of Grindsted Soft-N-Safe (SNS), 1.25 g of ethyl phenyl(2,4,6-trimethylbenzoyl)phosphinate (Et-APO), 0.75 g of 2-isopropylthioxanthone (ITX), and 1.25 g of 2,6-diterbuty-4-methylphenol (BHT). The mixture was homogenized using a planetary mixer (Precifluid PMix100) working at 2800 rpm until all solid components were dissolved.

### Monomers, Polymers, and Resin Characterization

^1^H and ^13^C NMR spectra were obtained on Varian Inova (14.09 T, 600 MHz) and Varian Mercury (9.39 T, 400 MHz) NMR spectrometers. In all recorded spectra, chemical shifts have been reported in ppm of frequency relative to the residual solvent signals for both nuclei (^1^H: 7.26 ppm and ^13^C: 77.16 ppm for CDCl_3_). ^13^C NMR analysis was performed using the ^1^H broad band decoupling mode. Mass spectra were recorded on a micromass LCT spectrometer using electrospray (ES) ionization techniques. ATR-FTIR analysis has been performed using a Cary 630 FTIR spectrometer (Agilent). Rotational viscosity measurements were performed on an Anton Paar MCR102 modular compact rheometer with a CP50–1 geometry, indicating a place-cone geometry with 1° angle and diameter of 25 mm, with a constant rotational frequency of 1 Hz in the temperature range +10/+40 °C and a heating rate of 5 °C/min. Each polymer and formulated resin were analyzed in three independent replicates, and viscosity data are reported as the mean ± SD of the obtained values. Size exclusion chromatography (SEC)/gel permeation chromatography (GPC) was performed on a Knauer system (controlling a Smartline Pump 1000 equipped with a K-2301 refractive index detector). A Shimadzu Shim-Pack GPC-803 column (0.8 cm × 30 cm) and a Shimadzu Shim-Pack GPC-800P (10.0 × 4.6 mm) guard column were used as column systems. HPLC-grade tetrahydrofuran (THF) was used as the eluent with a flow rate of 1 mL/min. The system was calibrated with polystyrene (PS) standards obtained from PSS covering a molar mass range from 300 to 50000 g/mol (Merck).

### Vat Photopolymerization 3D Printing

Once formulated, the resins were poured in the vat of a Peopoly Phenom Prime photopolymerization-based 3D printer working with a 12.5 in. 75 W LCD-LED UV screen (5484 × 3064 resolution, 5.5k HD) and printed into specimens for mechanical tests or other 3D objects. The printer LED screen had a nominal emission spectrum centered at 405 nm.

The g-codes used by the printer for the process were generated by using the slicer software Chitubox Basic 1.9.4 with a layer height of 100 μm and an exposure time per layer of 120 s. All prints were performed at 25 °C. For tensile tests, dog bones were printed according to the ISO 37 Type 2 (75 × 12.5 × 2 mm^3^) specifications. For flexural dynamic mechanical analysis, 50 × 8 × 3 mm^3^ bars were printed. Once printed, all samples were gently detached from the building plate and rinsed in an acetone–isopropanol (1:1) mixture to nonpolymerized resin. Then, the raw 3D-printed objects were postcured for 20 min at room temperature in a curing chamber (Sharebot CURE, wavelength 375–470 nm, 34.7 mW/cm^2^) to ensure complete polymerization of itaconate units.

### Solvent Compatibility of 3D-Printed Materials

To evaluate the stability of 3D-printed materials in different solvents, pieces of 3D-printed material (around 1 g) were placed in different solvents at room temperature (water, NaOH 1 M, iPrOH, EtOAc, acetone, 20 mL). The mass of the 3D-printed object was evaluated at predetermined time steps (0.5 1, 2, 5, 8, and 24 h). The test was repeated three times for the assessment of reproducibility.

### Thermal, Thermomechanical, and Mechanical Characterization of 3D-Printed Materials

Thermogravimetric analysis (TGA, Netzsch TG 209 F1 Libra) was carried out in a nitrogen atmosphere by heating the sample (10–15 mg) at a rate of 20 °C/min from 30 to 700 °C in a platinum crucible. Then, the inert atmosphere was replaced with air to evaluate the inorganic residue after the 30 min isothermal step. Differential scanning calorimetry (DSC) measurements were carried out on a TA Instruments Q2000 DSC modulated apparatus equipped with a refrigerated cooling system (RCS). Samples (10–15 mg) were heated from −88 to 150 °C at 20 °C/min in standard nonhermetic aluminum pans. Dynamic mechanical analysis (DMA) tests were performed using a Netzsch DMA 242 E Artemis instrument in the three-point bending deformation mode (40 mm fixed span support). DMA analyses were carried out between −85 and 150 °C temperature ranges at a 3 °C/min heating rate, 1 Hz frequency, 20 μm amplitude, and 1.5 static force/dynamic force ratio, on three replicate samples for each 3D-printed material. Destructive tensile tests were made by using a Remet TC-10 universal testing machine equipped with a 1 kN load cell at a 1 mm/min cross-head separation rate. Elastic modulus was evaluated as the slope of the linear regression in the 0–0.5% strain. For each tested resin, five replicated measurements were performed, and the extracted elastic moduli, elongations at break, and tensile strengths are reported as their mean ± SD. Hardness of 3D-printed materials was measured using an analogic Shore D durometer (Remet, Bologna, Italy). For each tested specimen, 15 measurements were performed, and the results are expressed as their mean ± SD.

## Results and Discussion

### Synthesis of Thioether Polyols from Terpenes

**Figure 1:**
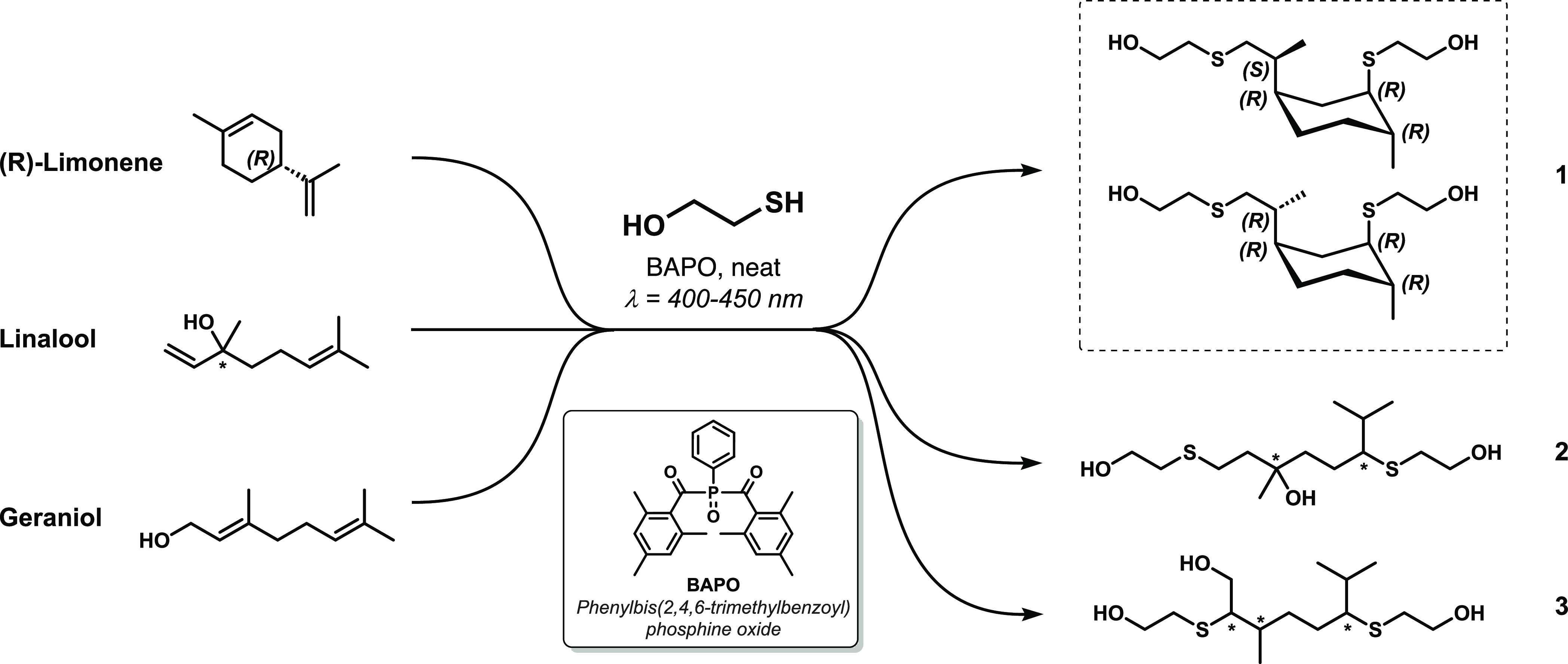
Chemical structure of the starting terpenes and the corresponding thioether-polyols obtained by the addition of 2-mercaptoethanol to their unsaturations. For adduct 1, the major diasteromers obtained are reported in the figure, while for adducts 2 and 3, the asterisks (*) indicate asymmetric carbon atoms present or formed in both configurations.

Terpene-based polyols were synthesized via the thiol–ene addition of 2-mercaptoethanol to the double bonds of terpenes using a simple and photocatalyzed bulk reaction (Figure 1). Compared to cationic and anionic thiol–ene mechanisms, the photoradical approach allows the quantitative conversion of terpenes under mild reaction conditions (room temperature and atmospheric pressure) and low catalyst contents (0.1 mol %), simplifying the polyol purification steps and allowing easy scale-up with improved sustainability.^34^

Because the photocatalyst is soluble in the reaction mixture, the reaction proceeds without a solvent, even when reactants are not miscible (e.g., limonene). The light absorbed by the photocatalyst forms radical initiator species that remove a hydrogen atom from thiol, forming thiyl radical species (RS·), which in turn react with the double bonds of terpene molecules. The radical mechanism leads to the formation of all possible diastereomers in different proportions,^35^ and all diastereomers can form photocurable poly(ester-thioether)s with dimethyl itaconate (DMI). Because this study is not focused on studying the effect of the stereochemistry of monomers on the properties of final materials, the stereochemistry was not controlled and different formed components were not separated, affording an easy, efficient, and scalable approach to incorporate terpenes in biobased resins for vat photopolymerization. Geraniol, limonene, and linalool were selected from the numerous naturally available terpenes because of their high availability and structural differences, which allow the preparation of poly(ester-thioether)s with different chemical and structural features (Figure 1). These terpenes were selected because, when polycondensed with linear diacids or diesters, their poly(ester-thioether)s exhibit different structural features. Notably, **1** leads to linear poly(ester-thioether)s with a cyclohexane ring in the polymeric chain, **2** leads to linear poly(ester-thioether)s with a polar tertiary OH group that exhibits hydrogen bonding (H-bonding), and **3** allows the synthesis of branched poly(ester-thioether)s.

Limonene is a chiral, enantiopure, and naturally occurring terpene, with a cyclohexene ring bearing an isopropenyl substituent. Because of the very low polarity of limonene, it is poorly miscible with 2-mercaptoethanol; therefore, its reaction with 2-mercaptowthanol starts slowly. However, once enough product is formed, the three-component mixture becomes homogeneous and both unsaturations easily react with thiyl radicals, forming thioether **1**, as previously reported.^31^ The thiyl radical preferentially attacks the less hindered carbon atom of C=C bonds because of steric and electronic effects; therefore, the major regioisomer is expected to exhibit the structure shown in Figure 1.^36^ Additionally, **1** bears four asymmetric carbon atoms, one of which has a fixed configuration because of the enantiopurity of the starting material. The chemical structures of all possible diastereomers are shown in Figure S7. The product mixture was analyzed via ^1^H, ^13^C, and ^1^H–^1^H correlation spectroscopy (COSY) and ^1^H–^13^C heteronuclear single quantum coherence (HSQC) NMR spectroscopy (Figures S8–S10), and NMR results confirm the regioselectivity of the reaction toward the formation of the expected product. The complete assignment of the detected NMR signals is shown in Table S1. LeBel at al. previously reported that, especially in the presence of excess thiol, the radical thiol–ene addition of substituted methylcyclohexenes predominantly forms *trans*-diaxial adducts; therefore, the formation of two diastereomers of **1** possessing this configuration may be preferred.^37^ A detailed analysis of NMR spectra shows the splitting of only those signals related to protons close to the original stereocenter of limonene, supporting the hypothesis that the mixture is composed of two *trans*-diaxial diols. However, small amounts of other regioisomers and diastereomers (<1%) are detected in the monomer mixture. Obtained thioether polyol **1** allows the synthesis of linear polyesters with 1,5-disubstituted 2-methylcyclohexane rings along the polymeric chain.

Linalool molecules bear a tertiary alcohol moiety, one monosubstituted C=C bond, and one trisubstituted C=C bond. Their reaction with 2-mercaptoethanol forms **2**, a polyol bearing two primary and one tertiary alcohol functionalities. The polarity of its tertiary alcohol moiety makes linalool miscible with 2-mercaptoethanol, leading to high reaction rates and formation of triol **2** as the reaction product. Similar to the synthesis of **1**, the thiyl radical preferentially attacks the less hindered carbon atom of the C=C bonds. From a stereochemistry perspective, linalool bears one asymmetric carbon atom, and it is commercially available as a racemic mixture of enantiomers. The thiol–ene addition of 2-mercaptoethanol of linalool forms one additional chiral carbon atom; therefore, the product mixture comprises two diastereomers of product **2** with no foreseeable preference of one over the other (Figure S11). Such predictions of the structure of the thiol–ene product were confirmed via NMR spectroscopy (Figures S12–S14), and NMR spectra show the diastereotopic isopropyl pattern, confirming the regioselectivity of the addition reaction. Moreover, all ^1^H and ^13^C NMR signals are doubled in the NMR spectra of product **2**, suggesting the presence of both of its diastereomers in similar concentrations. The complete assignment of the detected NMR signals is shown in Table S2. Thioether polyol **2** was selected because it forms linear polyesters whose macromolecules interact with each other via H-bonding because their tertiary alcohol moiety is inert toward polycondensation due to steric hindrance.

Geraniol, a structural isomer of linalool, was selected because it has a primary OH group and two aliphatic unsaturations. Like linalool, geraniol is miscible with 2-mercaptoethanol, but the reaction rate is really low when 1 mol % photocatalyst is used. In fact, after 48 h of reaction, the conversion using 1 mol % phenylbis(2,4,6-trimethylbenzoyl) phosphine oxide (BAPO) measured via NMR spectroscopy is still <20%. This may be due to the presence of the allylic alcohol functionality on the geraniol molecule, which can trap radical species, forming stable and delocalized allyloxy radicals.^38^ However, this issue can be solved by increasing the photocatalyst concentration to 10 mol %. Both double bonds in the geraniol structure are trisubstituted, and it is reasonable to expect that the RS· species would preferably attack the less hindered carbon atoms, similar to that previously discussed for other monomers. Because the reaction forms a product bearing three asymmetric carbon atoms, four different diastereomers may be produced (Figure S15). NMR analysis confirms the proposed chemical structure of product **3** and the presence of four diastereomers in the monomer mixture because all NMR signals are quadrupled (Figures S16–S18). The complete assignment of the detected NMR signals is shown in Table S3. Unlike linalool-derived polyol, thioether-triol **3** bears three primary alcohol moieties that are equally reactive, forming branched polyesters when polymerized with DMI. The differences in reactivity of the three terpenes in thiol–ene addition are worth noting. While full conversion of the reagents is achieved in 6 h during the synthesis of thioether diol **1**, the reaction time increases to 48 h for **2** and 120 h for **3**. This is due to the alcoholic nature of linalool and geraniol, which can trap free radicals formed during the photolysis of the radical initiator, forming, respectively, a stable tertiary alkoxy radical and an even more stable allylic one, decreasing the abundance of reactive thiyl radicals.^39^

### Synthesis of Photocurable Polyesters via Bulk Poly Transesterification

**Figure 2:**
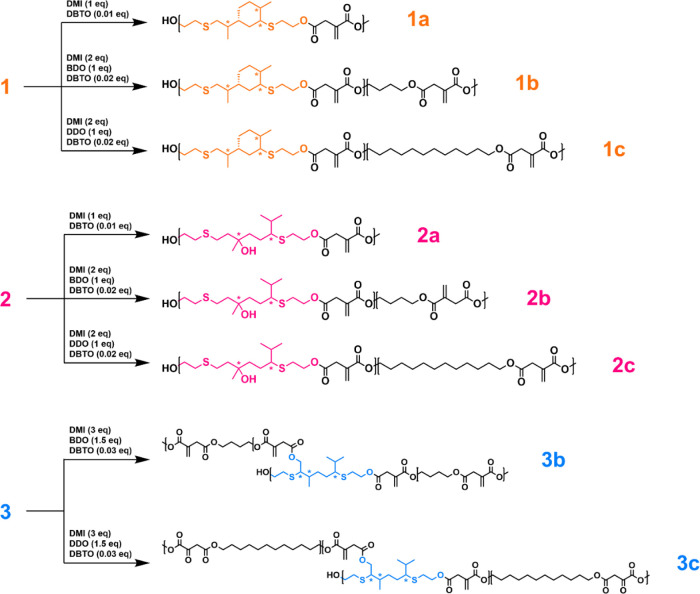
Chemical structure of the photocurable poly(ester-thioether)s. Reagents stoichiometry is referred to as one equivalent of thioether-polyol. Reaction conditions are for all polymerization reactions: N_2_ atmosphere and 190 °C for 5 h during which coproduced methanol is removed by distillation. DMI = dimethyl itaconate, BDO = 1,4-butanediol, DDO = 1,12-dodecanediol, and DBTO = dibutyl tin(IV) oxide. Asterisks (*) highlight asymmetric carbon atoms present or formed in both configurations.

**Figure 3:**
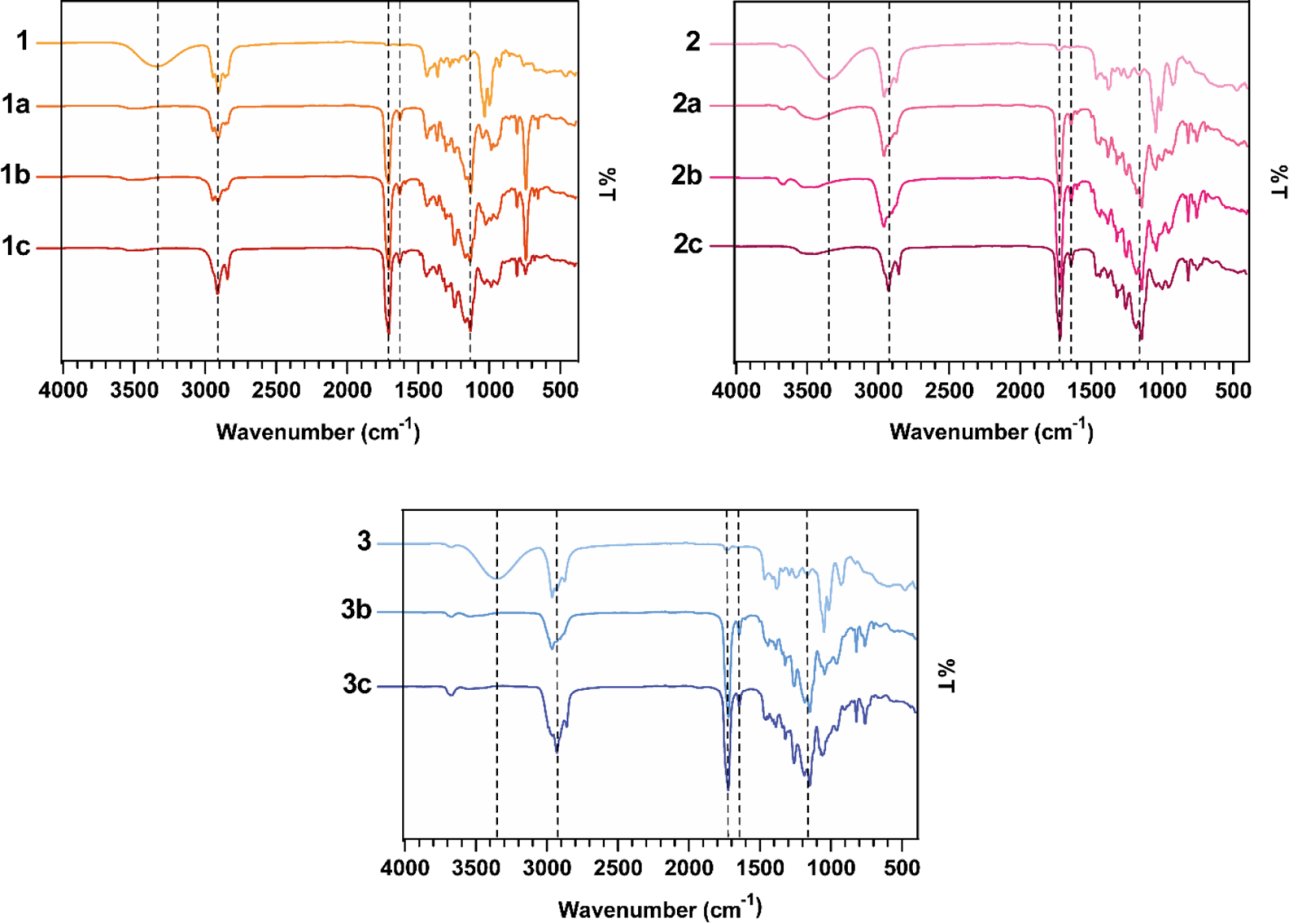
ATR-FTIR spectra of the poly(ester-thioether)s compared to the corresponding thioether-polyol monomers. Dashed lines correspond in all spectra at the wavenumbers 3350, 2930, 1730, 1640, and 1145 cm^–1^, from left to right.

Photocurable poly(ester-thioether)s were synthesized via the poly transesterification of terpene polyols with DMI. Dibutyl tin(IV) oxide was used as the catalyst because of its high transesterification efficiency, which has been widely explored in the literature, particularly during the synthesis of polyesters.^40^ The catalytic mechanism of DBTO is shown in Figure S19. Notably, the use of DBTO for the poly transesterification of dimethyl esters with diols allows the recovery of released methanol via distillation, offering the chance to recycle methanol for the synthesis of new DMI from biobased itaconic acid and reducing the impact of monomer synthesis from a green chemistry perspective. Using this approach, thioether polyols were polymerized with only DMI or diluted with biobased aliphatic α,ω-diols with different chain lengths, such as 1,4-butanediol (BDO) and 1,12-dodecanediol (DDO), to study the effect of copolymerized linear aliphatic chains on the properties of 3D-printed materials. In addition to the classical polycondensation routes, researchers have proposed alternative enzymatic processes, but, to date, experimental conditions hinder process scalability, and the obtained monomer conversions are still quite limited compared to those obtained via traditional high-temperature processes.^41^ Three poly(ester-thioether)s were synthesized using thioether polyol **1**, named **1a**, **1b,** and **1c**, and three additional poly(ester-thioether)s were synthesized using thioether polyol **2**, called **2a**, **2b,** and **2c**. In the case of monomer **3**, polymerization occurred without the addition of linear diols and formed a polymer network with extensive branching. This network was not soluble in organic solvents and could not be formulated into 3D-printable resins. Therefore, only two polymers were prepared using thioether polyols **3**, called **3b** and **3c**, using BDO and DDO as alcoholic comonomers, respectively. For polymerization to occur, an equimolar amount of hydroxyl and ester groups are required because an excess of diester or polyol considerably impedes the increase in the molecular weight of macromolecules. Therefore, the molar ratio of monomers (thioether polyol/linear diol/DMI ratio) was adjusted to 1:0:1 for **1a** and **2a** polymers, 1:1:2 for **1b**, **1c**, **2b,** and **2c** polymers, and 2:3:6 for **3b** and **3c** polymers. The chemical structures of the synthesized poly(ester-thioether)s are shown in Figure 2. All poly(ester-thioether)s were first characterized via ^1^H, ^13^C, and HSQC NMR spectroscopy, showing the effective incorporation of all monomers in polymeric chains while conserving their relative molar ratios and the integrity of the photocurable moiety of itaconic acid and the thioether polyol monomer structure (Figures S20–S35). As expected, NMR signals of all polymers are broader and less resolved than those of free monomers, especially when different polyols are used. However, for all polymers the overall spectral features are consistent with the NMR spectra of free monomers, except for the shift of the – CH_2_OH peaks to higher values related of its successful esterification with itaconic acid. NMR signals of terminal monomers can be easily identified using HSQC NMR spectroscopy, revealing the presence of hydroxyl and methyl ester end groups, as expected. Moreover, NMR analysis of linalool-containing poly(ester-thioether)s **2a**, **2b**, and **2c** confirms the prediction that the tertiary OH group of linalool could not participate in the polymerization reaction due to steric hindrance. Its esterification leads to the deshielding of the NMR signal related to the close quaternary carbon, which is not observed in any ^13^C NMR spectra. Additionally, Fourier transform infrared (FTIR) spectroscopy was used to further confirm the predicted polymer structures. The vibrational peaks of the abundant ester carbonyl stretching (strong band at 1730 cm^–1^), C=C stretching of the photocurable functionalities of itaconic acid (weak band at 1640 cm^–1^), and aliphatic CH_2_ groups (medium band at 2930 cm^–1^) are observed in the FTIR spectra of all polymers (Figure 3). Compared with the FTIR spectra of the corresponding thioether polyol, the FTIR spectra of all polymers exhibit an additional band at 1145 cm^–1^, which is attributed to the C–O stretching vibration of ester moieties. The alcoholic O–H stretching absorption is clearly visible in the FTIR spectra of thioether polyols **1** and **3**, but it is absent in the FTIR spectra of the corresponding polymers. In the FTIR spectra of the polymers of **2**, the O–H stretching band (∼3350 cm^–1^) has a lower intensity and moves to higher vibration frequencies compared to the corresponding monomer **2**. This further confirms the presence of free tertiary OH groups on monomers derived from product **2**, and the shift to higher frequencies may be due to the transition from intramolecular to intermolecular H-bonding. Finally, in the FTIR spectra of the polymers, the absence of the high-intensity absorption bands in the 1300–1350 and 1000–1070 cm^–1^ regions indicates little to no oxidation of thioether moieties to sulfone or sulfoxide. More differences can be observed in the fingerprint region (500–1100 cm^–1^) in the FTIR spectra of monomers and polymers; however, the unambiguous assignment of such spectral features is not possible because they are related to the vibration of abundant C–C, C–H, and C–S bonds.

Gel permeation–size exclusion chromatography (GPC-SEC) was employed to assess the molecular weight distribution of poly(ester-thioether)s (Table 1), and their viscosity was measured between 10 and 40 °C for a comprehensive comparison (Figure S36). For molecular weight analysis, all polymers were characterized by a low polymerization degree with an average number molecular weight (*M*_n_) between 1000 and 2000 g/mol. Higher-molecular-weight polymers would have led to increased viscosities and, therefore, to a difficult processability of the liquid resins through 3D printing.

**Table 1:** Molecular Weight Distributions, Polydispersity Index (PDI), and Rotational Viscosity (η) at 25°C of the Synthesized Poly(ester-thioether)s^a^

polymer	*M*_n_ (g/mol)	*M*_w_ (g/mol)	PDI	η at 25 °C (Pa s)
**1a**	1750 ± 130	2850 ± 255	1.6 ± 0.1	48.4 ± 2.9
**1b**	1800 ± 145	5050 ± 215	2.8 ± 0.2	33.9 ± 3.6
**1c**	1400 ± 140	5550 ± 320	4.0 ± 0.2	16.7 ± 1.8
**2a**	2200 ± 250	3000 ± 230	1.4 ± 0.1	16.1 ± 1.4
**2b**	1700 ± 180	4600 ± 415	2.7 ± 0.1	10.7 ± 1.7
**2c**	1800 ± 215	6700 ± 380	3.8 ± 0.2	4.43 ± 0.6
**3b**	1800 ± 160	4450 ± 395	2.5 ± 0.1	24.0 ± 2.0
**3c**	1850 ± 190	9600 ± 855	5.2 ± 0.2	31.1 ± 4.2

a Data are expressed as the mean ± SD obtained by GPC-SEC analysis of three independent batches for each poly(ester-thioether).

**Figure 4:**
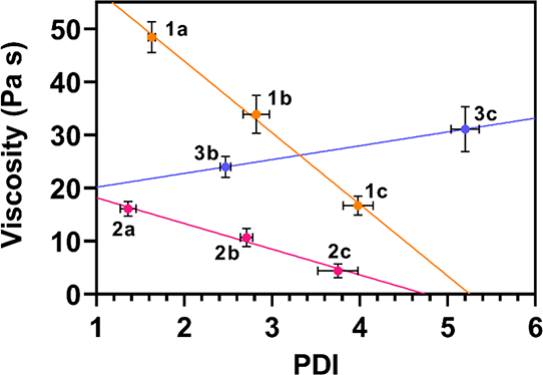
Measured viscosity at 25 °C for the synthesized poly(ester-thioether)s as a function of their polydispersity index (PDI) calculated from GPC data. Error bars represent the standard deviations reported in Table 1.

To assess the reproducibility of the proposed polymerization process, all polymers were prepared three times with the same procedure and subjected to independent molecular weight and rheological analysis. The obtained *M*_n_, *M*_w_, polydispersity index (PDI), and viscosity values show standard deviations ranging from 2 to 12% of the average values, implying homogeneity among different synthesis batches and good reproducibility of polymer syntheses. All as-prepared polymers exhibit viscosities in the 5–50 Pa s range, and the viscosity of all polymers drastically decreases with an increase in the temperature, with an average 25-fold reduction in polymer viscosity when the temperature increases from 10 to 40 °C. Figure 4 shows the viscosity of poly(ester-thioether)s at 25 °C as a function of their PDIs. In all cases, the PDI increases when two polyols are used as monomers, and the most relevant increase in the PDI is observed when BDO is replaced with DDO. The viscosity of linear poly(ester-thioether)s decreases when BDO is used as the comonomer (polymers **1b** and **2b**), and a further decrease in viscosity is observed when BDO is replaced with DDO (polymers **1c** and **2c**). This effect may be due to the corresponding increase in the PDI because broader molecular weight distributions usually lead to lower melt viscosities because of the presence of low-molecular weight chains that act as plasticizers.^42^

Additionally, linear aliphatic chains of BDO and DDO may reduce intermolecular interactions, particularly in the polymers of **2** in which the thioether polyol unit can form H-bonds. Among the branched polymers of **3**, DDO-containing polymer **3c** exhibits a viscosity higher than **3b**. This is probably due to intermolecular effects of the branched polymeric structure, which overcomes the general decrease in viscosity observed for high-PDI linear poly(ester-thioether)s. Nevertheless, all as-prepared poly(ester-thioether)s are characterized by the optimal rheological features for their formulation into photocurable resins for applications in vat photopolymerization.

### Synthesis of the Cross-Linker 1,4-Butanediyl Bis(methyl itaconate) (I_2_B_1_)

**Figure 5:**
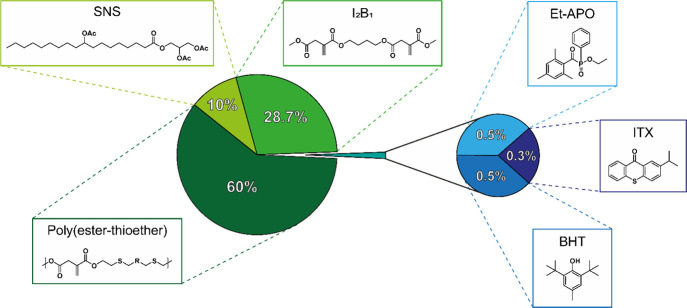
Weight composition of the poly(ester-thioether)-based formulations for VAT photopolymerization with the chemical structures of the resin’s components.

Because of the high viscosity of most synthesized poly(ester-thioether)s, a low-molecular-weight reactive diluent was prepared via the transesterification of two molecules of DMI with one molecule of BDO to obtain a biobased difunctional cross-linker. The reaction was performed in an analogous manner, similar to that for the synthesis of the polyester. However, by changing the ratio of alcoholic and ester functionalities, it is possible to completely prevent the increase in the molecular weight. ^1^H NMR spectroscopy confirms the expected structure of the product (I_2_B_1_ in Figure 5), and the splitting of the terminal methyl groups into two identical peaks reveals the fully random orientation of itaconate units (Figure S37). The low-molecular-weight cross-linker is a liquid at room temperature with a measured viscosity of 0.076 Pa s at 25 °C (Figure S38).

### Resin Formulation and Vat Photopolymerization

Synthesized poly(ester-thioether)s were mixed with the bifunctional cross-linker I_2_B_1_, plasticizer 9-hydroxystearic acid monoglyceride triacetate (Grindsted Soft-N-Safe; SNS), radical photoinitiator ethyl phenyl(2,4,6-trimethylbenzoyl)phosphinate (Et-APO), polymerization inhibitor 2,6-di-*tert*-butyl-4-methylphenol (BHT), and photosensitizer 2-isopropylthioxanthone (ITX) according to the proportions reported in Figure 5 to obtain a methacrylate-free liquid resin that can harden when exposed to radiation ranging between 380 and 420 nm, covering the nominal spectral range emitted by the screen of 3D printers. In fact, the FTIR spectrum of the selected photoinitiator exhibits an absorption peak centered at 370 nm and spreading up to 410 nm, and the photosensitizer ITX is characterized by light absorption between 380 and 415 nm.^43,44^ SNS, mainly composed of 9-hydroxystearic acid monoglyceride triacetate, was selected as a plasticizer because it can be obtained from naturally occurring castor oil and reduce the oxygen permeability of the polymer matrix, supporting the photopolymerization process by reducing the concentration of radical-inhibiting oxygen triplet species.^45^ Moreover, test prints performed without the addition of any plasticizers formed brittle materials that broke as the cured material was detached from the build plate. The radical photoinitiator was selected from the family of acyl phosphine oxides, which are characterized by broad absorption bands in the near-UV range up to 420 nm and efficient degradation of their photoexcited states into reactive RS.^46^ In particular, compared to commonly used BAPO and diphenyl(2,4,6-trimethylbenzoyl) phosphine oxide (MAPO), which are solids at room temperature, Et-APO was selected because it is liquid at room temperature, allowing easier mixing with resin components and improved solubility.

BHT was added to spatially control radical polymerization, increasing the 3D printing resolution. In fact, during the printing process, the hindered phenol moiety could trap reactive thiyl radicals present outside the irradiated regions of the vat containing the photocurable resin (by diffusion of thiyl radicals or reflection/diffusion/scattering of UV light), forming stable phenoxy radicals and stopping chain polymerization.^47,48^ However, in the irradiated portions of the resin, the concentration of formed thiyl radicals is high enough to saturate all free BHT molecules and polymerization effectively occurs. Finally, ITX was included as a UV photoabsorber because it can reduce the penetration depth of UV light into the resin, limiting the formation of free radicals outside the irradiated regions. In addition, according to the literature, when used as a photosensitizer, ITX releases the absorbed energy to the initiator, increasing the concentration of reactive initiating radicals.^49,50^ Therefore, ITX synergistically acts with BHT to improve the overall printing resolution and Et-APO to increase the concentration of active radical species.

The viscosity of photocurable resins was measured between 10 and 40 °C to assess whether the formulation of poly(ester-thioether)s with other components forms liquid mixtures compatible with the photopolymerization-based printing process. The full viscosity versus temperature curves is shown in Figure S39, while the interpolated viscosities at 25 °C are shown in Table 2.

**Table 2:** Rotational Viscosity Measured for the Resins Formulated with the Different Poly(ester-thioether)s^a^

resin	**r1a**	**r1b**	**r1c**	**r2a**	**r2b**	**r2c**	**r3b**	**r3c**
η at 25 °C (Pa s)	8.9 ± 0.8	2.5 ± 0.3	1.3 ± 0.6	2.9 ± 0.6	1.1 ± 0.3	0.7 ± 0.2	2.8 ± 0.5	3.0 ± 0.6

a Data are expressed as the mean ± SD, obtained by analyzing three independently formulated samples.

The resin formulated using polymer **1a** was named **r1a**, and other resins formulated using other polymers were similarly named according to the name of the corresponding poly(ester-thioether). As expected, the viscosities of formulated resins exhibit similar trends to the viscosities of poly(ester-thioether)s because poly(ester-thioether)s are the major components of resin mixtures and also have the highest viscosities among the mixed materials. Nonetheless, the formulation with low-viscosity I_2_B_1_ and SNS reduces viscosities of all formulated resins in the 0.25–10 Pa s range, which is the optimal viscosity range for the 3D printing process, as reported in the literature.^51^ High-viscosity resins often cause printing errors and reduce resolutions due to the slower diffusion of fresh liquid resin below the build stage of the 3D printer between one layer and the next. Therefore, low-viscosity resins are often preferentially used during this additive manufacturing technique. In fact, many vat photopolymerization printer manufacturers include a heating system that heats the resin to 30–35 °C, reducing its viscosity and improving the overall quality of the 3D printing process in terms of the printing resolution and time. To understand the biobased content of each formulation, a quick analysis of the derivation of each component is herein presented. Itaconic acid is a promising 100% biobased building block, as discussed in Introduction, and BDO can be considered as fully biobased because it is efficiently manufactured via the direct fermentation of sugars. On the other hand, DDO may be obtained via the catalytic reduction of ω-oxidated lauric acid.^52,53^ Furthermore, SNS is a biobased plasticizer produced via the acetylation of naturally abundant castor oil. Obviously, 2-mercaptoethanol is not biobased, and similar conclusions can be drawn for Et-APO, BHT, and ITX, whose production is fully petrochemical. The biobased content was evaluated in terms of the TUV Austria – OK BIOBASED labeling, which allows for the assignment of a rank from one to four stars to the individual compounds that conform to the EU norm ‘NPR-CEN/TS 16137:2011’ on the determination of biobased carbon content.^32,33^ For organic materials, the assessment of the biobased carbon content is generally preferred over the total biomass content because the measurement technique for the former is easier (based on ^14^C radioisotope analysis). The complete calculation of the biobased carbon content of each formulation is reported in the Supporting Information, and the biobased carbon content values (which represents the percentage of biobased carbon in the formulation with respect to the overall carbon content, ) and classical overall biomass content values (which represents the percentage of the total mass of the formulation that is derived from biobased feedstock, *m*_B_) are shown in Tables 3 and S4, respectively.

**Table 3:** Quantitative Parameters for the Classification of the Biobased Content of the Formulated Photocurable Resins^a^

resin		*m*_B_
**r1a**	85.7%	74.5%
**r1b**	89.8%	82.3%
**r1c**	91.6%	85.0%
**r2a**	85.9%	75.5%
**r2b**	89.8%	82.8%
**r2c**	91.6%	85.3%
**r3b**	91.5%	85.8%
**r3c**	93.2%	88.2%

a Details regarding the calculation of the reported parameters are available in the Supporting Information.

The results presented in Table 3 show that more than 85% of the carbon atoms in the described formulations could, in theory, derive from biomasses, a potential assignment of the four-star ranking conferred by the TUV Austria certification body for materials with a total biobased carbon content exceeding 80%. Nonetheless, all formulations are characterized by at least 74.5% total biomass contents, which is among the highest reported values for a (meth)acrylate-free photocurable resin for vat photopolymerization. Then, the formulated resins were efficiently used for the additive manufacturing of 3D objects. In addition to the specimen required for the mechanical characterization of prepared materials, 3D objects such as chess pieces and complex abstract structures were printed to determine the high printing resolution that may be obtained using reported resins (Figures S40, S41, and 6).

**Figure 6:**
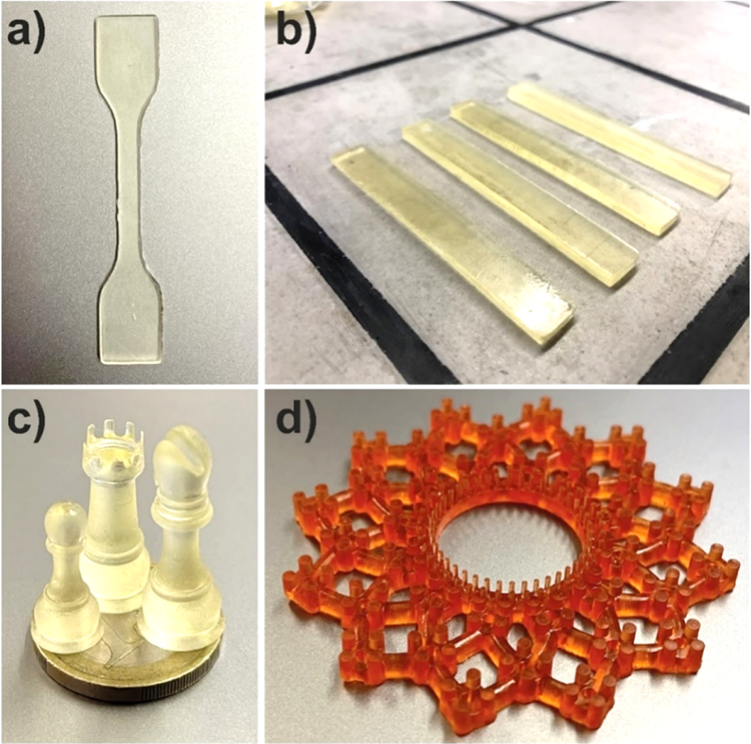
Pictures of 3D-printed materials. (a) Tensile and (b) DMA test specimen, (c) small chess pieces, and (d) complex abstract structure with small details printed after the addition of 0.05 wt.% purpurin to the resin. Chess pieces were placed on top of a 1 € coin to highlight their small size. All displayed prints were performed using the **r1a** resin to show the high resolution that can be obtained with the proposed poly(ester-thioether)s. Specimens printed with the other resins are not displayed, but no appreciable differences in the printing resolution were observed when the poly(ester-thioether) was changed.

To quantitatively evaluate the spatial accuracy and resolution of the printing process, the dimensions of the smaller sections of five tensile test specimens of each formulation were measured and compared to those of the corresponding virtual 3D models (Table S5), revealing good spatial accuracy for all 3D-printed materials. A slight deviation from the computer model is detected in the *z*-direction and along the *x*–*y* plane (0.05 ± 0.01 mm along the *z*-axis and 0.05 ± 0.02 mm on the *x–y* plane), probably due to the imperfect calibration of the printer. The high printing accuracy achieved is perfectly consistent with those achievable using commercially available (meth)acrylate-based formulations.^54^ Notably, printing accuracy is related not only to the printability of the resin but also to printing parameters, which need to be optimized for each printer–resin pair. Furthermore, the possibility of coloring the fabricated resins using natural organic dyes such as purpurin in very low concentrations (∼0.05 wt %) was explored. The addition of purpurin to **r1a** resin did not affect the latter’s printability and allowed the manufacturing of colored specimens with high resolutions (Figure 6d).

In addition, the stability of prepared resins after 6 months of storage was assessed at room temperature (25 °C) and +4 °C, showing that no spontaneous polymerization occurs during storage, probably due to the presence of BHT, which acts as the radical stabilizer. Moreover, the printability of prepared resins is not affected by prolonged storage. The use of itaconates for vat photopolymerization often requires higher UV exposure times per layer to ensure resin hardening. This is because itaconate radicals exhibit lower reactivity than meth(acrylates) caused by the higher hinder around the photocurable moiety in itaconic acid molecules.^55^ In fact, reported resins required around 5–10× higher printing times than commercially available resins during vat photopolymerization, but this issue is not limiting and may be solved in the future by manufacturing 3D printers with higher UV light powers or by introducing radical polymerization catalysts that speed up the hardening process of resins upon UV exposure. However, lower radical reactivity can be associated with the lower polymerization rate, leading to a reduced shrinkage of 3D-printed materials. In fact, the shrinking phenomenon, which is sometimes evident and problematic when printing (meth)acrylate-based formulations,^56^ does not occur when as-prepared formulations are used, as suggested by the printing accuracies shown in Table S5.

Attenuated total reflectance (ATR)-FTIR spectroscopy of liquid photocurable resins and printed 3D materials was performed to explore the changes in the main functional groups that occur during the 3D printing process (Figures S42–S44). For all samples, the spectra recorded for the photocured materials retrace the ones recorded for the corresponding liquid resin with the exception of three peaks of the C=C moiety of itaconic acid (C=C stretching at 1640 cm^–1^, C = C–H bending at 850 and 760 cm^–1^), which are strongly reduced in the FTIR spectra of photocured samples. This suggests the effective photopolymerization of most itaconate units, while all other functional groups are preserved. Furthermore, extensive peak broadening can be observed in the fingerprint region (800–1500 cm^–1^) after photocuring in the FTIR spectra of all samples, implying the occurrence of increased chemical interactions in the photocured network and formation of new C–C bonds.

It is reasonable to expect that not all itaconic acid monomers are involved in photopolymerization during 3D printing. At the beginning of the photocatalyzed radical polymerization, itaconic acid units start reacting randomly with other itaconate units from other polymeric chains or from I_2_B_1_. As polymerization proceeds, the irradiated region gets more and more viscous due to the reduced mobility of polymeric chains, up to the point where the cross-linked network hardens and the movement of polyester chains is totally prevented. At this point, unreacted itaconic acid monomers between two cross-linking junctions are cannot move around and collide with other itaconic acid groups to continue polymerization. The overall effect of this type of polyester photocuring is the formation of distinct polymer domains characterized by different abundances of unsaturated itaconate blocks with respect to the photocured itaconate residues. This is one of the main molecular-level differences in the current approach and most other photopolymerization approaches reported in the literature. In fact, most commercial resins are comprise nonphotocurable polymers that are functionalized with photocurable end groups, often diluted with small-molecule (meth)acrylates. In both cases, during and after the cross-linking and hardening of resins, all photocurable groups can move to a certain extent and complete the polymerization process because they are either the only photocurable group of a small molecule or terminal photocurable functionality in a flexible polymeric chain.

### Solvent Compatibility of 3D-Printed Materials

The stability of 3D-printed materials containing synthesized poly(ester-thioether)s was evaluated by measuring their weight variation after 24 h immersion in different solvents such as water, 1 M NaOH in water, 2-propanol (iPrOH), ethyl acetate, and acetone. The change in sample weight with time grouped by the type of resin tested (Figures S45 and S46) and by the used solvent (Figure S47) is reported in the Supporting Information. Moreover, the performances of the described resins were compared with those of a popular commercial analogue, Tough resin developed and distributed by Formlabs. All materials exhibit good stability in polar environments, such as water, 1 M NaOH, and iPrOH, with a change in sample mass of ±1% during 24 h of immersion. On the other hand, when placed in ethyl acetate, the weight of all materials increases to +10% of the initial mass after 24 h of immersion. The immersion in NaOH leads to only minor weight loss, suggesting the good stability of poly(ester-thioether)s against hydrolysis at room temperature. Interestingly, the immersion of 3D-printed materials in acetone lead to a gradual mass loss for some resins reaching values of −20% (resins r1b-3D, r2a-3d, r2b-3D, and r3b-3D) while for the rest of the tested materials, it led to a slight mass gain, less relevant than the one recorded for ethyl acetate. The observed loss in weights of resins after immersion in acetone may be partially related to the diffusion of noncross-linkable components in the solvent (accounting for a mass loss of −10% maximum) and partial disruption of the 3D-printed piece. Poly(ester-thioether)-based formulations exhibit better compatibility with all solvents than their commercial counterpart.

### Thermal, Thermomechanical, and Mechanical Properties of 3D-Printed Resins

3D-printed materials were thermally, thermomechanically, and mechanically characterized to thoroughly characterize their behavior. All materials exhibit similar thermal stability: resins are stable until 150–160 °C and a slight weight loss of 1–3% occurs (onset 204 °C–230 °C) before the main degradation step at 300–500 °C (onset 312–342 °C), leading to a residual mass of 9–12% in an inert atmosphere (Figure S48). When switched to the air atmosphere, the weight of the final residue is negligible (0.5–0.8%). The slight change in the slope of all thermogravimetric analysis (TGA) curves observed at ∼200 °C can be attributed to the release of water molecules coordinated with the carboxyl moiety from DMI terminal units.^57^ The thermal degradation of macromolecules begins well after 300 °C, guaranteeing that all obtained thermosets are highly thermally stable, regardless of the particular poly(ester-thioether) resin formulation. While the onset of the macromolecular degradation (2nd loss onset in Table 4) is quite similar for different resins, the presence of the aliphatic carbon chain affects the degradation rate, that is, the degradation rate decreases with an increase in the chain length (all resin series exhibit this degradation pattern, as observed in the thermograms shown in Figure S48). The obtained thermal degradation profile resembles those of many (meth)acrylate- and itaconate-based photopolymers obtained via the vat polymerization of commercially available resin and described in the literature, which are characterized by the first loss onset temperature ranging between 150 and 250 °C and second loss onset temperatures ranging between 300 and 400 °C.^22,25,58^

**Table 4:** Thermal and Thermomechanical Properties of 3D-Printed Resins

sample	1st loss onset^a^ (°C)	2nd loss onset^a^ (°C)	residue in N_2_ @ 700 °C^a^ (%)	residue in air @ 700 °C^a^ (%)	1st *T*_g_^b^ (°C)	2nd *T*_g_^b^ (°C)	*E*′ loss onset^c^ (°C)	*E*′ @ 25 °C (MPa)
**r1a-3D**	230	332	11.3	0.5	–20	53	–52	999 ± 42
**r1b-3D**	214	336	11.4	0.7	–28	54	–49	850 ± 37
**r1c-3D**	221	342	9.3	0.5	–30	57	–57	445 ± 51
**r2a-3D**	214	312	11.4	0.8	–13	54	–50	708 ± 62
**r2b-3D**	215	318	12.4	0.6	–19	53	–54	1175 ± 84
**r2c-3D**	216	316	10.7	0.7	–20	51	–20	584 ± 38
**r3b-3D**	204	324	12.1	0.7	–17	53	–17	699 ± 35
**r3c-3D**	209	329	9.4	0.5	–31	51	–31	398 ± 22

a From TGA.

b From DSC.

c From DMA. The standard deviations of *E′* loss onset values are lower than 2 °C for all samples.

The differential scanning calorimetry (DSC) thermograms (Figure S49) exclude the additional thermal-activated cross-linking of 3D-printed materials because the absence of exothermic events indicates that photoactivated cross-linking during the printing process can cure resins to the maximum achievable extent. Their overall thermal behavior is comparable; in particular, materials are characterized by two *T*_g_s: one at low temperatures between −13 and −31 °C and the second between 51 and 57 °C. The presence of two well-separated glass transitions (*T*_g_s) suggests some sort of phase compartmentalization and/or the presence of a blocky structure in original linear copolymers. The lack of any notable endothermic signal is consistent with FTIR and DSC analyses, showing that the number of itaconate residues that can be polymerized, either thermally or photoradically, is limited. The first glass transition, well below room temperature, increases the strength of the material due to the rubbery behavior of the polymeric fraction at temperatures above *T*_g_, reducing the brittleness typical of thermosetting systems. The low *T*_g_ is affected by the presence of the linear aliphatic chains derived from diols, BDO, or DDO, incorporated in poly(ester-thioether)s, supporting the hypothesis that the lower *T*_g_ is determined by the polyester structure. Indeed, when present, such additional carbon chains may afford higher system mobility due to an increase in free volume, resulting in a lower *T*_g_. Such effect is clearly observed for the formulation created using thioether polyol **1** (Figure S49a and Table 4): **r1a-3D** exhibits the low *T*_g_ at −20 °C, whereas **r1b-3D** and **r1c-3D** exhibit the low *T*_g_ at −28 and −30 °C, respectively. Even in the case of thioether polyol **3**, DDO considerably lowers the *T*_g_ to −31 °C (Figure S49c and Table 4), and the effect of BDO and DDO on the *T*_g_ is less pronounced in resins fabricated using thioether polyol **2** (Figure S49b and Table 4), probably due to the formation of an H-bond using OH groups.

The thermomechanical behavior of 3D-printed materials was evaluated via dynamic mechanical analysis (DMA; Figure S50). An analysis of the *E′* curves shows that all materials exhibit a similar overall trend, typical of unreinforced cross-linked resins: the storage modulus, accounting for the material stiffness, is high at low temperatures (below *T*_g_) and decreases as the temperature increases, exceeding *T*_g_. The *E′* profiles confirm, at least partially, the rubbery behavior of the printed materials at room temperature, which is consistent with DSC analysis. The onsets of the reduction in *E′* are comparable (between −57 and −49 °C), but some differences can be found in the *E′* trends: poly(ester-thioether)s containing long aliphatic carbon chains derived from DDO exhibit a “softer” behavior than the other two with BDO or without the addition of a linear diol. Indeed, in all cases, the *E′* profile of DDO-containing resins (Figure S50) is shifted toward lower temperatures, besides displaying lowered storage moduli. By grouping the materials according to the presence of BDO and DDO and the absence of the linear diol (Figure S51a), it is observed that *E′* shifts toward lower temperatures in the following order: **r3c-3D** > **r1c-3D** > **r2c-3D** (full *T* range), **r3b-3D** > **r1b-3D** > **r2b-3D** (full *T* range), **r1a-3D** > **r2a-3D** (only for *T* < – 30 °C). This behavior can be explained by considering the structures of starting polyols: the methyl and isopropyl groups of geraniol promote considerable self-plasticization, and the cyclohexane ring of limonene provides higher structural rigidity. The methyl and isopropyl groups are present in linalool, but their potential action as self-plasticizers is probably counteracted by OH groups that can form intramolecular and intermolecular H-bonds, resulting in the highest observed structural rigidity.

Destructive mechanical tests were performed using a universal testing machine in the tensile mode to obtain the stiffness and mechanical properties at the break of the material. Stress–strain curves exhibit a trend typical of thermosetting polymeric resins, characterized by the absence of a yield point and, consequently, considerable plastic deformation (Figures S51b and S52). However, most stress–strain curves exhibit a very limited Hookean region (linear region), whereas in some cases, the stress–strain curve is completely nonlinear. The addition of BDO and DDO considerably modifies the tensile properties of analogue resins without BDO or DDO (Figure S51c and Table S6): BDO improves the tensile modulus (+122%) and strength (+53%) of the linalool-based resin (**r2b-3D**), while DDO reasonably increases the deformability of the limonene-based poly(ester-thioether) resin (**r1c-3D**), whose elongation at break increases by +65% without considerable change in its tensile strength.

The high tensile modulus of **r2b-3D**, superior to that of the resin without aliphatic carbon chains (**r2a-3D**), may be because a “short” chain extender favors the previously discussed intramolecular and intermolecular H-bonding interactions, hampering macromolecular motion and forming a stiffer material. Such an effect is also consistent with DMA results; indeed, by extrapolating the *E′* values at 25 °C (Table 4), the same trend is observed for **r2a-3D**, **r2b-3D**, and **r2c-3D**, supporting the hypothesis of maximization of such H-bonding interactions for **r2b-3D**. Even the *E′* values of other formulations at room temperature have trends similar to those of the calculated tensile moduli. Considering the overall mechanical behavior of the tested resins, the most balanced mechanical performances are achieved by using **r1b-3D** and **r3b-3D** resins. Tensile properties of all 3D-printed materials were obtained by averaging replicate measurements, and the error analysis of results shows that the standard deviation of all printed materials is between 5 and 12% of the average value of elastic modulus, between 12 and 30% of the average value of the elongation at break, and between 5 and 22% of the average value of tensile strength. Such variations in the measured properties are consistent with previously reported analogues for materials manufactured via vat photopolymerization, and these variations are small enough to describe good homogeneity of their mechanical properties.^25,26^ While a large set of methacrylated molecules is available in the library of commercially available monomers for vat photopolymerization, allowing the development of materials with various mechanical properties, to date, the scientific literature has proposed only a small selection of sustainable itaconic acid-derived formulations. By comparing the mechanical properties of the obtained materials with those of previously reported polyesters^25^ and poly(ester-amides)^26^ of itaconic acid, it can be seen that poly(ester-thioether)s can be used to obtain materials with higher elastic moduli (100–300 MPa compared to <100 MPa), lower elongations at break (4–12% compared to 18–38%), and generally higher tensile strengths (5–10 MPa compared to 4–5 MPa). However, a detailed rational comparison is not straightforward because such photocurable formulations are composed of a numerous different comonomers and cross-linkers and are manufactured using different 3D printing techniques, all factors that greatly affect the mechanical properties of final thermosets.

## Conclusions

This study demonstrated the feasibility of preparing poly(ester-thioether)s via the tin-catalyzed poly transesterification of the dimethyl ester of itaconic acid, a green photocurable building block, with terpene polyols obtained via the thiol–ene addition of 2-mercaptoethanol to naturally occurring terpenes and, in some cases, with the addition of linear aliphatic α,ω-diols as comonomers. Such poly(ester-thioether)s were efficiently formulated using an itaconic acid–based reactive diluent and an appropriate photoinitiating system to produce photocurable resins, which were used for the vat photopolymerization of biobased materials with high printing resolution. The photocurable resins underwent thorough physical and chemical characterizations. No acrylic or methacrylic acid derivative was used to prepare the resins, which were characterized by the highest overall biobased contents reported to date for applications in vat photopolymerization. The detailed thermal, thermomechanical, and mechanical analyses of 3D-printed materials allowed the correlation of the macromolecular features of poly(ester-thioether)s with the properties of the 3D-printed material.

- All 3D-printed materials exhibit two glass-transition temperatures, whose positions are a function of the poly(ester-thioether) chains, because they strongly depend on the type of diol or polyol used during polymer preparation, suggesting a sort of phase compartmentalization upon network formation.
- The 3D-printed thermosets exhibit good thermal stability, with no appreciable mass loss up to 150–160 °C and are characterized by a main degradation step at >300 °C.
- DMA of 3D-printed materials shows that the molecular structure of the incorporated terpene polyols affects the thermomechanical properties of the material. The cyclohexane ring in limonene-based thioether polyol **1** provides structural rigidity (and therefore higher *E′* values), whereas the pendant methyl and isopropyl groups in geraniol polyol **3** exhibit self-plasticizing properties, allowing higher macromolecular mobility related to lower *E′* values. In thioether polyol **2**, the H-bonding ability of the free tertiary alcoholic moiety counteracts the plasticizing properties of the pending functionalities, leading to the lowest observed chain mobility.
- Tensile testing was used to correlate the effect of different copolymerized linear aliphatic α,ω-diols on the mechanical properties of the 3D-printed material, revealing that the effect of the diol chain length relates with the molecular structure of the terpene polyol employed.

The reported results show the promising applicability of itaconic acid as a replacement for toxic and fossil fuel-based acrylates and methacrylates, revealing the high versatility and promising thermal and mechanical properties of this type of formulation and suggesting a potential great benefit in evaluating the biobased content of photopolymerizable resins to solve issues related to environmental and toxicity concerns for numerous applications.

## Supporting Information Available

NMR, ATR-FTIR, and ESI-MS analyses of thioether-polyols; NMR and rheology of poly(ester-thioether)s; ATR-FTIR analysis of photocurable formulations before and after 3D printing; solvent stability of 3D-printed materials; thermogravimetric analyses; DSC thermograms; DMA analyses; tensile stress–strain curves (DOCX)
