# Supplementary material for: (Meth)acrylate-Free Three-Dimensional Printing of Bio-Derived Photocurable Resins with Terpene- and Itaconic Acid-Derived Poly(ester-thioether)s
Source: ACS Sustain Chem Eng. 2023 Nov 28;11(49):17285–98. doi: 10.1021/acssuschemeng.3c04576 (PMC10716902; doi:10.1021/acssuschemeng.3c04576)
Supplement: Supplementary file 1 — sc3c04576_si_001.docx [file sc3c04576_si_001.docx]

(Meth)acrylate-free 3D printing of bio-derived photocurable resins with terpene- and itaconic acid-derived poly(ester-thioether)s

Mirko Maturi ^†^, Chiara Spanu ^†^, Emanuele Maccaferri ^†,‡^, Erica Locatelli ^†^, Tiziana Benelli ^†,‡^, Laura Mazzocchetti ^†,‡^, Letizia Sambri ^†^, Loris Giorgini ^†,‡^, and Mauro Comes Franchini ^†^*

^†^ Department of Industrial Chemistry “Toso Montanari”, University of Bologna, Viale Risorgimento 4, 40136 Bologna, Italy.

^‡^ Interdepartmental Center for Industrial Research on Advanced Applications in Mechanical Engineering and Materials Technology, CIRI‑MAM, University of Bologna, Viale Risorgimento 2, 40136 Bologna, Italy.

**SUPPORTING INFORMATION**

This Supporting Information file contains 52 figures and 14 tables in 61 pages.

**TABLE OF CONTENTS**

**Figure S1:** ESI-MS spectrum of thioether-polyol **1**……………………………………………………Page S3

**Figure S2:** ATR-FTIR of thioether-polyol **1**……………………...……………………………………Page S3

**Figure S3:** ESI-MS spectrum of thioether-polyol **2**……………………………………………………Page S4

**Figure S4:** ATR-FTIR of thioether-polyol **2**……………………………………………………...……Page S4

**Figure S5:** ESI-MS spectrum of thioether-polyol **3**……………………………………………………Page S5

**Figure S6:** ATR-FTIR of thioether-polyol **3**……………………………………………………...……Page S5

**Figure S7:** Structure of thioether-polyol **1** and its diastereomers………………………………...……Page S6

**Figure S8 – S10:** NMR characterization of thioether-polyol **1**………………………...…………..Page S7-S9

**Table S1:** NMR peak assignments for thioether-polyol **1**…………………………………….………Page S10

**Figure S11:** Structure of thioether-polyol **2** and its diastereomers…………...………………………Page S11

**Figure S12 – S14:** NMR characterization of thioether-polyol **2**……………………...…………Page S12-S14

**Table S2:** NMR peak assignments for thioether-polyol **2**…………………………………….………Page S15

**Figure S15:** Structure of thioether-polyol **3** and its diastereomers………………………………...…Page S16

**Figure S16 – S18:** NMR characterization of thioether-polyol **3**……………………...…………Page S17-S19

**Table S3:** NMR peak assignments for thioether-polyol **3**…………………………………….………Page S20

**Figure S19:** Mechanism of DBTO-catalyzed transesterification……………………….…….………Page S21

**Figure S20 – S21:** NMR analysis of polymer **1a**……………………….…….…………………Page S22-S23

**Figure S22 – S23:** NMR analysis of polymer **1b**……………………….…….…………………Page S24-S25

**Figure S24 – S25:** NMR analysis of polymer **1c**……………………….…….…………………Page S26-S27

**Figure S26 – S27:** NMR analysis of polymer **2a**……………………….…….…………………Page S28-S29

**Figure S28 – S29:** NMR analysis of polymer **2b**……………………….…….…………………Page S30-S31

**Figure S30 – S31:** NMR analysis of polymer **2c**……………………….…….…………………Page S32-S33

**Figure S32 – S33:** NMR analysis of polymer **3b**……………………….…….…………………Page S34-S35

**Figure S34 – S35:** NMR analysis of polymer **3c**……………………….…….…………………Page S36-S37

**Figure S36 –** Viscosity of the poly(ester-thioether)s as a function of temperature..….………………Page S38

**Figure S37 –** ^1^H-NMR spectrum of I_2_B_1_……………………….…….……………….………………Page S39

**Figure S38 –** Viscosity of I_2_B_1_ as a function of temperature……………………….…….………..…Page S39

**Figure S39 –** Viscosity of the formulated resins as a function of temperature………………….……Page S40

Quantification of total biobased carbon content……………………….…….……………...……Page S41-S46

**Table S4**: Calculated parameters for the evaluation of the biobased content of the formulations...….Page S46

**Figure S40 – S41:** Printed object arrangement and slicing……………………….……………..……Page S47

**Table S5**: Quantitative comparison of 3D printing accuracy……………………….…….……..……Page S48

**Figure S42 – S44:** ATR-FTIR of photocurable formulations before and after 3D printing..……Page S49-S51

**Figure S45 – S47:** Evaluation of solvent compatibility of 3D printed resins……………………Page S52-S54

**Figure S48**: Thermogravimetric analysis of 3D printed materials………………….….………..……Page S55

**Figure S49**: Differential scanning calorimetry thermograms of 3D printed materials…………….…Page S56

**Figure S50**: DMA analysis of 3D printed materials……………………………...…….………..……Page S57

**Figure S51**: Thermomechanical and mechanical characterization of 3D printed materials…..…...…Page S58

**Figure S52**: Tensile stress-strain curves of 3D printed materials…………………….………..…...…Page S59

**Table S6**: Tensile and hardness properties of 3D printed materials………………….………..…...…Page S60


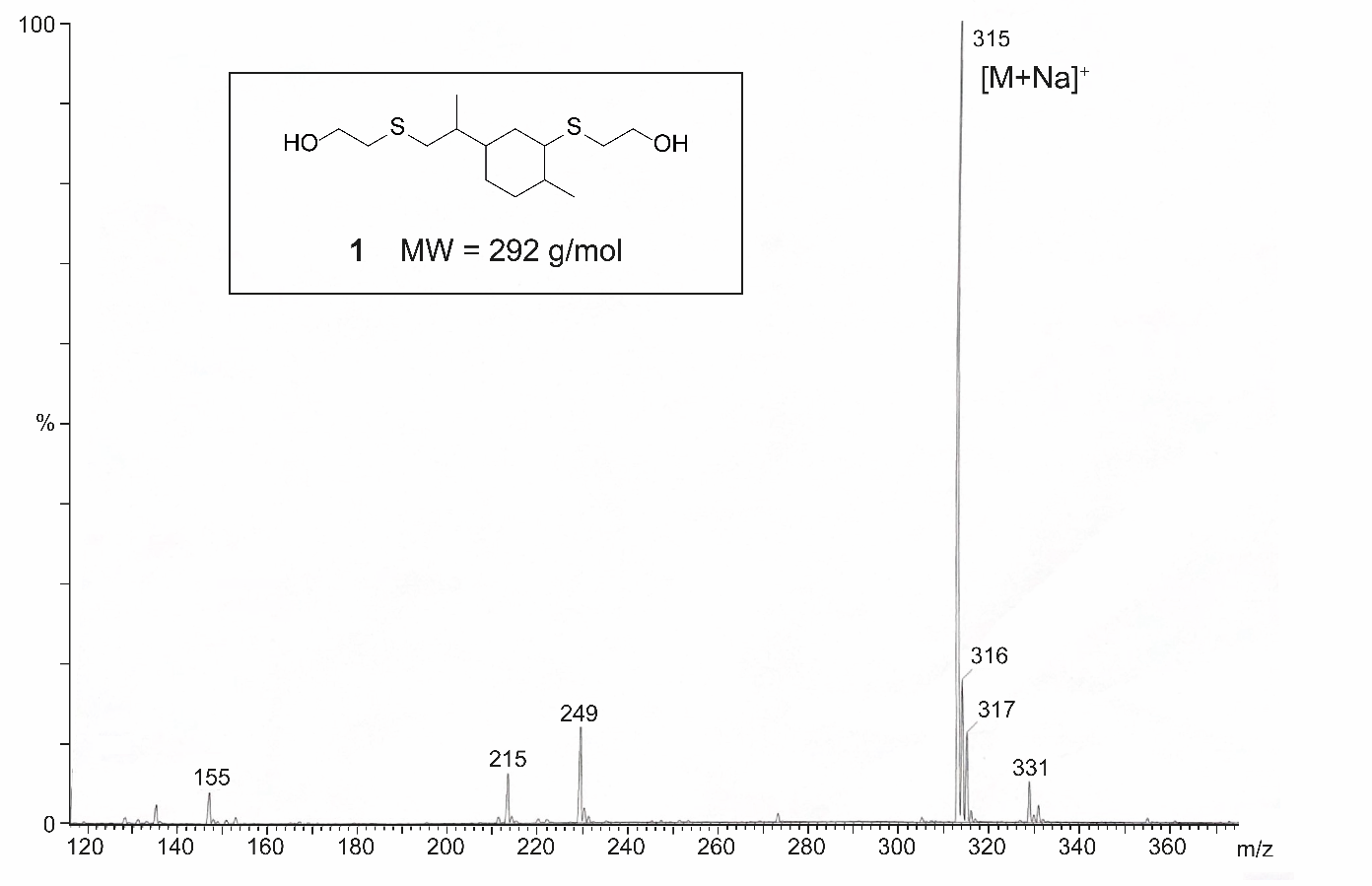


**Figure S1.** ESI-MS spectrum of thioether-polyol **1**.


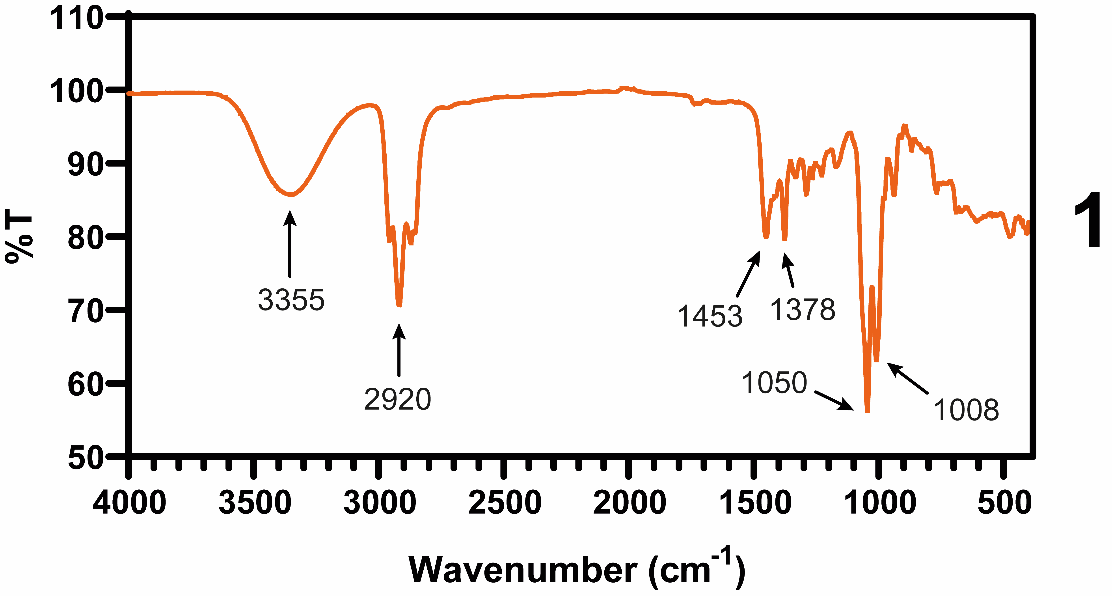


**Figure S2.** ATR-FTIR spectrum of thioether-polyol **1**.


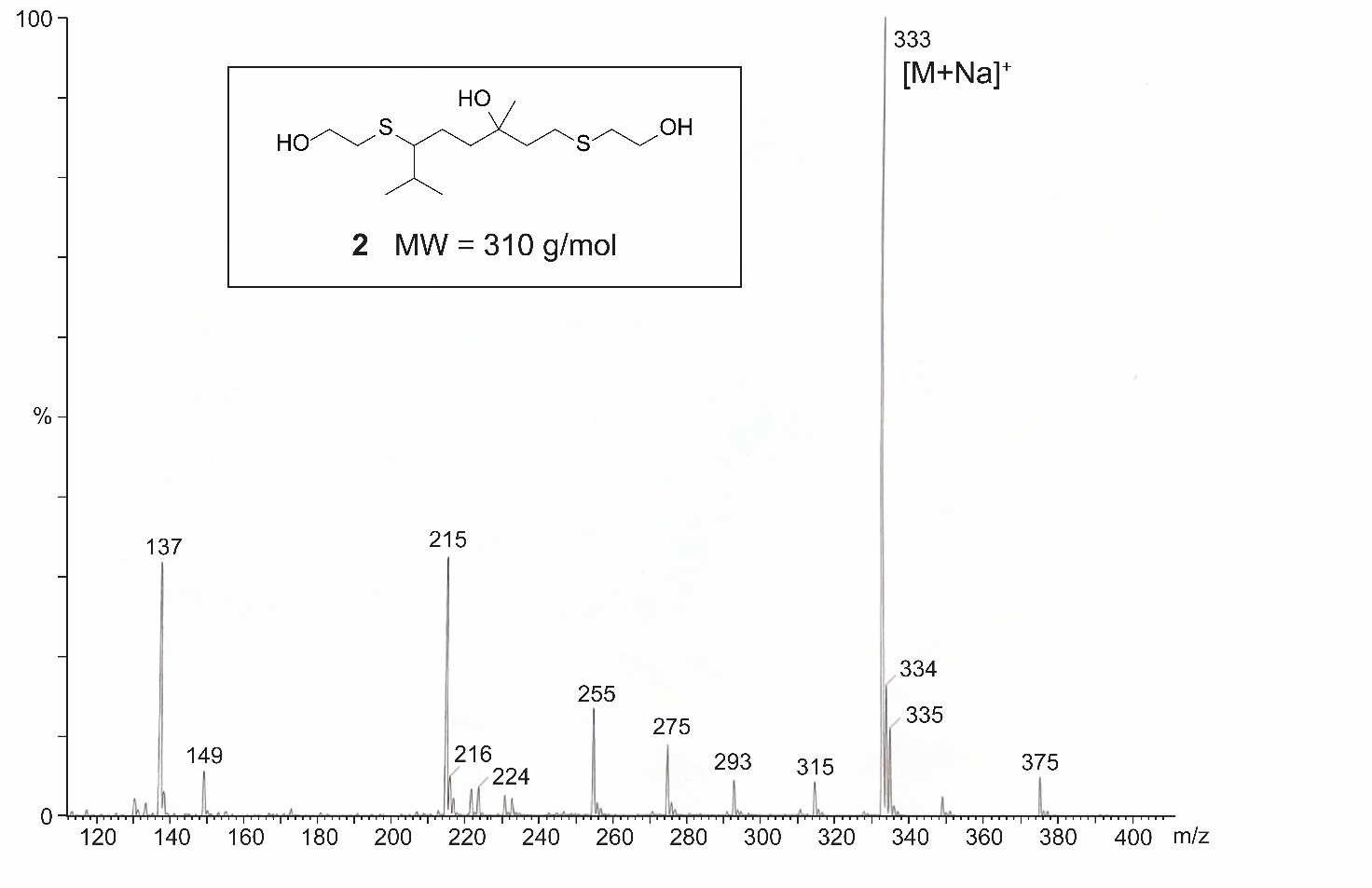


**Figure S3.** ESI-MS spectrum of thioether-polyol **2**.


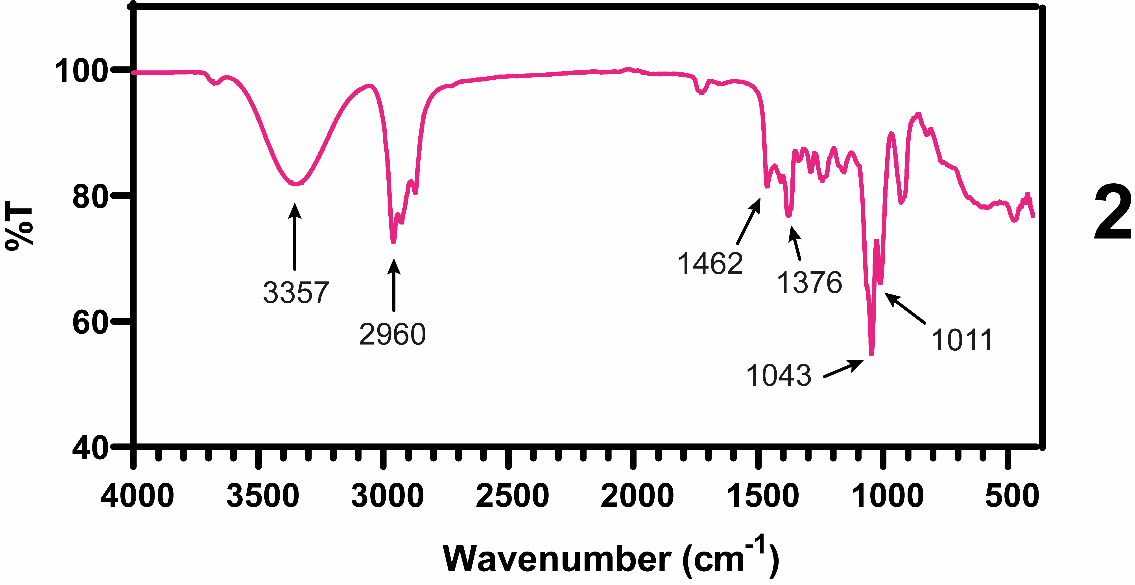


**Figure S4.** ATR-FTIR spectrum of thioether-polyol **2**. **
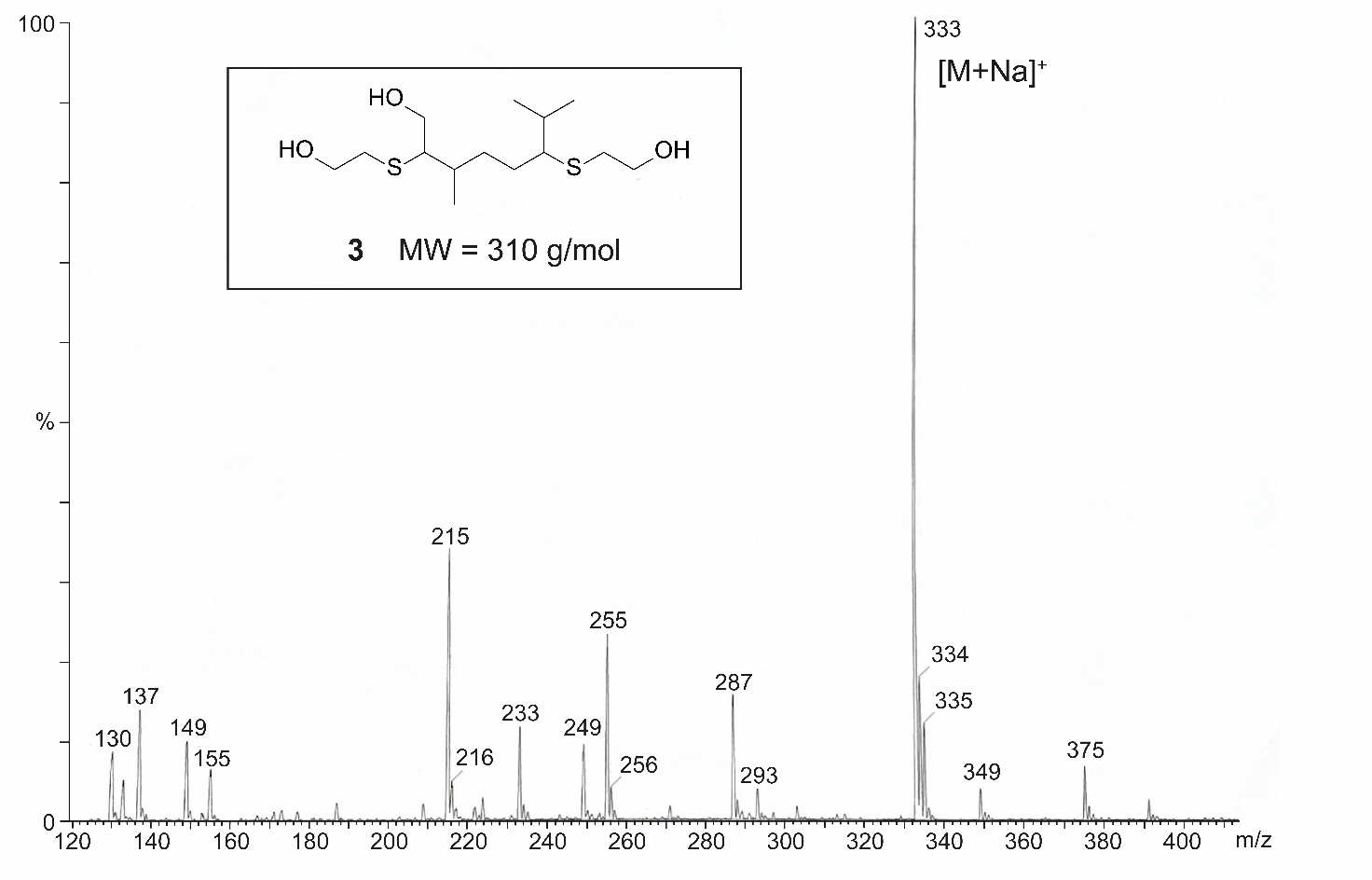
**

**Figure S5.** ESI-MS spectrum of thioether-polyol **3**.


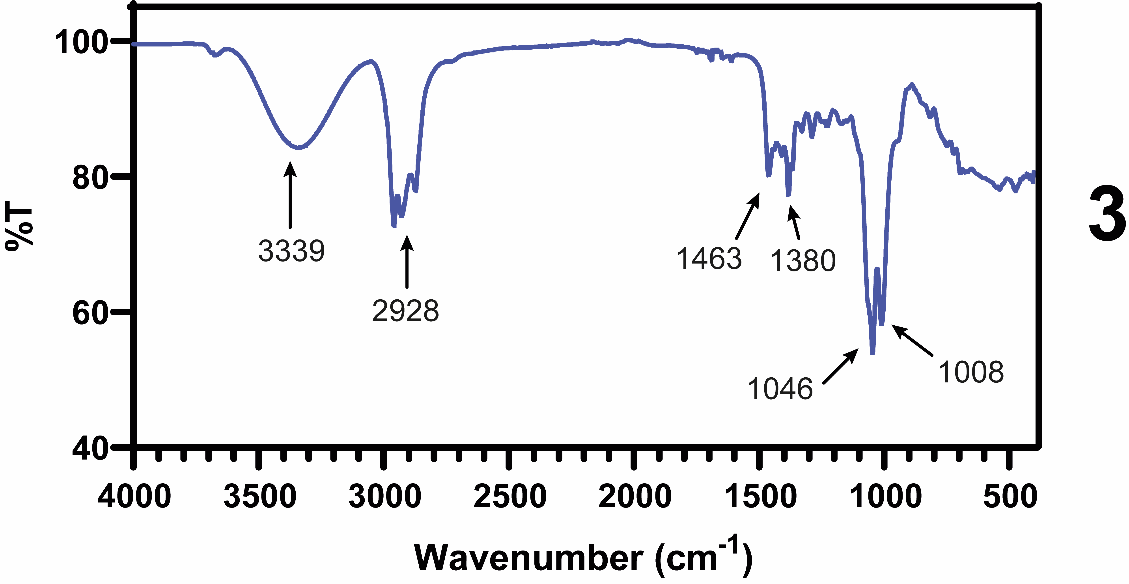


**Figure S6.** ATR-FTIR spectrum of thioether-polyol **3**.

**Figure S7.** Structure of thioether-polyol **1** and all its diastereomers. The *trans*-diaxial products are expected to be the most abundant in the product mixture.

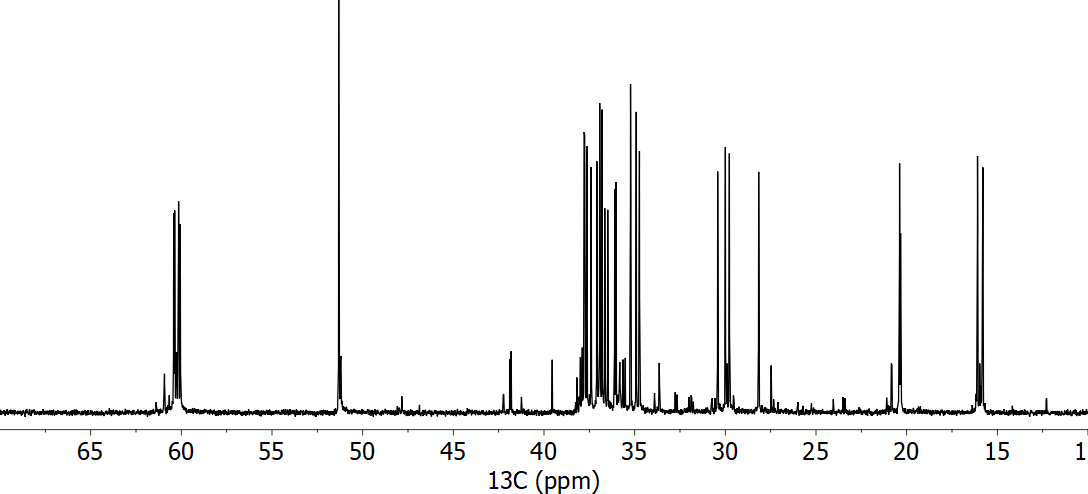


**Figure S8.** ^1^H- (600 MHz, CDCl_3_, top) and ^13^C- (100 MHz, CDCl_3_, bottom) NMR spectra of thioether-polyol **1**.


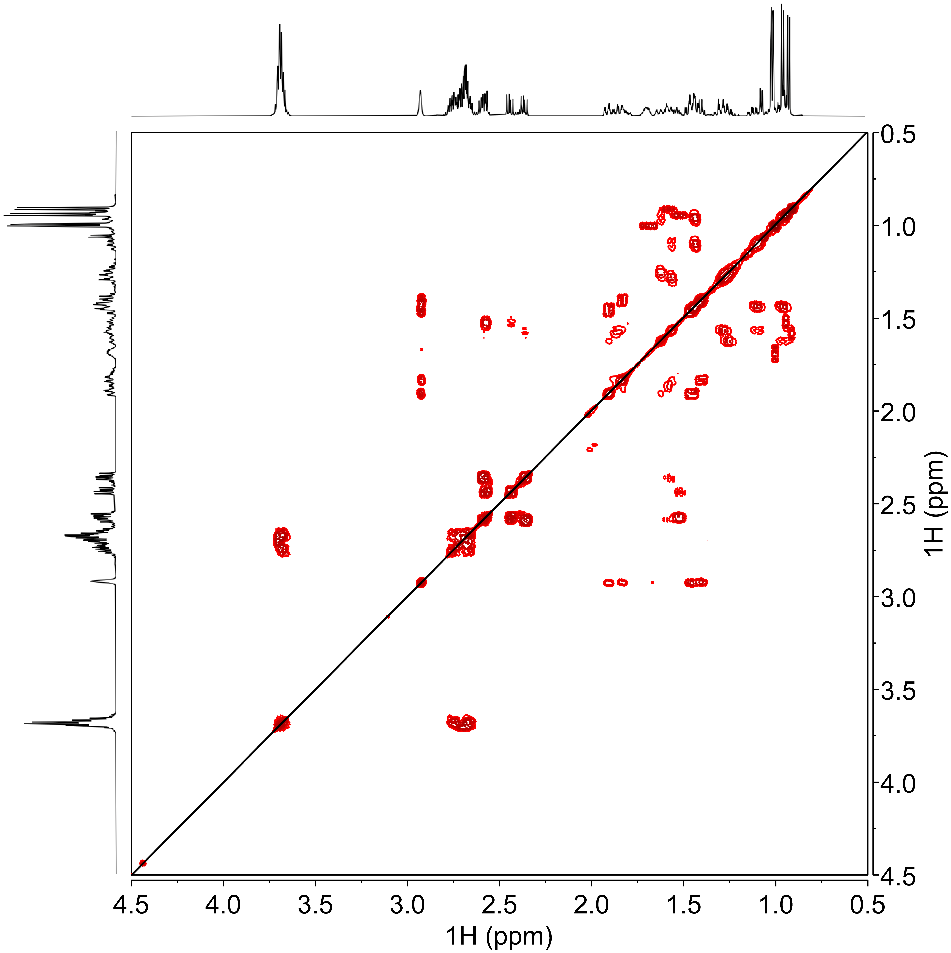

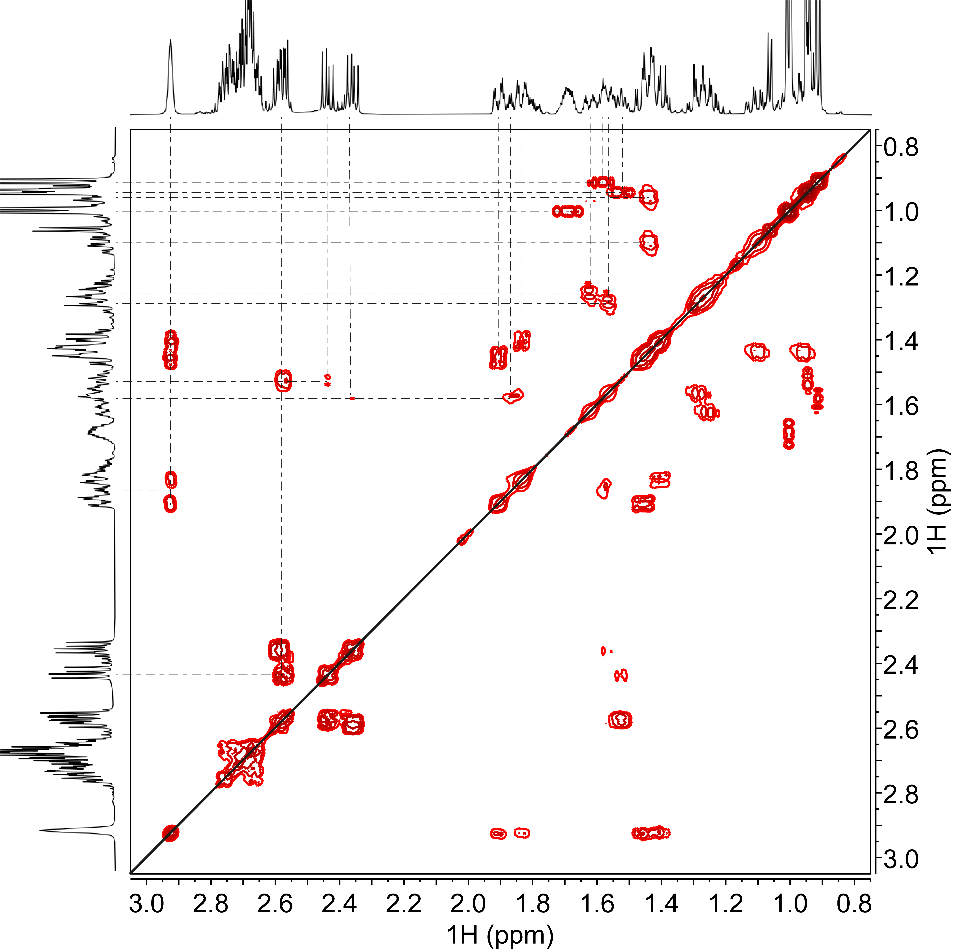


**Figure S9.** ^1^H-^1^H COSY (600 MHz, CDCl_3_) NMR analysis of thioether-polyol **1**. Full spectrum (top) and expansion (bottom).


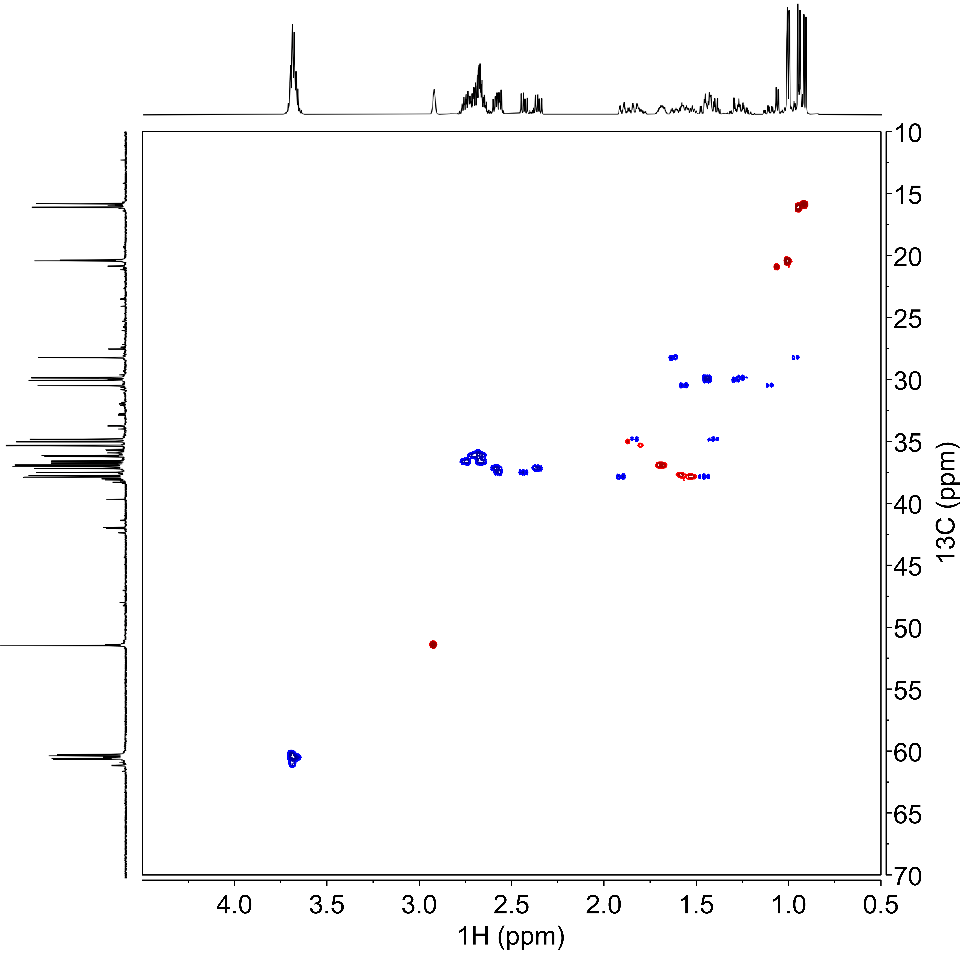


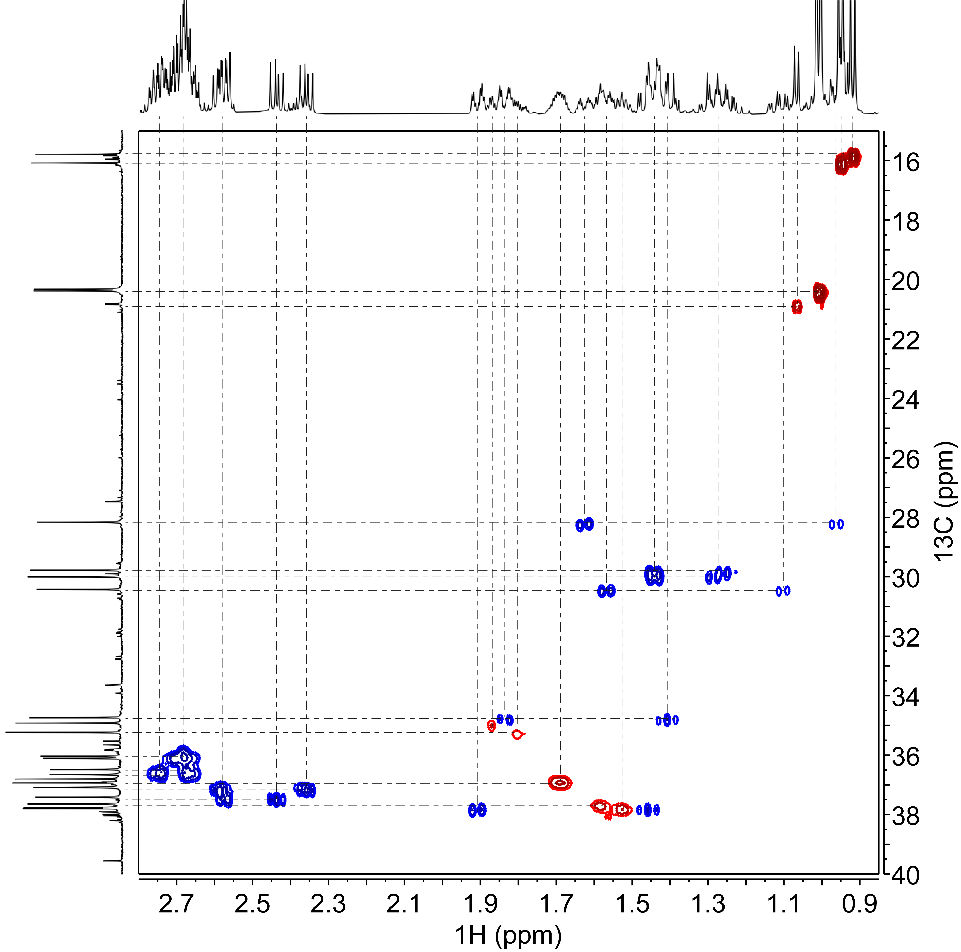


**Figure S10.** ^1^H-^13^C HSQC (600 MHz, CDCl_3_) NMR analysis of thioether-polyol **1**. Full spectrum (top) and expansion (bottom). Positive peaks are coloured in red and are related to CH and CH_3_ groups, while negative peaks are coloured in blue and are related to CH_2_ groups.


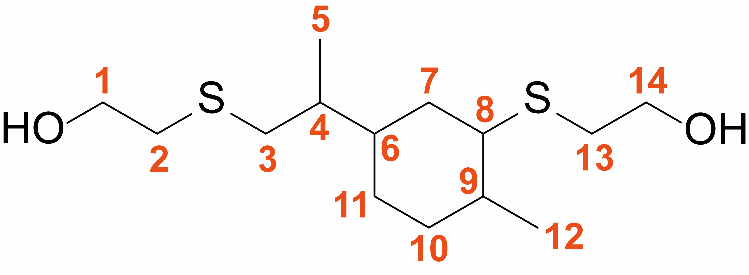


|  | **DIASTEROMER 1** | | **DIASTEROMER 2** | |
| --- | --- | --- | --- | --- |
|  | **δ ^1^H (ppm)** | **δ ^13^C (ppm)** | **δ ^1^H (ppm)** | **δ ^13^C (ppm)** |
| **1** | 3.68 | 59.8 – 60.6 |  |  |
| **2** | 2.62 – 2.80 | 35.6 – 36.8 |  |  |
| **3a** | 2.43 | 37.5 | 2.36 | 37.2 |
| **3b** | 2.57 |  | 2.58 |  |
| **4^1^** | 1.58 | 37.7 | 1.53 | 37.9 |
| **5^1^** | 0.91 | 15.9 | 0.95 | 16.2 |
| **6^1^** | 1.80 | 35.3 | 1.87 | 35.0 |
| **7a** | 1.40 | 34.8 | 1.46 | 37.9 |
| **7b** | 1.84 |  | 1.91 |  |
| **8** | 2.92 | 51.4 |  |  |
| **9** | 1.69 | 36.9 |  |  |
| **10a** | 1.27 | 29.9 |  |  |
| **10b** | 1.44 |  |  |  |
| **11a** | 0.96 | 28.3 | 1.10 | 30.5 |
| **11b** | 1.62 |  | 1.57 |  |
| **12** | 1.00 | 20.5 |  |  |
| **13** | 2.62 – 2.80 | 35.6 – 36.8 |  |  |
| **14** | 3.68 | 59.8 – 60.6 |  |  |

**Table S1**. Full NMR peak assignments for thioether-polyol **1**. All signals related to protons close to the asymmetric carbon atoms in positions 4 and 6 are split into two sets of peaks, assigned to different diasteromers.

**
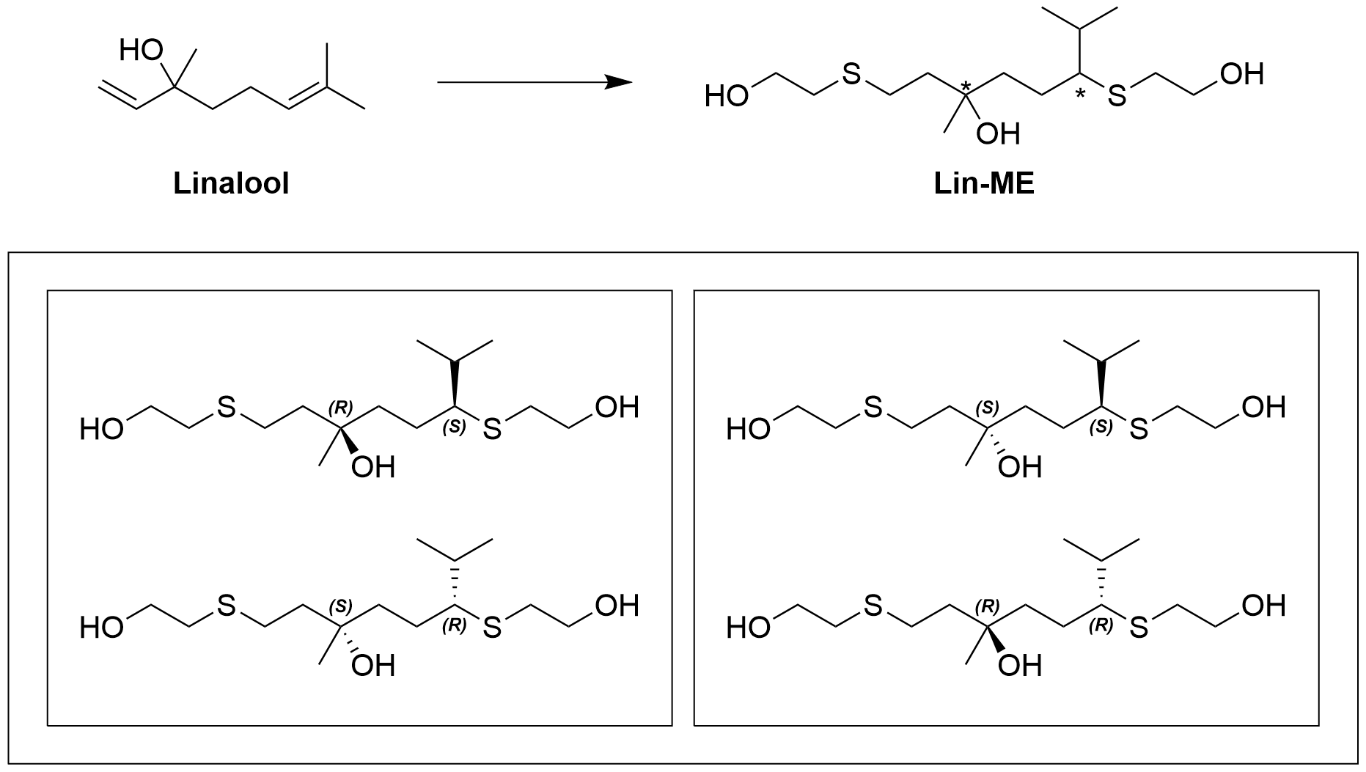
**

**Figure S11.** Structure of thioether-polyol **2** and its diastereomers.

**
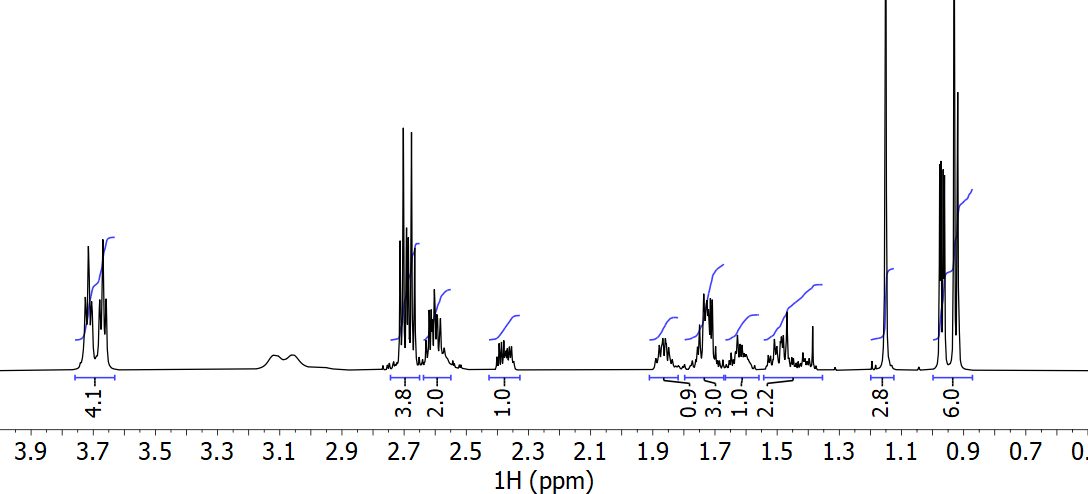
**


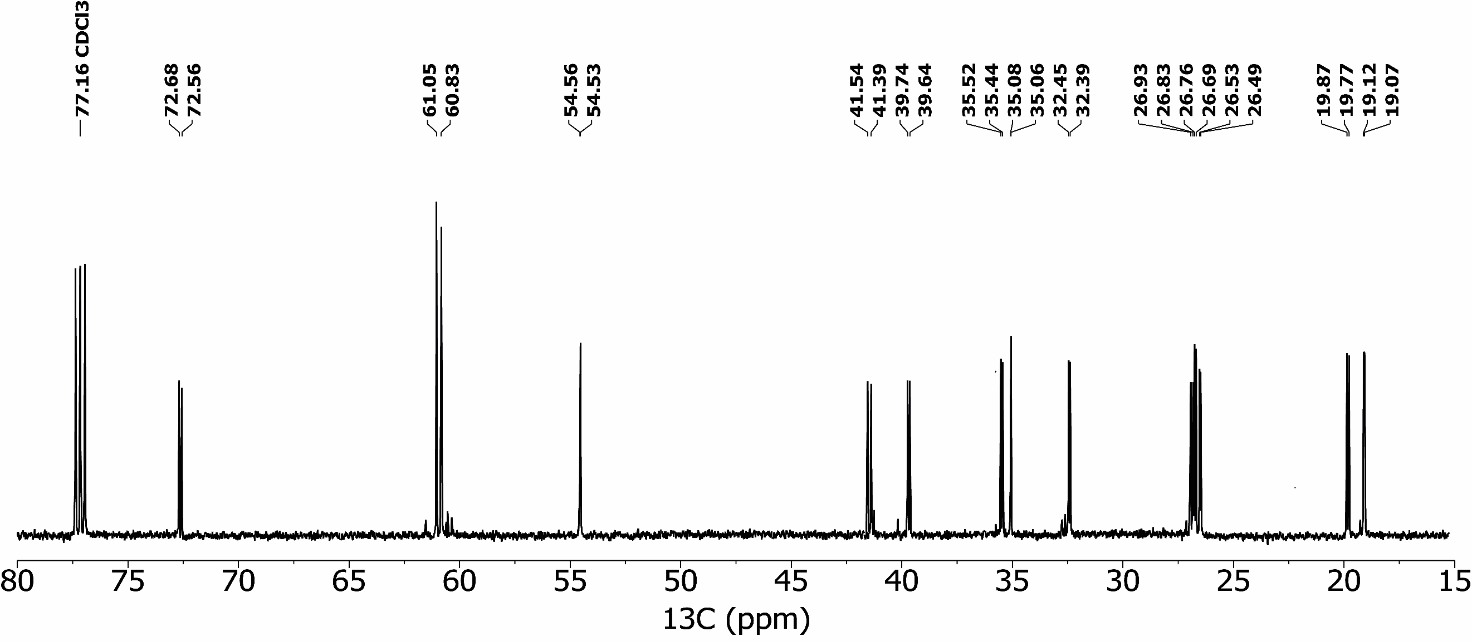


**Figure S12.** ^1^H- (600 MHz, CDCl_3_, top) and ^13^C- (100 MHz, CDCl_3_, bottom) NMR spectra of thioether-polyol **2**.


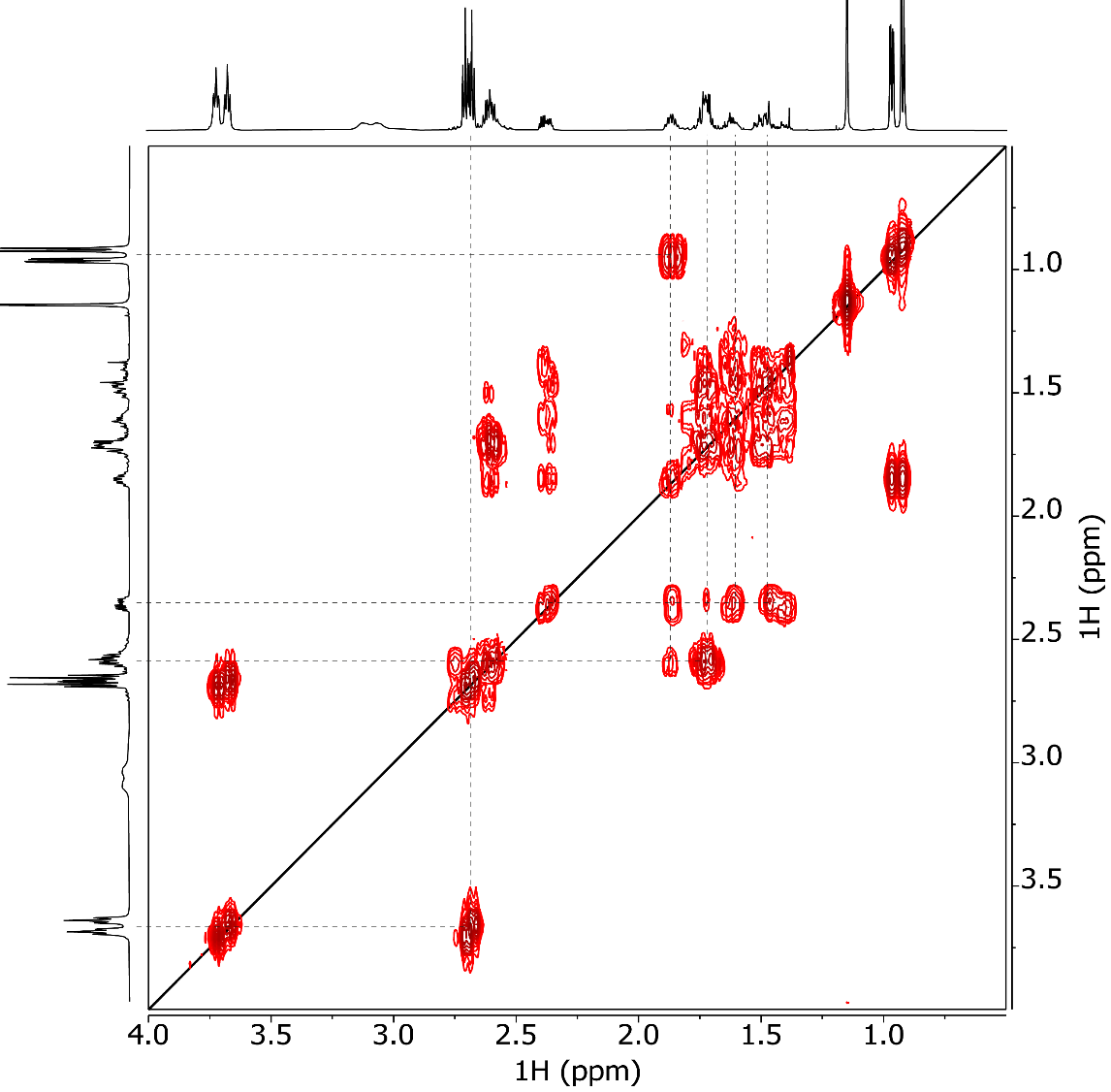


**Figure S13.** ^1^H-^1^H COSY (600 MHz, CDCl_3_) NMR analysis of thioether-polyol **2**.


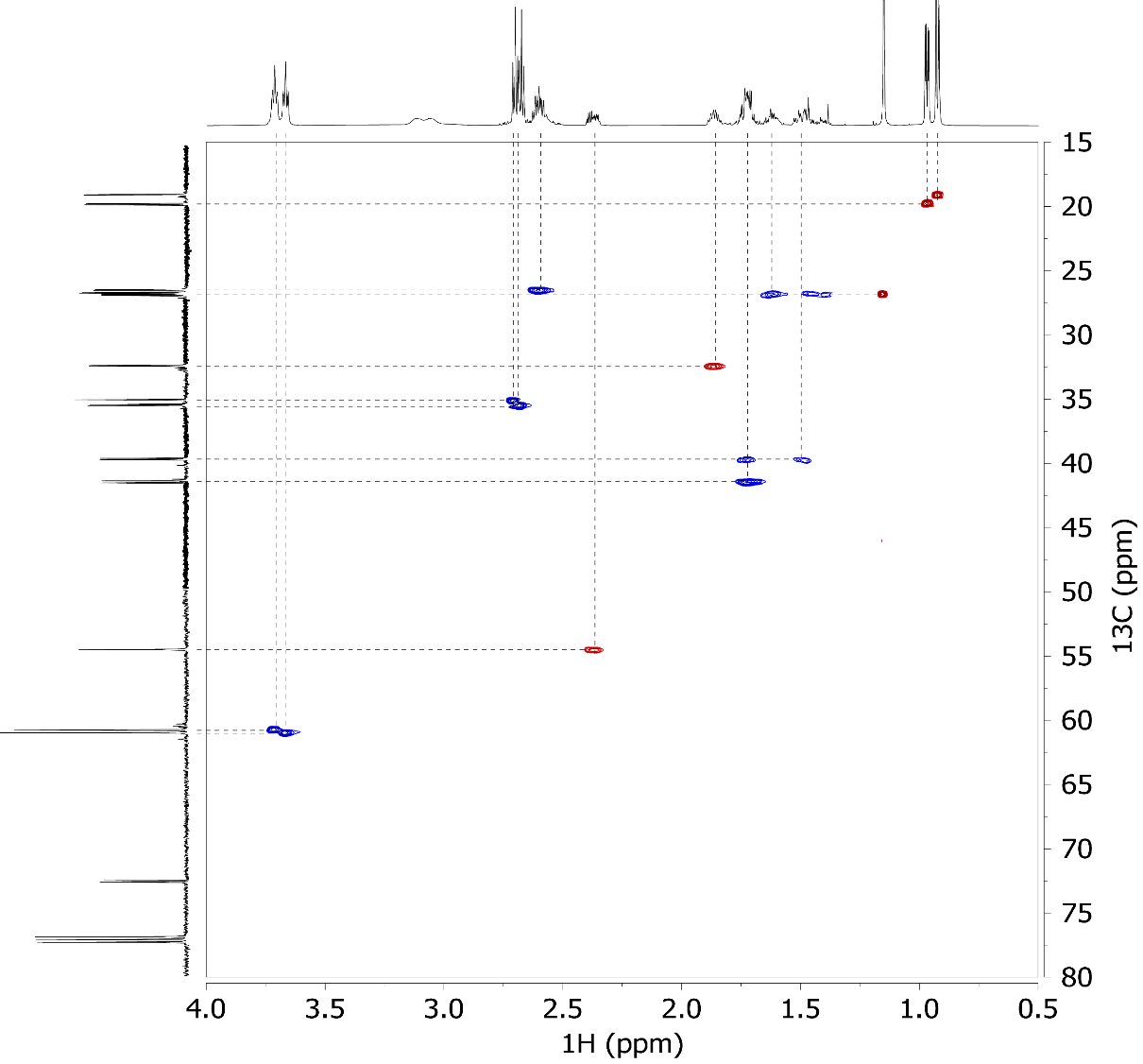


**Figure S14.** ^1^H-^13^C HSQC (600 MHz, CDCl_3_) NMR analysis of thioether-polyol **2**. Positive peaks are coloured in red and are related to CH and CH_3_ groups, while negative peaks are coloured in blue and are related to CH_2_ groups.


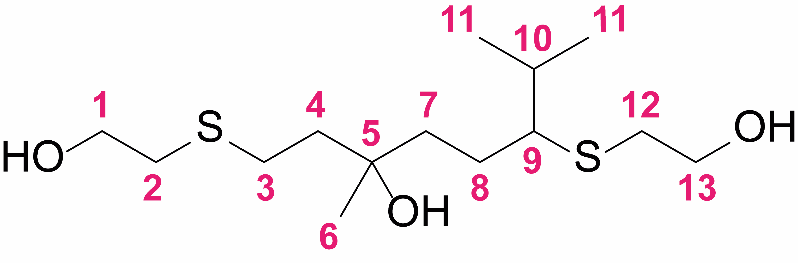


|  | **δ ^1^H (ppm)** | **δ ^13^C (ppm)** |
| --- | --- | --- |
| **1** | 3.72 and 3.67 | 60.83 and 61.05 |
| **2** | 2.67 and 2.70 | 35.06, 35.08, 35.44 and 35.52 |
| **3** | 2.60 | 26.49 and 26.53 |
| **4** | 1.72 | 41.39 and 41.54 |
| **5** | / | 72.56 and 72.78 |
| **6** | 1.15 | 26.83 and 26.93 |
| **7a** | 1.48 | 39.64 and 39.74 |
| **7b** | 1.74 |  |
| **8a** | 1.38 | 26.76 and 26.69 |
| **8b** | 1.61 |  |
| **9** | 2.37 | 54.53 and 54.56 |
| **10** | 1.86 | 32.39 and 32.45 |
| **11** | 0.93 and 0.97 | 19.0, 19.1, 19.8 and 19.9 |
| **12** | 2.67 and 2.70 | 35.06, 35.08, 35.44 and 35.52 |
| **13** | 3.72 and 3.67 | 60.83 and 61.05 |

**Table S2**. Full NMR peak assignments for thioether-polyol **2**. All signals are doubled due to the presence of two diasteromers.

**
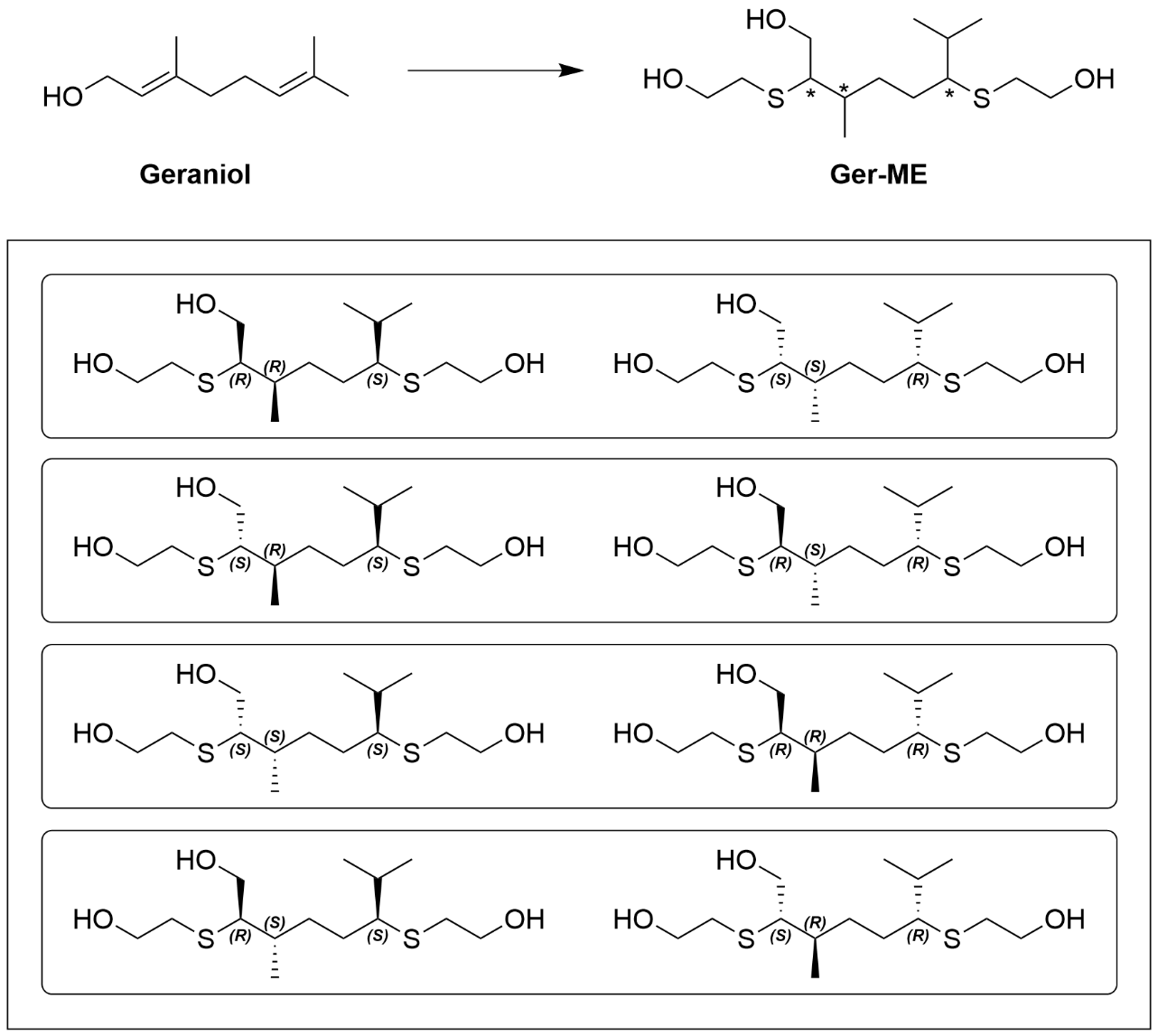
**

**Figure S15.** Structure of thioether-polyol **3** and its diastereomers.

**
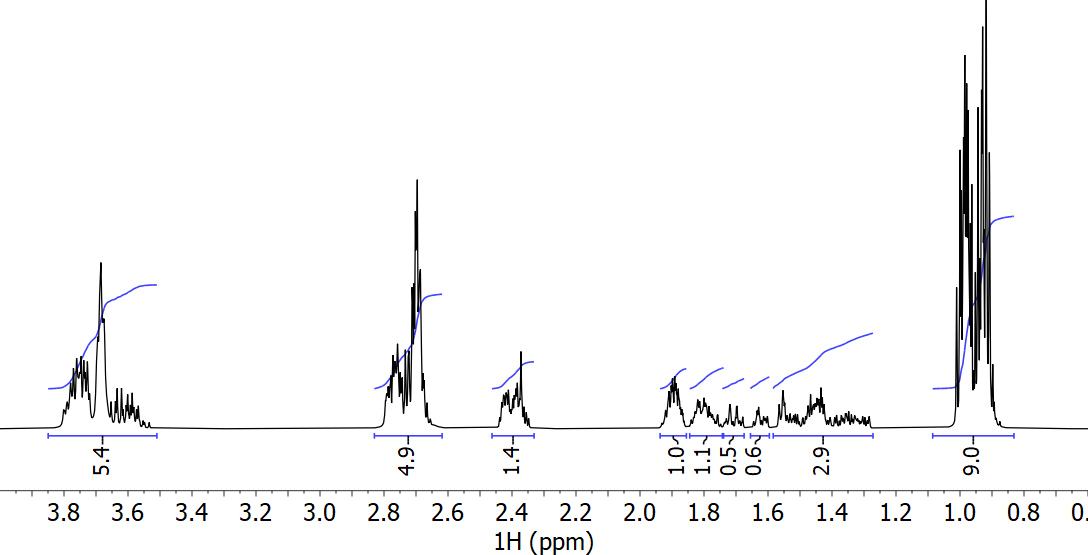
**

**
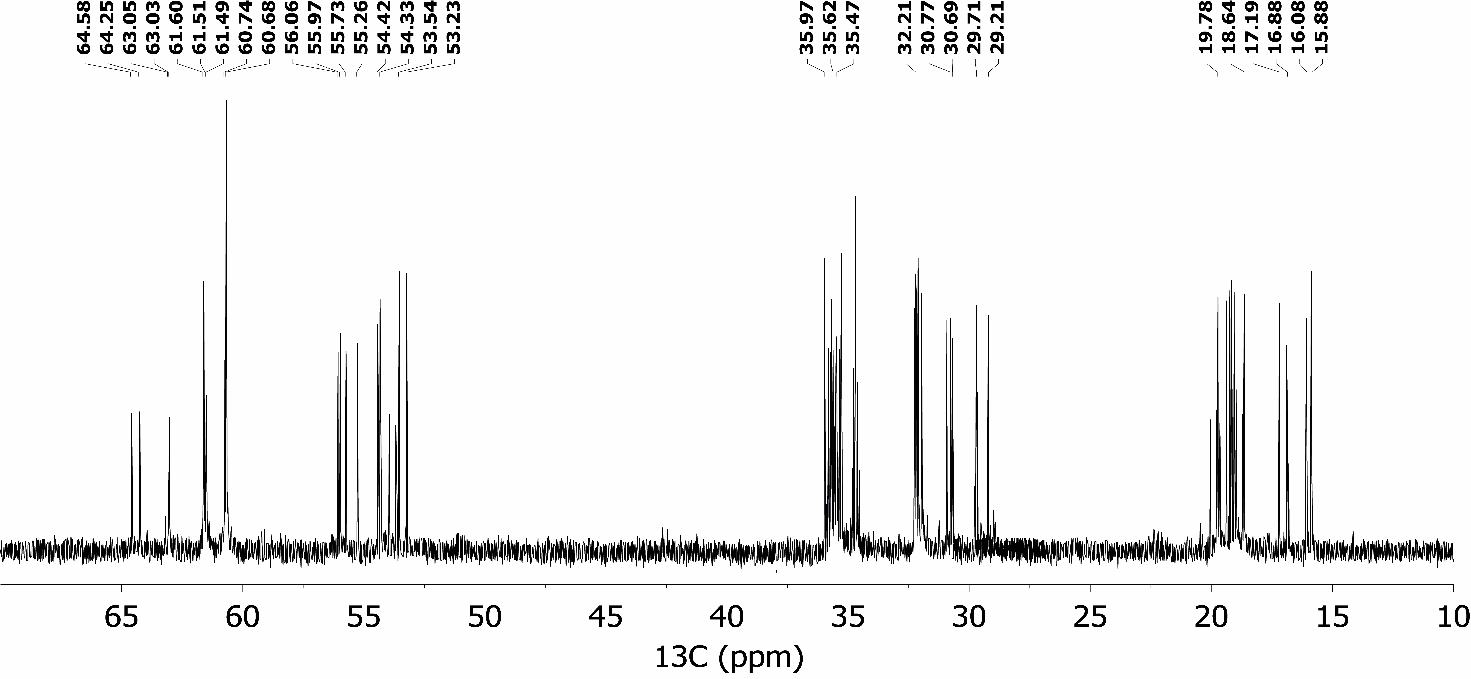
**

**Figure S16.** ^1^H- (600 MHz, CDCl_3_, top) and ^13^C- (100 MHz, CDCl_3_, bottom) NMR spectra of thioether-polyol **3**.


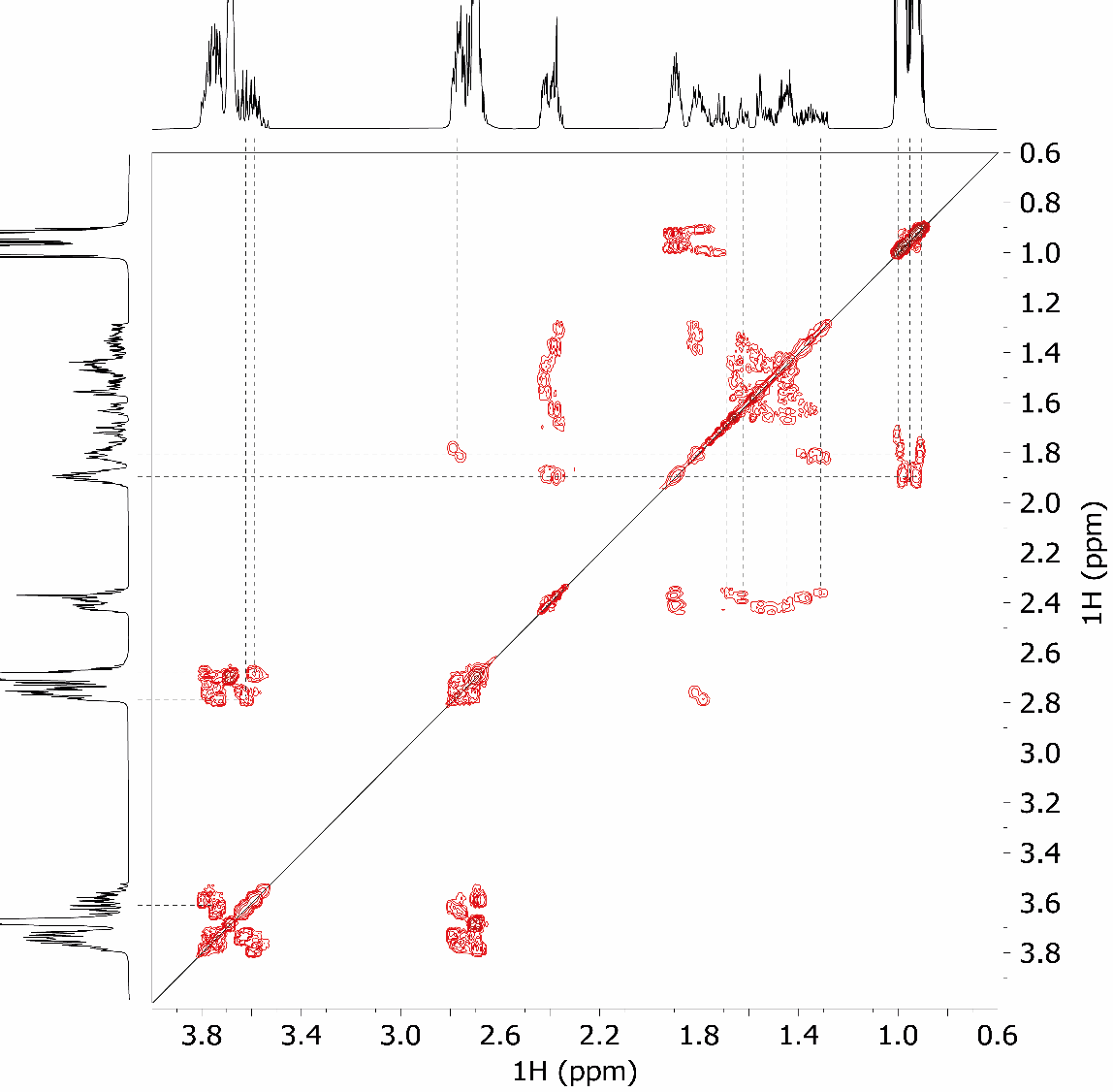


**Figure S17.** ^1^H-^1i^H COSY (600 MHz, CDCl_3_) NMR analysis of thioether-polyol **3**.


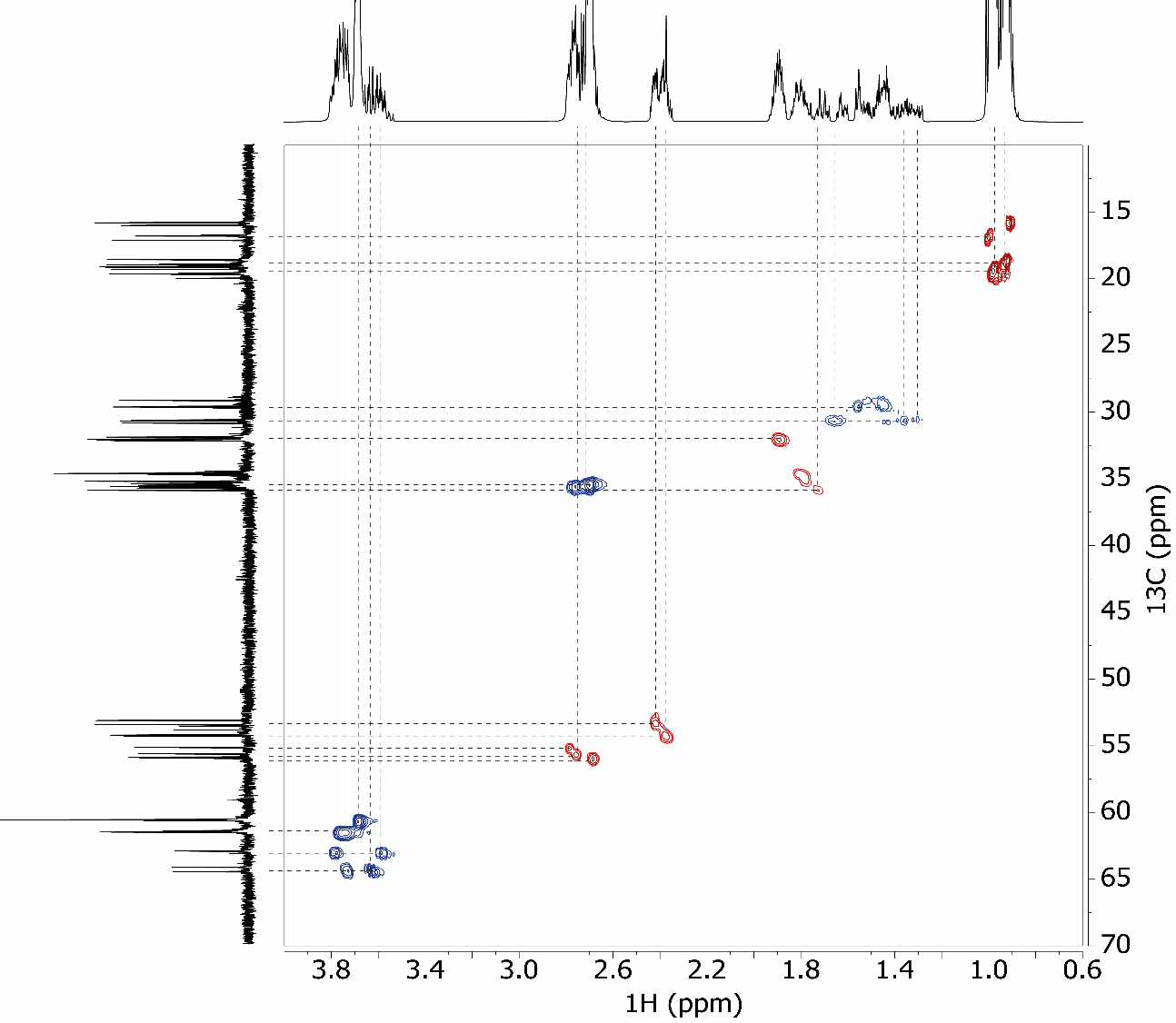


**Figure S18.** ^1^H-^13^C HSQC (600 MHz, CDCl_3_) NMR analysis of thioether-polyol **3**. Positive peaks are coloured in red and are related to CH and CH_3_ groups, while negative peaks are coloured in blue and are related to CH_2_ groups.


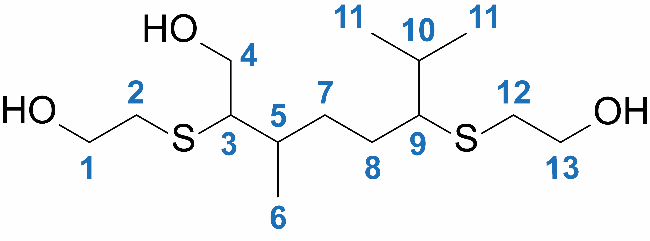


|  | **δ ^1^H (ppm)** | **δ ^13^C (ppm)** |
| --- | --- | --- |
| **1** | 3.68 and 3.75 | 60.67 and 61.55 |
| **2** | 2.70 and 2.76 | 35.45 and 35.60 |
| **3** | 2.69, 2.75 and 2.78 | 55.26, 55.73, 55.97 and 56.06 |
| **4** | 3.58, 3.63, 3.73 and 3.78 | 63.02, 63.05, 64.25 and 64.58 |
| **5** | 1.78 and 1.81 | 36.70 |
| **6** | 0.90, 0.92, 0.98 and 1.00 | 15.87, 16.08, 16.89 and 17.18 |
| **7** | 1.35 and 1.66 | 30.70 and 30.78 |
| **8** | 1.44 and 1.56 | 29.19 and 29.68 |
| **9** | 2.38 and 2.42 | 53.24, 53.53, 54.34 and 54.43 |
| **10** | 1.89 | 32.11 |
| **11** | 0.93 and 0.98 | 18.65 to 19.75 |
| **12** | 2.70 and 2.76 | 35.45 and 35.60 |
| **13** | 3.68 and 3.75 | 60.67 and 61.55 |

**Table S3**. Full NMR peak assignments for thioether-polyol **3**. All signals are split in four due to the presence of four diasteromers.


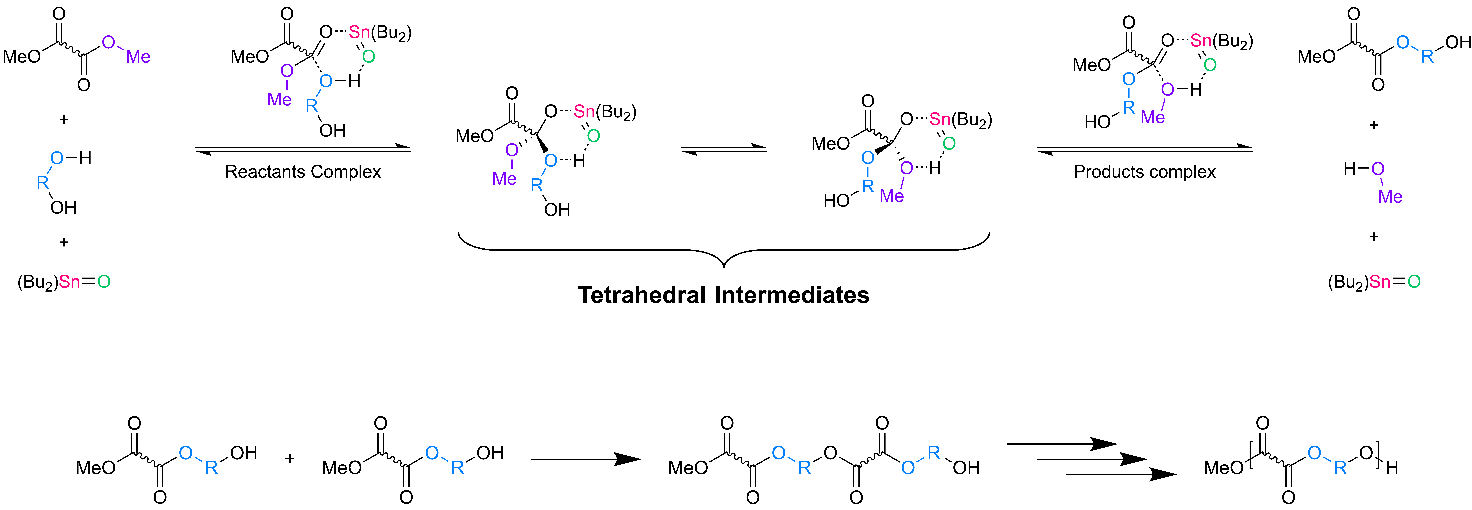


**Figure S19.** Mechanism of DBTO-catalysed transesterification, as proposed by Bhusal et al. [REF]


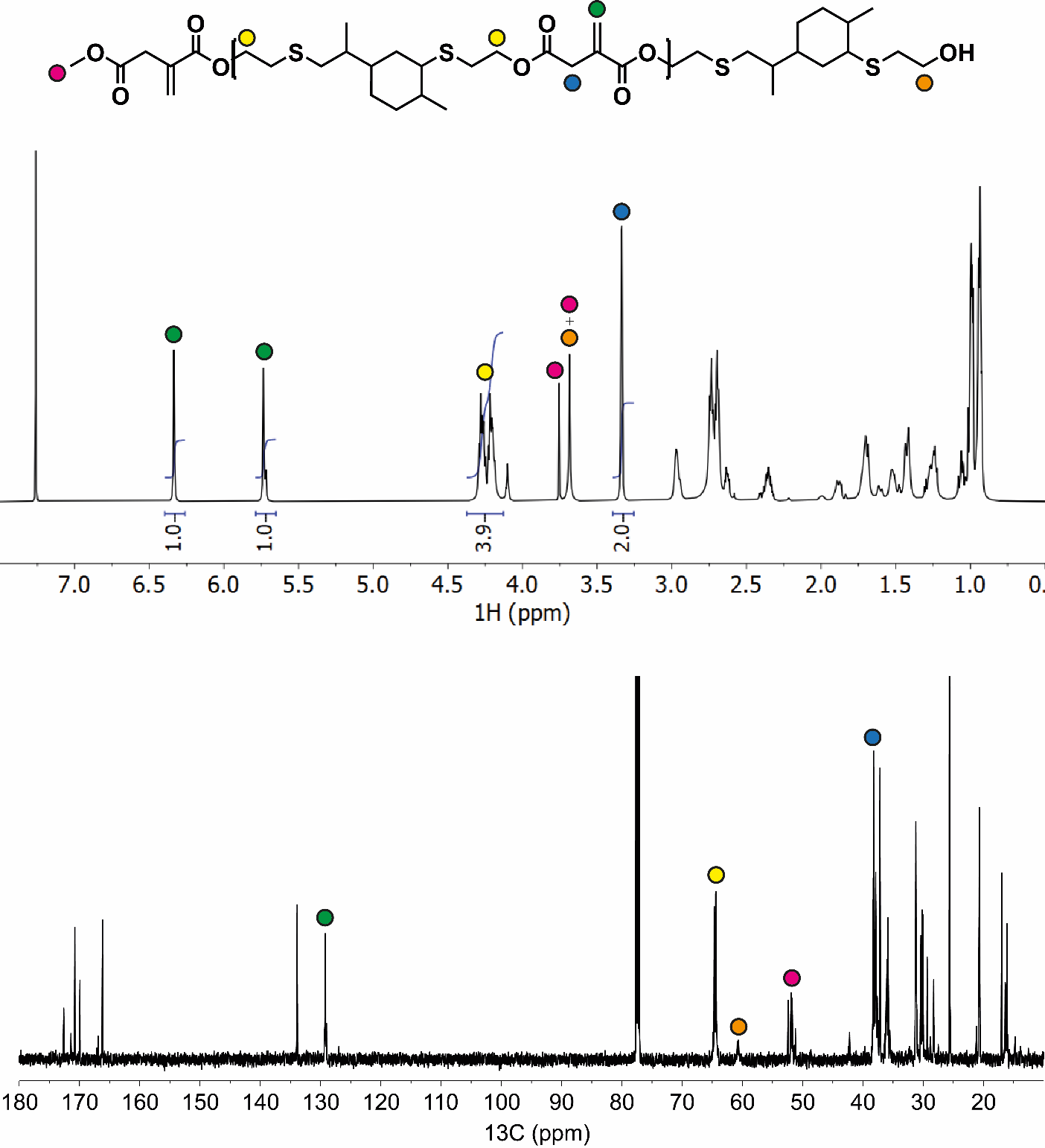


**Figure S20.** ^1^H- (600 MHz, CDCl_3_, top) and ^13^C- (125 MHz, CDCl_3_, bottom) NMR spectra of poly(ester thioether) **1a** with the spectral assignment of characteristic peaks for each monomer. By integration of the NMR signals it is possible to confirm the 1:1 ratio between **1** and itaconate units.

**
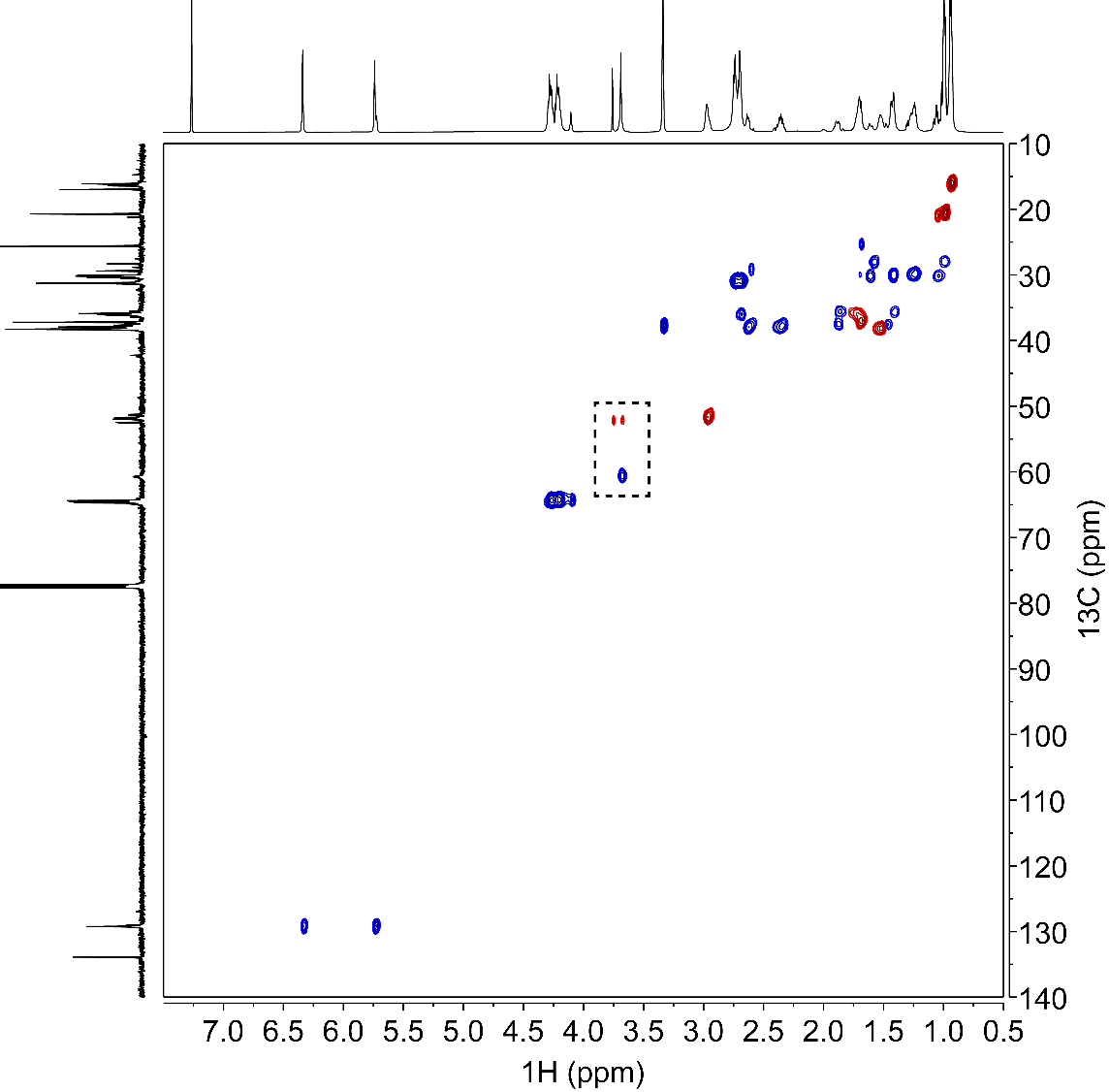
**

**Figure S21.** ^1^H-^13^C HSQC (600 MHz, CDCl_3_) NMR analysis of poly(ester thioether) **1a**. Positive peaks are coloured in red and are related to CH and CH_3_ groups, while negative peaks are coloured in blue and are related to CH_2_ groups. The peaks related to terminal monomers are highlighted in the dashed box, revealing the coexistence of terminal hydroxy- and methyl ester groups.

**
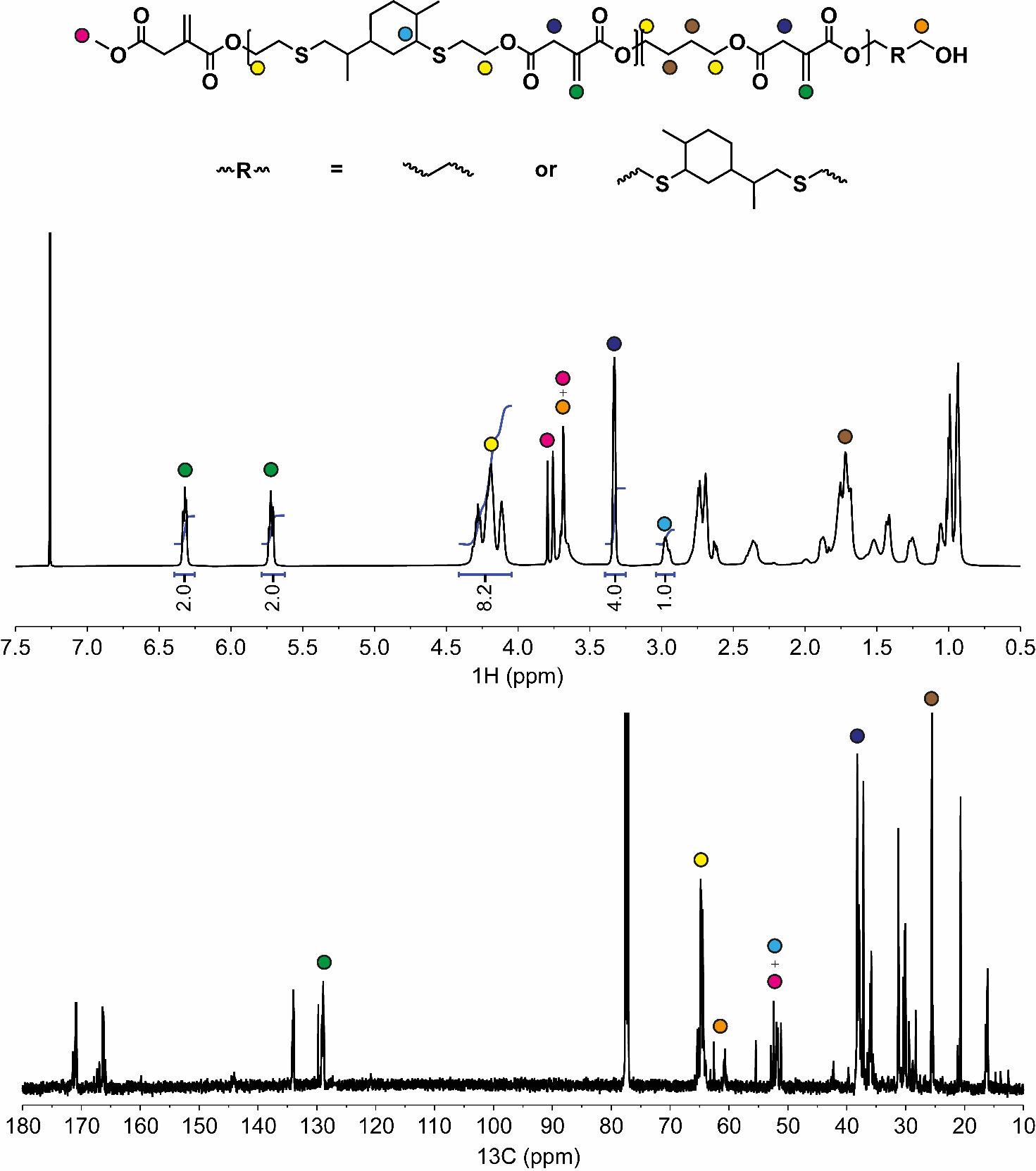
**

**Figure S22.** ^1^H- (600 MHz, CDCl_3_, top) and ^13^C- (125 MHz, CDCl_3_, bottom) NMR spectra of poly(ester thioether) **1b** with the spectral assignment of characteristic peaks for each monomer. By integration of the NMR signals it was possible to confirm the 1:1:2 ratio between **1**, 1,4-butanediol and itaconate units, respectively.


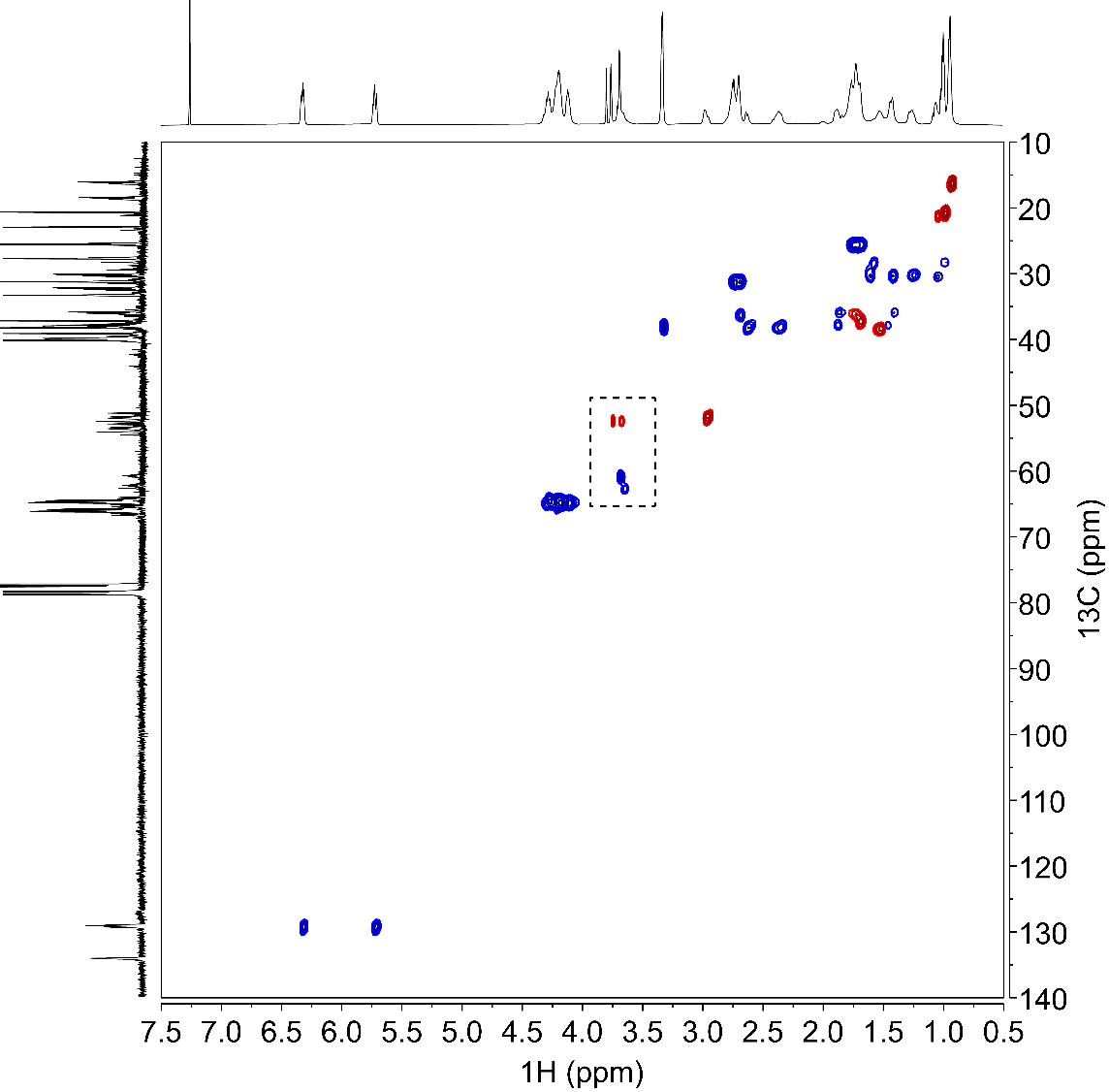


**Figure S23.** ^1^H-^13^C HSQC (600 MHz, CDCl_3_) NMR analysis of poly(ester thioether) **1b**. Positive peaks are coloured in red and are related to CH and CH_3_ groups, while negative peaks are coloured in blue and are related to CH_2_ groups. The peaks related to terminal monomers are highlighted in the dashed box, revealing the coexistence of terminal hydroxy- and methyl ester groups.


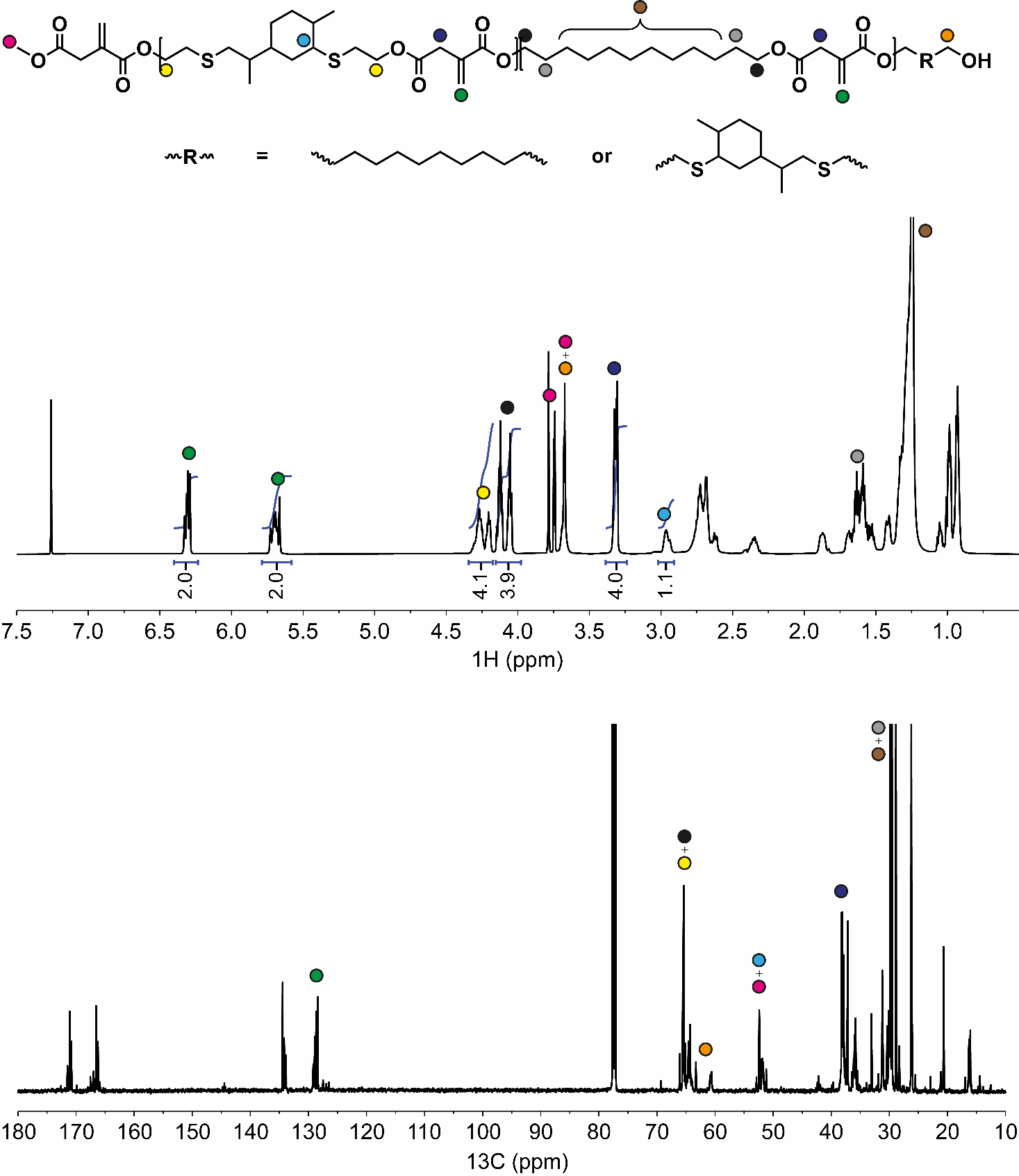


**Figure S24.** ^1^H- (600 MHz, CDCl_3_, top) and ^13^C- (125 MHz, CDCl_3_, bottom) NMR spectra of poly(ester thioether) **1c** with the spectral assignment of characteristic peaks for each monomer. By integration of the NMR signals it was possible to confirm the 1:1:2 ratio between **1**, 1,12-dodecanediol and itaconate units, respectively.

**
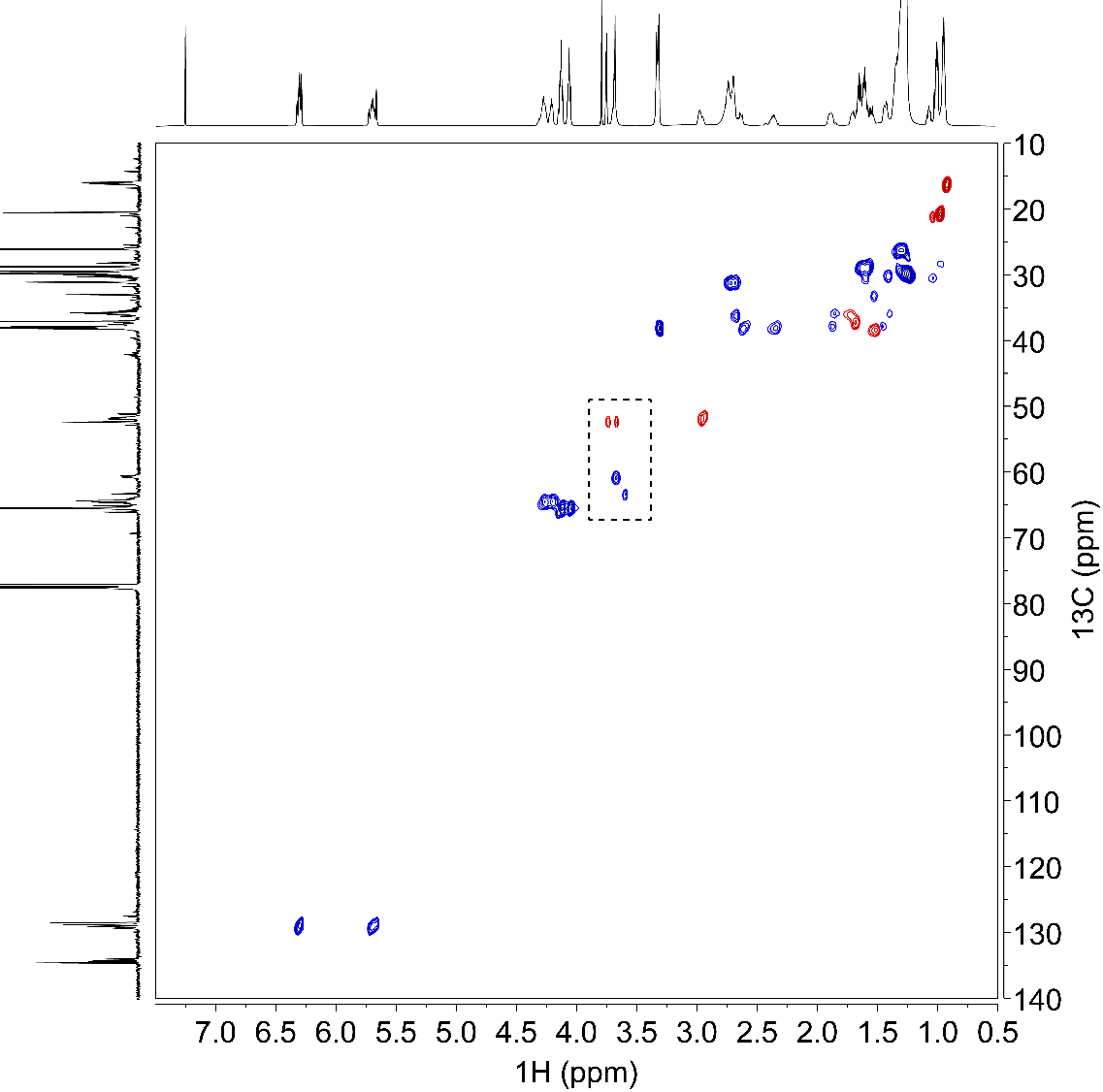
**

**Figure S25.** ^1^H-^13^C HSQC (600 MHz, CDCl_3_) NMR analysis of poly(ester thioether) **1c**. Positive peaks are coloured in red and are related to CH and CH_3_ groups, while negative peaks are coloured in blue and are related to CH_2_ groups. The peaks related to terminal monomers are highlighted in the dashed box, revealing the coexistence of terminal hydroxy- and methyl ester groups.

**
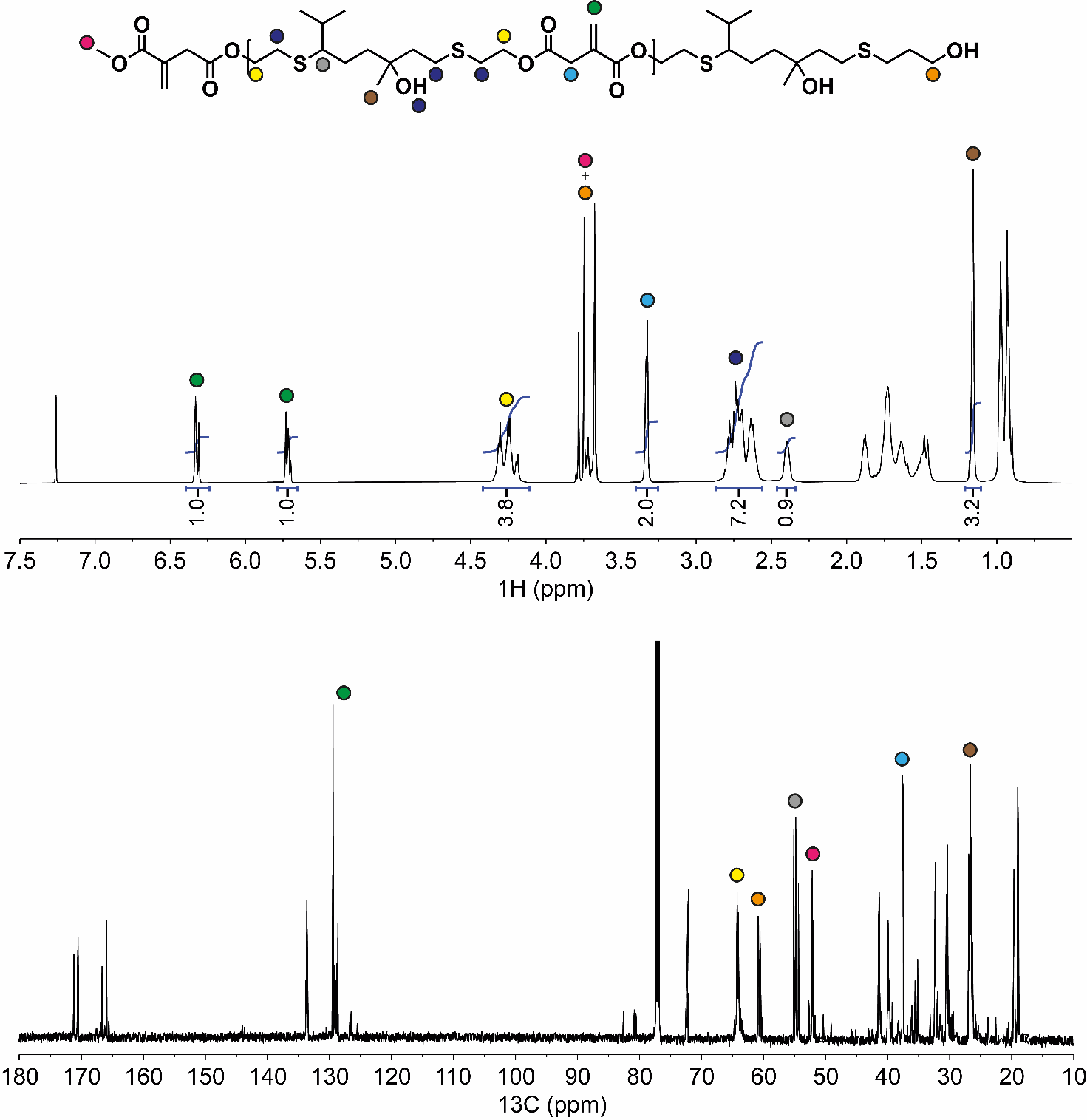
**

**Figure S26.** ^1^H- (600 MHz, CDCl_3_, top) and ^13^C- (125 MHz, CDCl_3_, bottom) NMR spectra of poly(ester thioether) **2a** with the spectral assignment of characteristic peaks for each monomer. By integration of the NMR signals it was possible to confirm the 1:1 ratio between **2** and itaconate units.

**
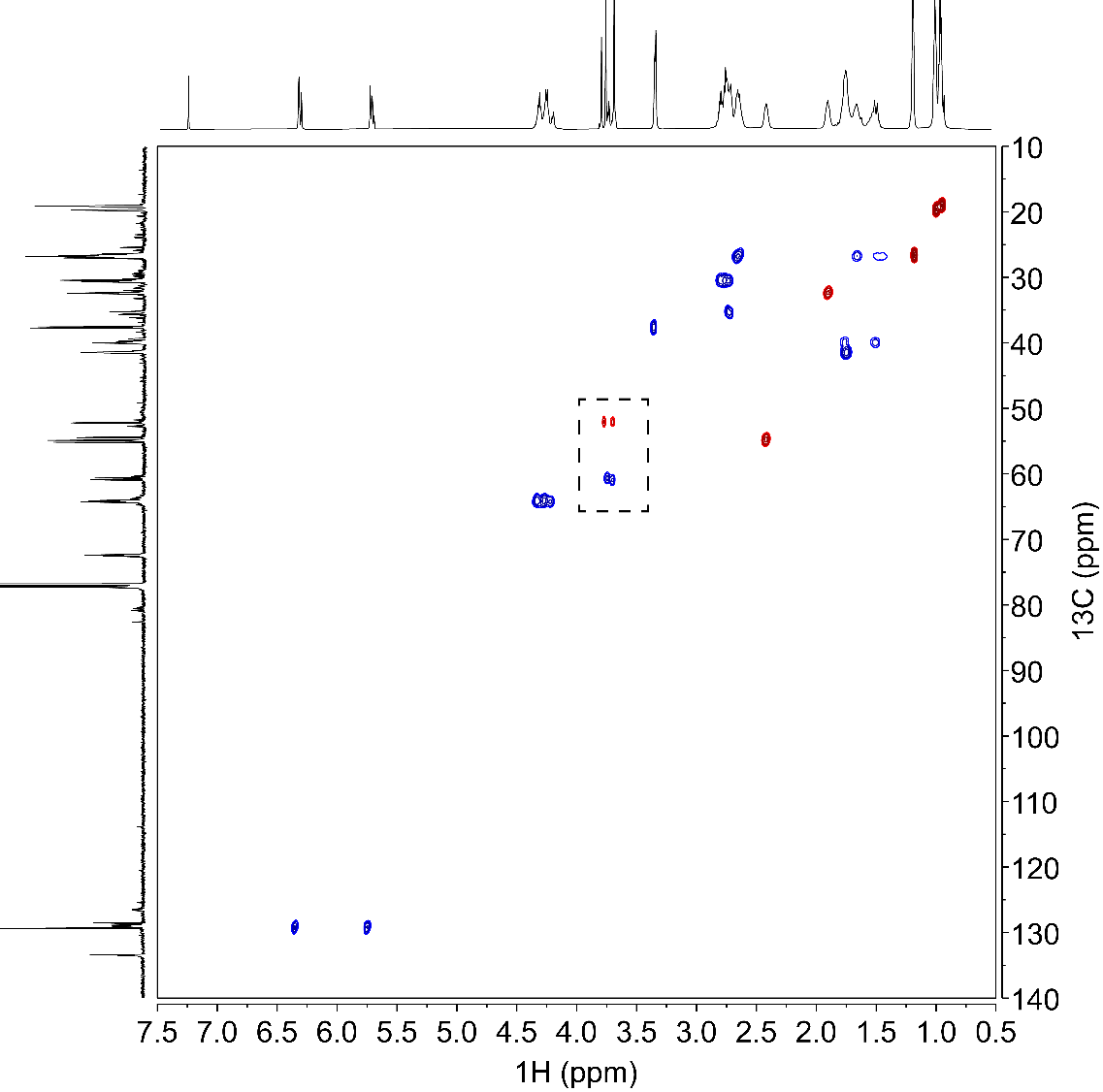
**

**Figure S27.** ^1^H-^13^C HSQC (600 MHz, CDCl_3_) NMR analysis of poly(ester thioether) **2a**. Positive peaks are coloured in red and are related to CH and CH_3_ groups, while negative peaks are coloured in blue and are related to CH_2_ groups. The peaks related to terminal monomers are highlighted in the dashed box, revealing the coexistence of terminal hydroxy- and methyl ester groups.

**
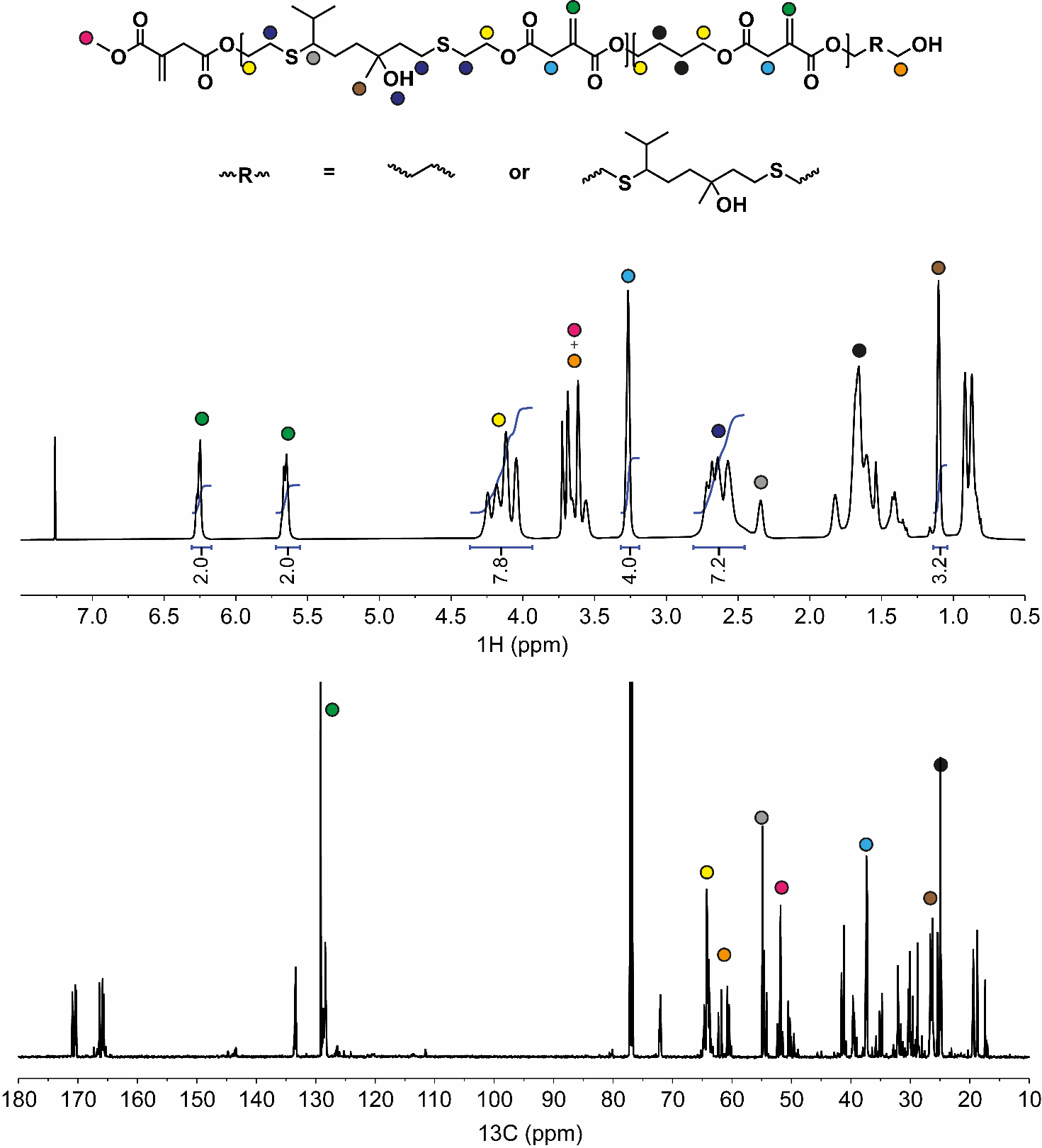
**

**Figure S28.** ^1^H- (600 MHz, CDCl_3_, top) and ^13^C- (125 MHz, CDCl_3_, bottom) NMR spectra of poly(ester thioether) **2b** with the spectral assignment of characteristic peaks for each monomer. By integration of the NMR signals it was possible to confirm the 1:1:2 ratio between **2**, 1,4-butanediol and itaconate units, respectively.

**
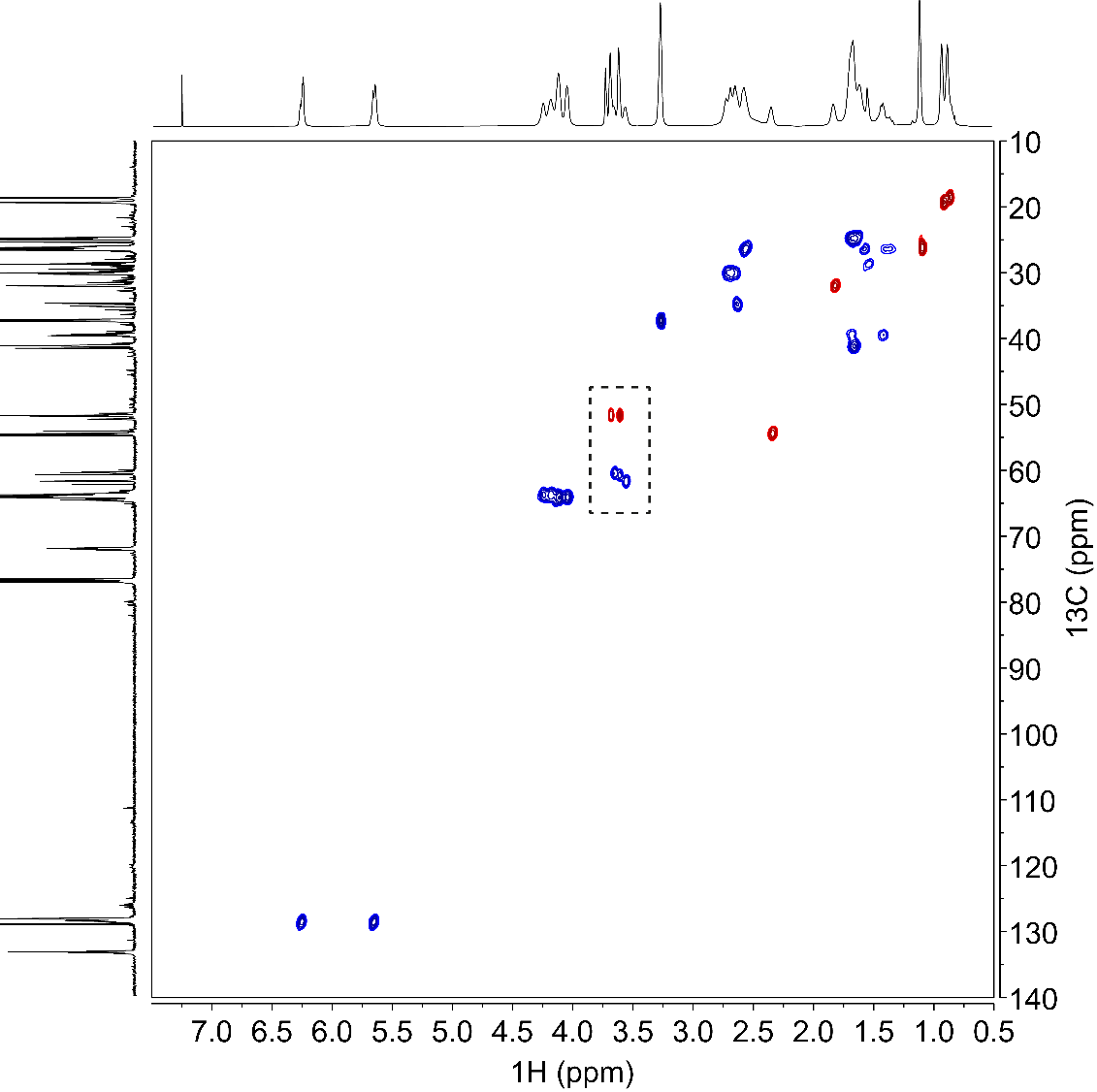
**

**Figure S29.** ^1^H-^13^C HSQC (600 MHz, CDCl_3_) NMR analysis of poly(ester thioether) **2b**. Positive peaks are coloured in red and are related to CH and CH_3_ groups, while negative peaks are coloured in blue and are related to CH_2_ groups. The peaks related to terminal monomers are highlighted in the dashed box, revealing the coexistence of terminal hydroxy- and methyl ester groups.

**
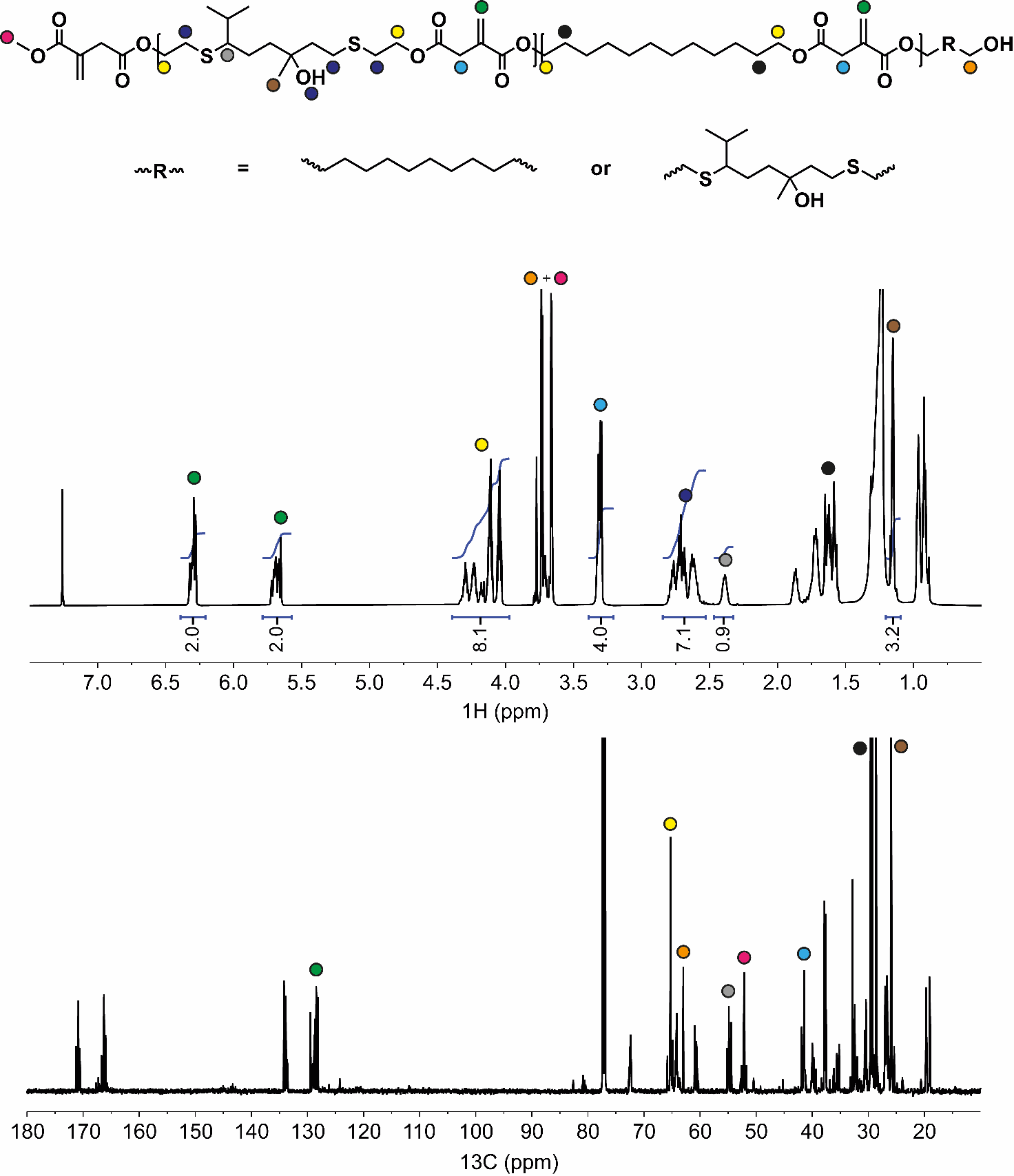
**

**Figure S30.** ^1^H- (600 MHz, CDCl_3_, top) and ^13^C- (125 MHz, CDCl_3_, bottom) NMR spectra of poly(ester thioether) **2c** with the spectral assignment of characteristic peaks for each monomer. By integration of the NMR signals it was possible to confirm the 1:1:2 ratio between **2**, 1,12-dodecanediol and itaconate units, respectively.

**
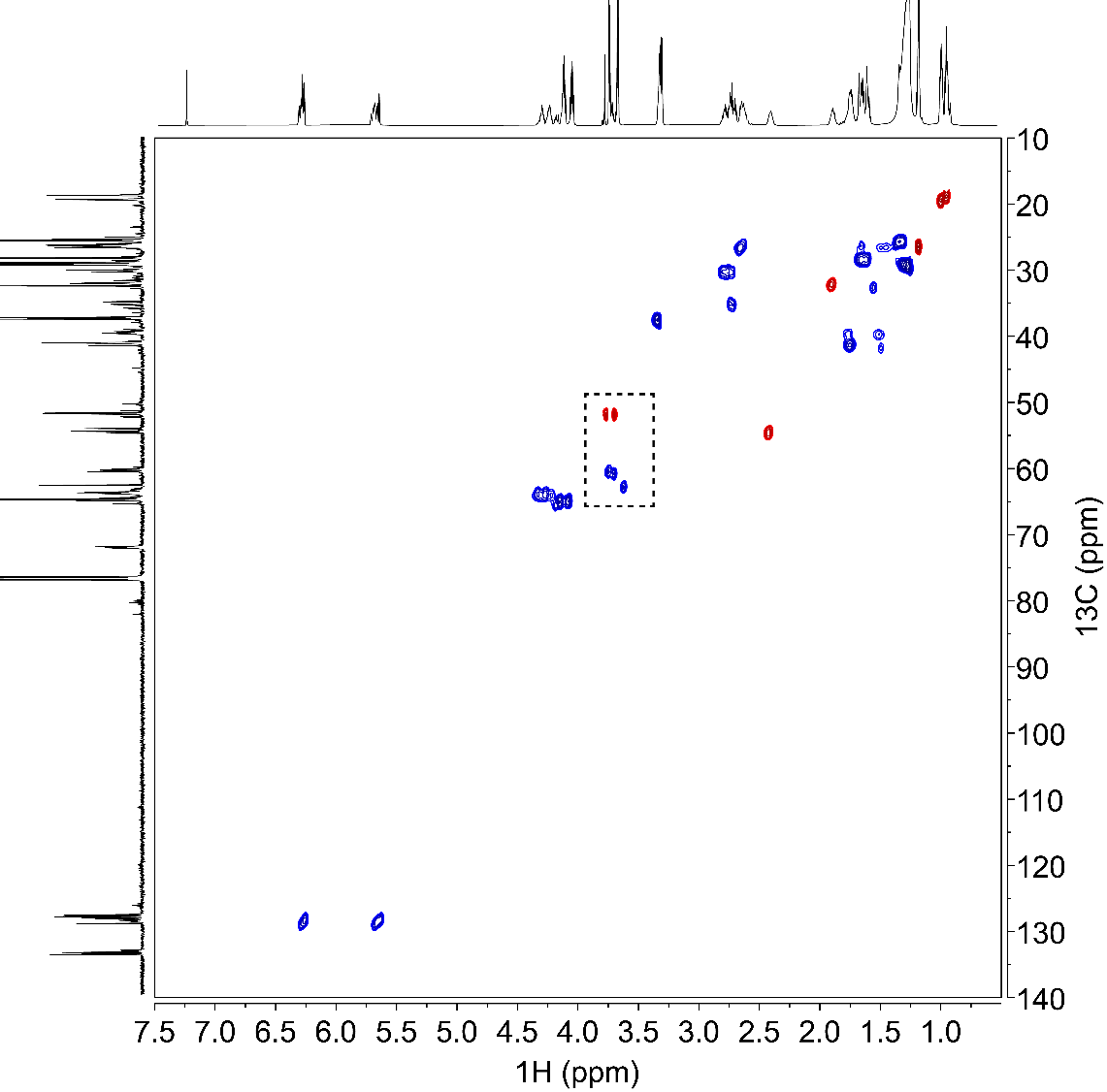
**

**Figure S31.** ^1^H-^13^C HSQC (600 MHz, CDCl_3_) NMR analysis of poly(ester thioether) **2c**. Positive peaks are coloured in red and are related to CH and CH_3_ groups, while negative peaks are coloured in blue and are related to CH_2_ groups. The peaks related to terminal monomers are highlighted in the dashed box, revealing the coexistence of terminal hydroxy- and methyl ester groups.

**
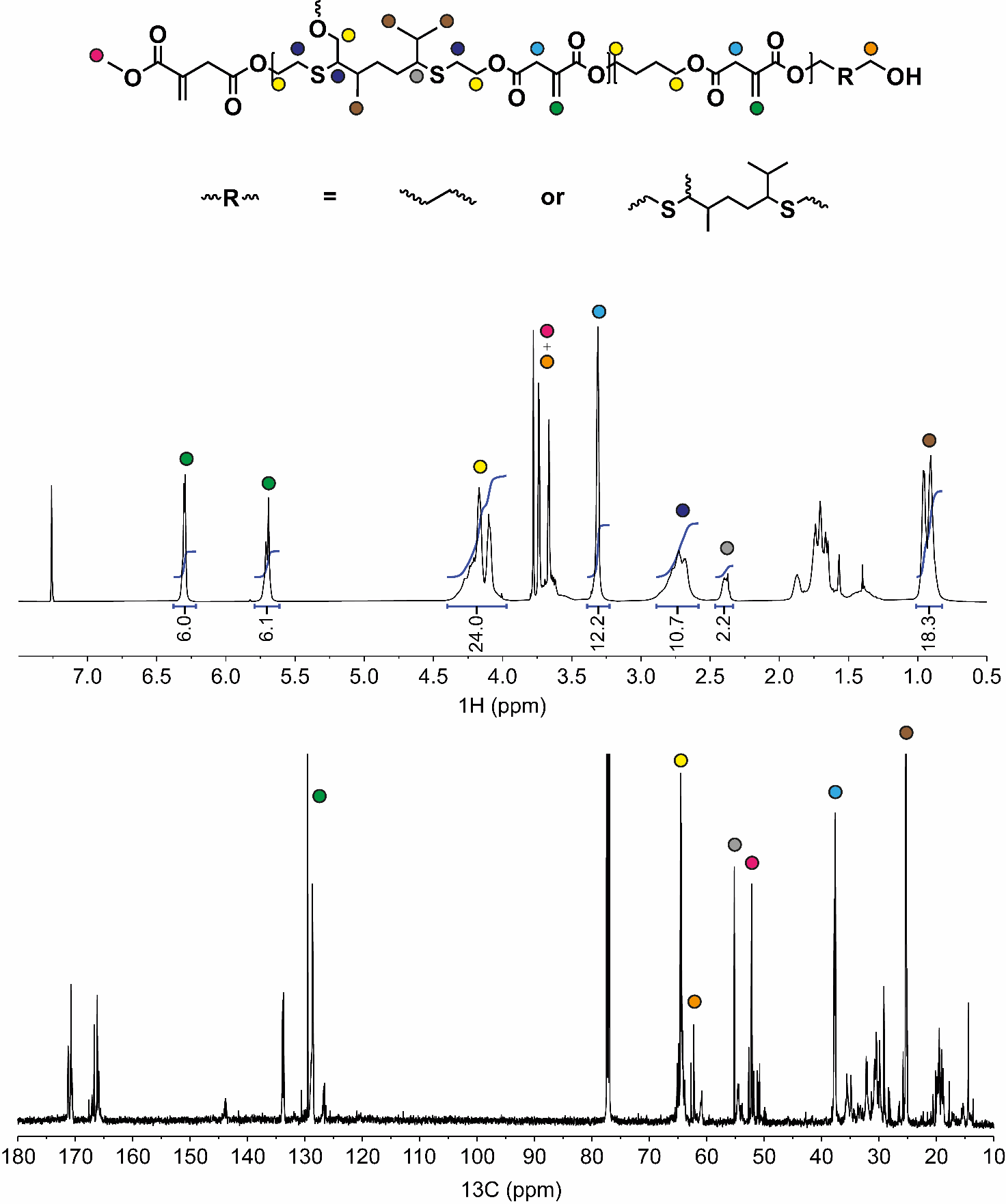
**

**Figure S32.** ^1^H- (600 MHz, CDCl_3_, top) and ^13^C- (125 MHz, CDCl_3_, bottom) NMR spectra of poly(ester thioether) **3b** with the spectral assignment of characteristic peaks for each monomer. By integration of the NMR signals it was possible to confirm the 2:3:6 ratio between **3**, 1,4-butanediol and itaconate units, respectively.

**
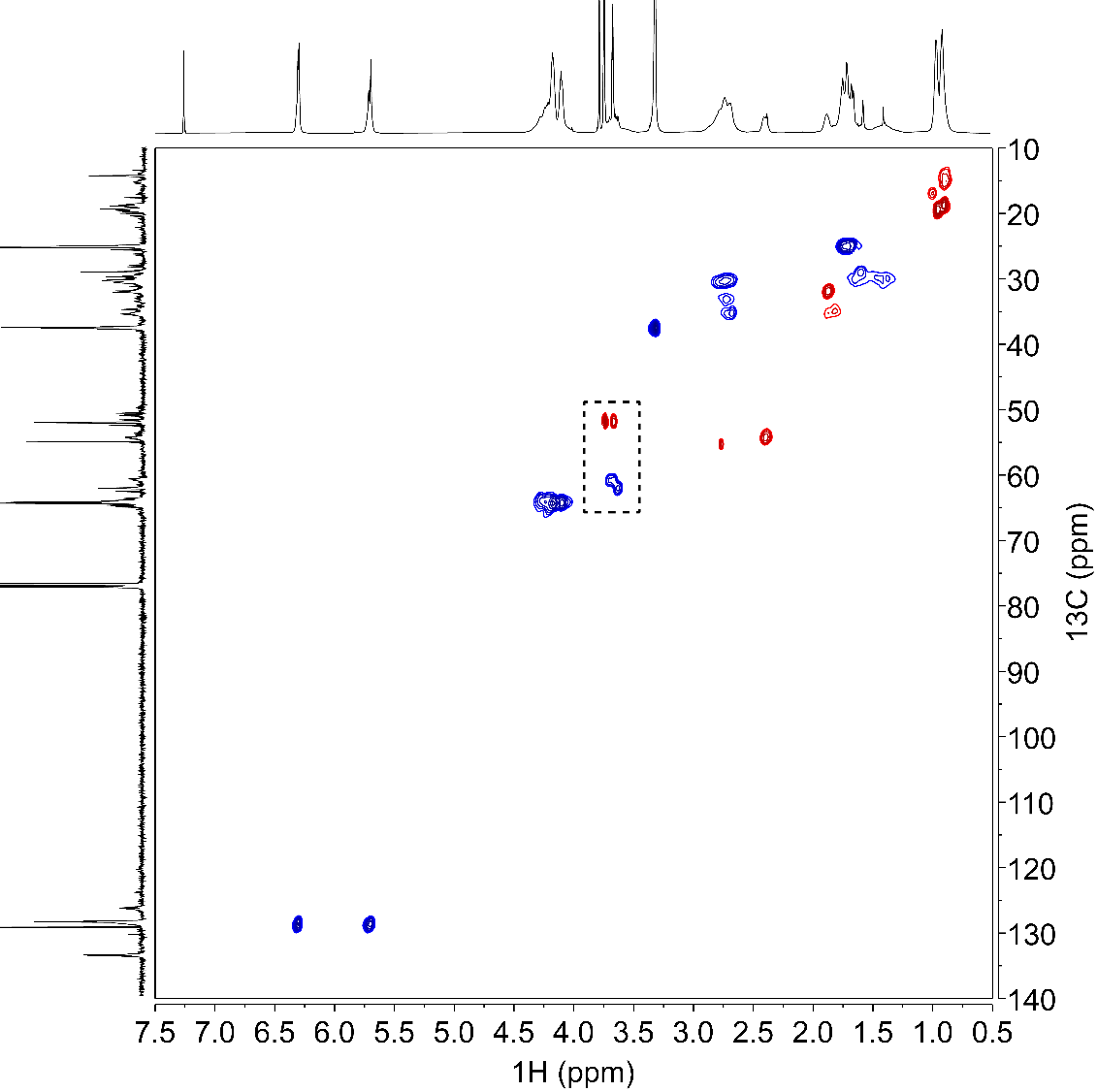
**

**Figure S33.** ^1^H-^13^C HSQC (600 MHz, CDCl_3_) NMR analysis of poly(ester thioether) **3b**. Positive peaks are coloured in red and are related to CH and CH_3_ groups, while negative peaks are coloured in blue and are related to CH_2_ groups. The peaks related to terminal monomers are highlighted in the dashed box, revealing the coexistence of terminal hydroxy- and methyl ester groups.

**
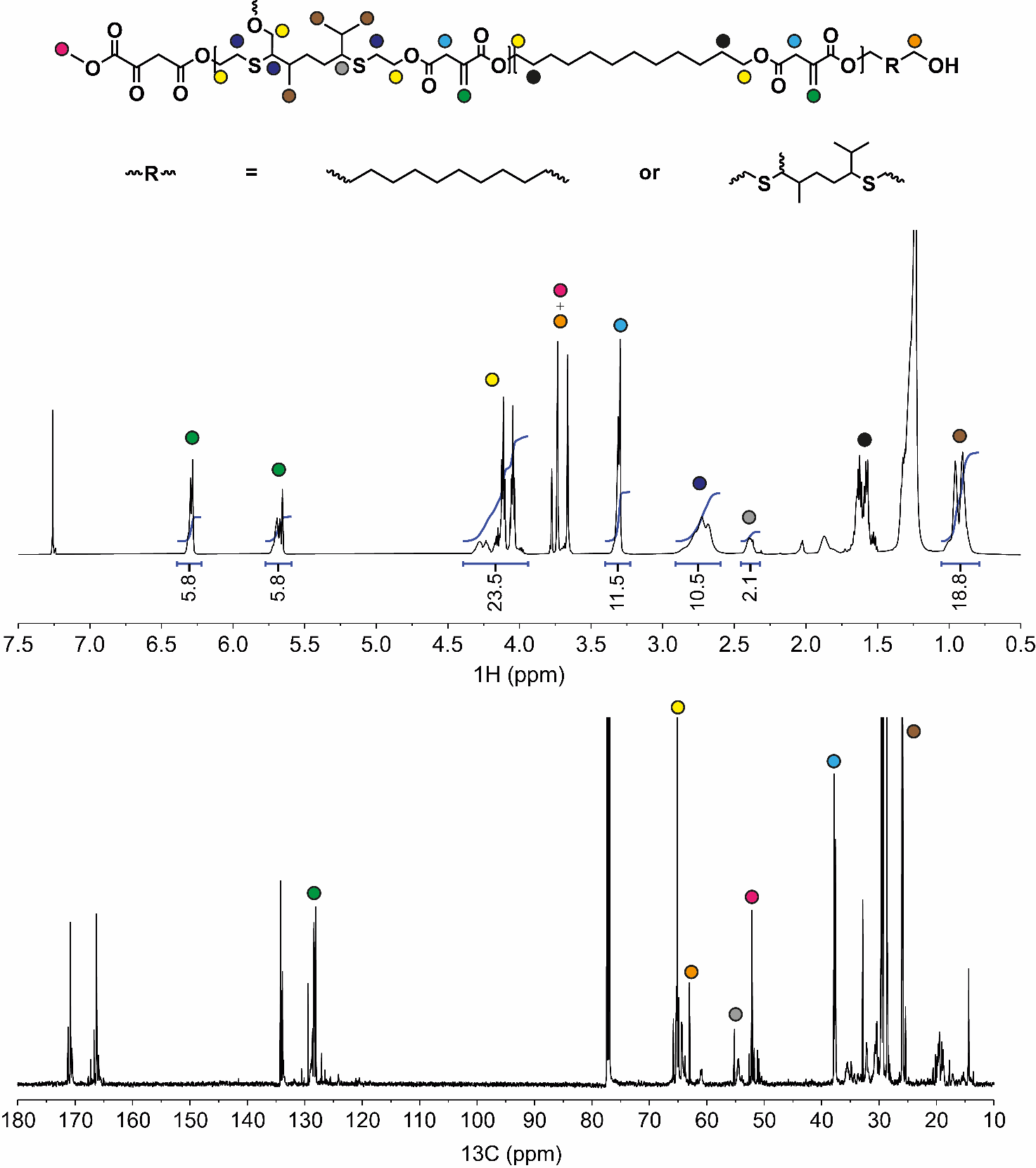
**

**Figure S34.** ^1^H- (600 MHz, CDCl_3_, top) and ^13^C- (125 MHz, CDCl_3_, bottom) NMR spectra of poly(ester thioether) **3c** with the spectral assignment of characteristic peaks for each monomer. By integration of the NMR signals it was possible to confirm the 2:3:6 ratio between **3**, 1,12-dodecanediol and itaconate units, respectively.

**
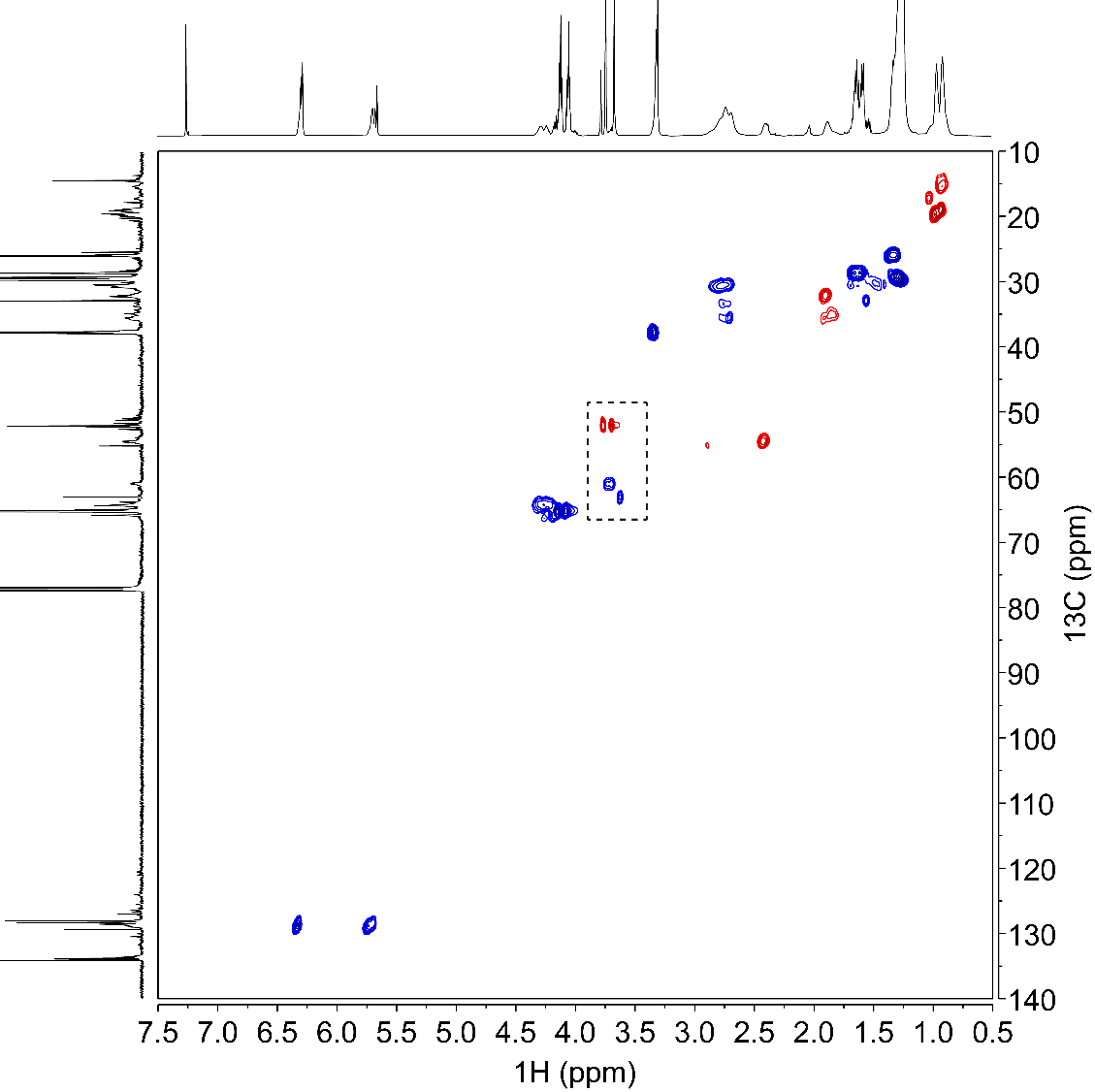
**

**Figure S35.** ^1^H-^13^C HSQC (600 MHz, CDCl_3_) NMR analysis of poly(ester thioether) **3c**. Positive peaks are coloured in red and are related to CH and CH_3_ groups, while negative peaks are coloured in blue and are related to CH_2_ groups. The peaks related to terminal monomers are highlighted in the dashed box, revealing the coexistence of terminal hydroxy- and methyl ester groups.


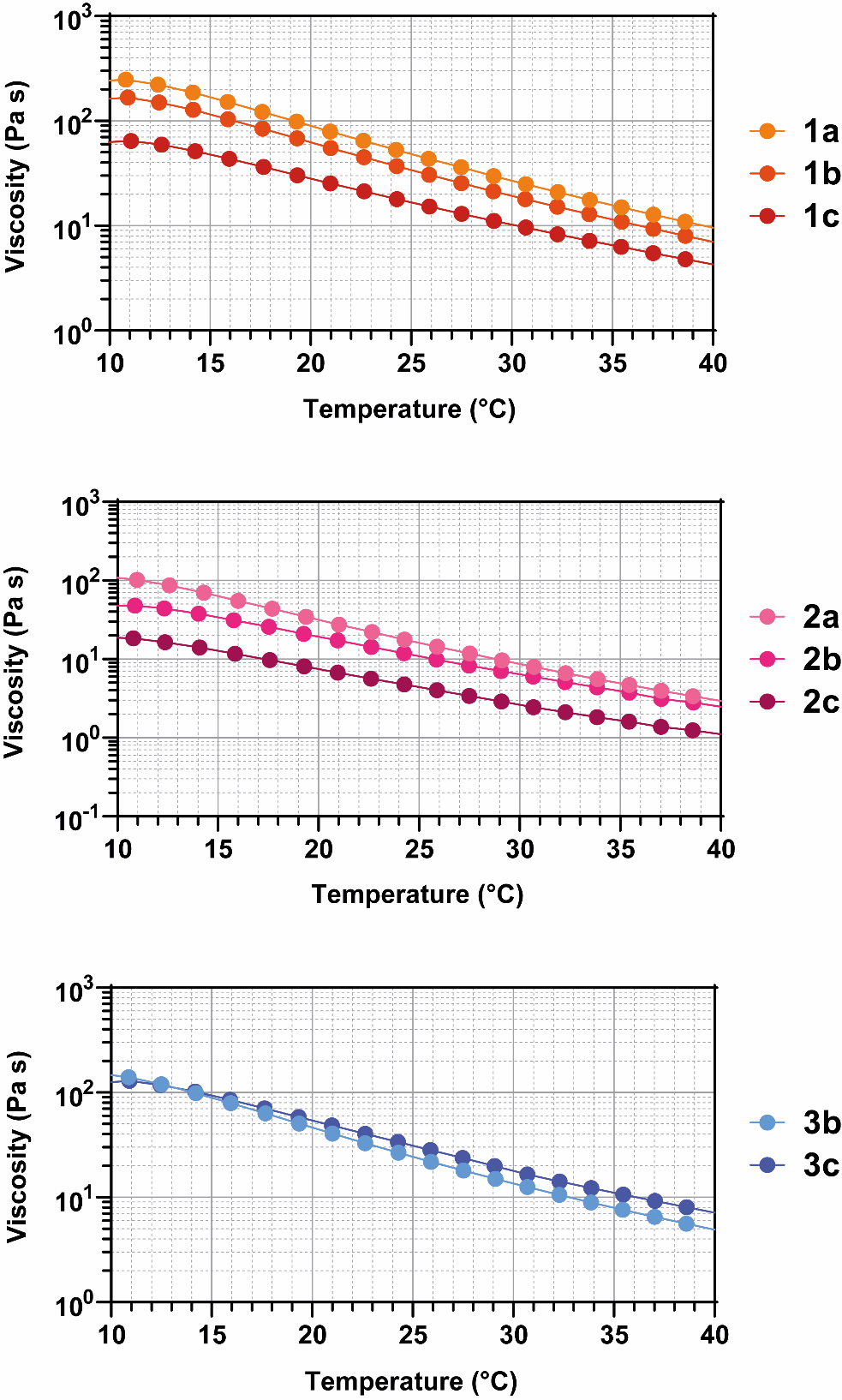


**Figure S36**. Rotational viscosity measurements (shear rate = 1 Hz) for the prepared poly(ester-thioethers) as a function of temperature


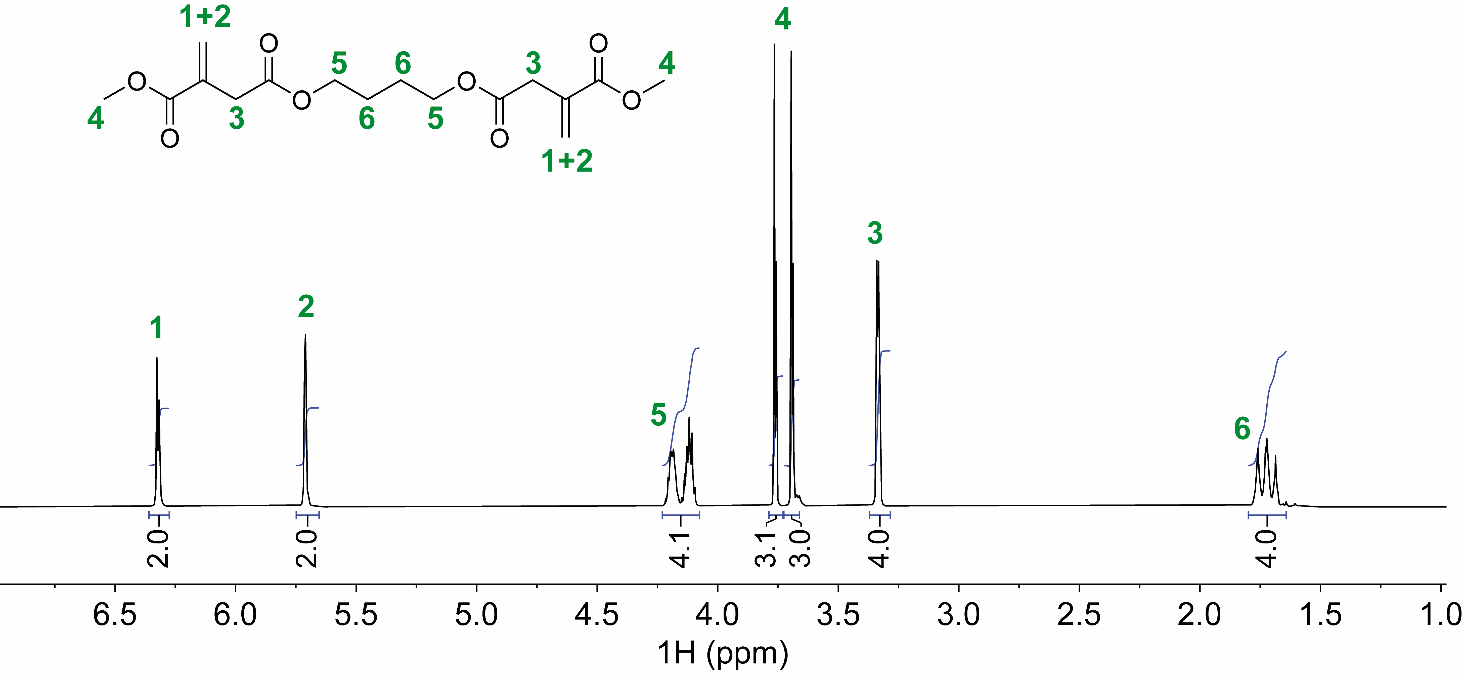


**Figure S37.** ^1^H-NMR spectrum of 1,4-butanediyl bis(methyl itaconate) with the corresponding chemical structure and peak assignments.


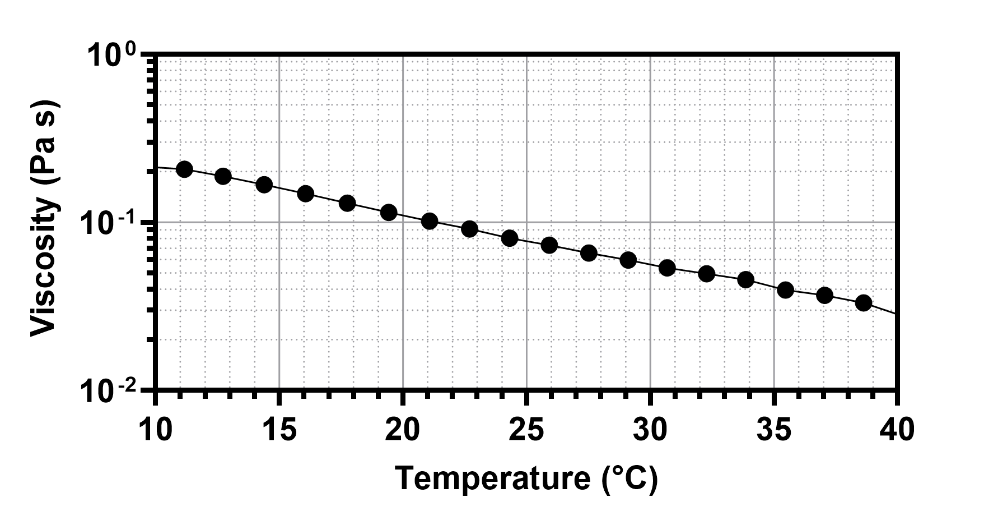


**Figure S38.** Viscosity of I_2_B_1_ as a function of temperature.


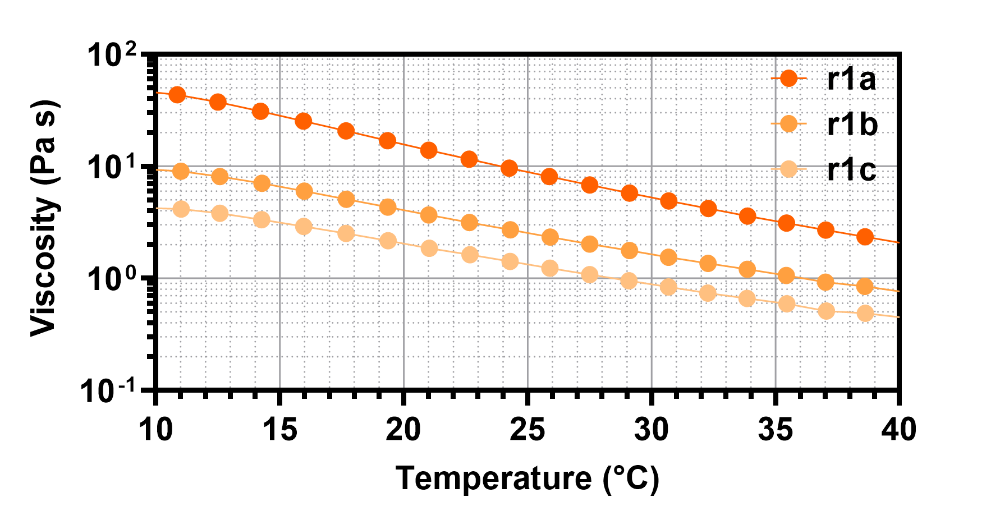

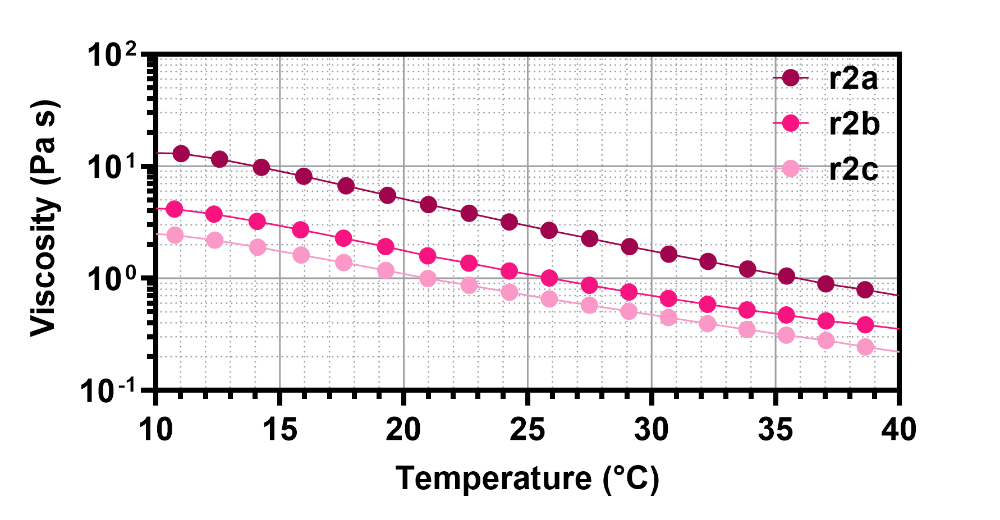

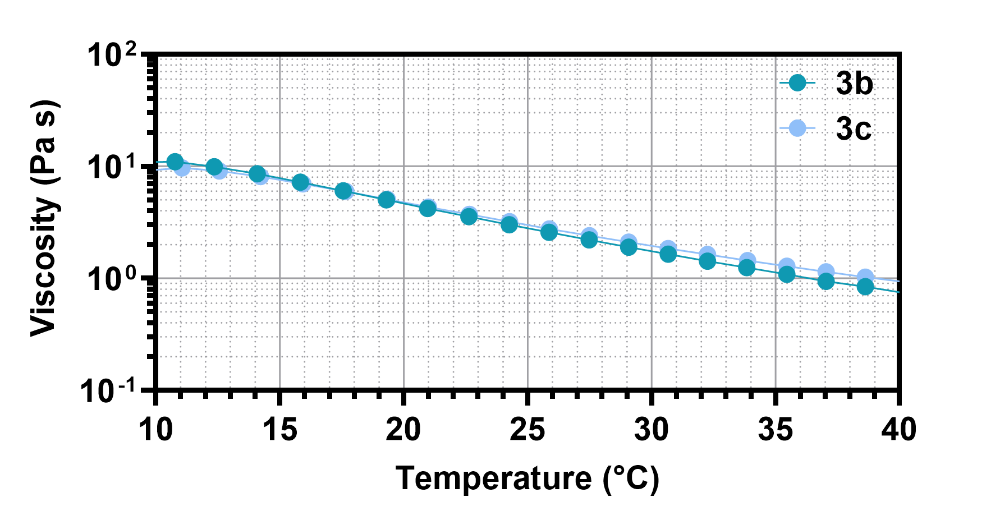


**Figure S39**. Viscosity of the described photocurable formulations for stereolithography as a function of temperature

**Quantification of the biobased content**

To be able to calculate the biobased carbon content of the photocurable formulations in terms of their overall biomass content and their total biobased carbon content, it is firstly necessary to determine for each component of the formulations: molecular weight, total number of carbon atoms, and number of biobased carbon atoms, For polymers, this is related to the repeating unit that contains the small integer number of all monomers. In particular:

**Polymer 1a**

Itaconic acid 50% Molecular weight = 112

Thioether polyol **1** 50% Molecular weight = 274

Molecular weigth of repeating unit = 386

Total number of carbon atoms = 19

Number of biobased carbon atoms = 15

**Polymer 1b**

Itaconic acid 50% Molecular weight = 112

Thioether polyol **1** 25% Molecular weight = 274

1,4-butanediol 25% Molecular weigth = 72

Molecular weigth of repeating unit = 570

Total number of carbon atoms = 28

Number of biobased carbon atoms = 24

**Polymer 1c**

Itaconic acid 50% Molecular weight = 112

Thioether polyol **1** 25% Molecular weight = 274

1,12-dodecanediol 25% Molecular weigth = 184

Molecular weigth of repeating unit = 682

Total number of carbon atoms = 36

Number of biobased carbon atoms = 32

**Polymer 2a**

Itaconic acid 50% Molecular weight = 112

Thioether polyol **2** 50% Molecular weight = 292

Molecular weigth of repeating unit = 404

Total number of carbon atoms = 19

Number of biobased carbon atoms = 15

**Polymer 2b**

Itaconic acid 50% Molecular weight = 112

Thioether polyol **2** 25% Molecular weight = 292

1,4-butanediol 25% Molecular weigth = 72

Molecular weigth of repeating unit = 588

Total number of carbon atoms = 28

Number of biobased carbon atoms = 24

**Polymer 2c**

Itaconic acid 50% Molecular weight = 112

Thioether polyol **2** 25% Molecular weight = 292

1,12-dodecanediol 25% Molecular weigth = 184

Molecular weigth of repeating unit = 700

Total number of carbon atoms = 36

Number of biobased carbon atoms = 32

**Polymer 3b**

Itaconic acid 55% Molecular weight = 112

Thioether polyol **3** 18% Molecular weight = 292

1,4-butanediol 27% Molecular weigth = 72

Molecular weigth of repeating unit = 1454

Total number of carbon atoms = 70

Number of biobased carbon atoms = 62

**Polymer 3c**

Itaconic acid 55% Molecular weight = 112

Thioether polyol **3** 18% Molecular weight = 292

1,12-dodecanediol 27% Molecular weigth = 184

Molecular weigth of repeating unit = 1790

Total number of carbon atoms = 94

Number of biobased carbon atoms = 86

**1,4-butanediyl bis(methyl itaconate) - I_2_B_1_**

Molecular weigth = 342

Total number of carbon atoms = 16

Number of biobased carbon atoms = 16

**9-hydroxystearic acid monoglyceride triacetate - SNS**

Molecular weigth = 500

Total number of carbon atoms = 27

Number of biobased carbon atoms = 27

**Ethyl phenyl(2,4,6-trimethylbenzoyl)phosphinate - Et-APO**

Molecular weigth = 316

Total number of carbon atoms = 18

Number of biobased carbon atoms = 0

**BHT**

Molecular weigth = 220

Total number of carbon atoms = 15

Number of biobased carbon atoms = 0

**2- isopropyl thioxanthone - ITX**

Molecular weigth = 254

Total number of carbon atoms = 16

Number of biobased carbon atoms = 0

At this point, for each formulation, the following descriptors are calculated:

- ***BIOBASED MASS***: calculated for each component of the formulations, it represents the percentage of the mass of the component that is biobased or it is obtained by renewable resources. It is 0% for Et-APO, BHT and ITX, 100% for I_2_B_1_ and SNS while for each polymer it is calculated as:

$$\boldsymbol{BIOBASED MASS}=\frac{M_{w}-n\cdot ME}{M_{w}}$$

Where $M_{w}$ is the molecular weight of the repeating unit, $n$ is the number of 2-mercaptoethanol groups in the repeating unit and $ME$ is the molecular weight of 2-mercaptoethanol (78 g/mol).

- ***Total carbon content (X^TC^***): for each component of the formulation, the elemental composition by weight is calculated for carbon.
- ***Biobased carbon content***: for each component of the formulation, the biobased carbon content is calculated as the ratio between the number of biobased carbon atoms and the total number of carbon atoms.
- ***Carbon mass***: represent the mass of biobased and non-biobased carbon in 100 parts of the photocurable resin, coming from each of the different components of the formulation.

Photocurable composition **r1a**

|  |  |  |  |  | **Carbon mass** | |
| --- | --- | --- | --- | --- | --- | --- |
|  |  | **Biobased**  **mass** | **X^TC^** | **Biobased**  **Carbon Content** | **Biobased** | **Non-biobased** |
| **1a** | 60% | 59.6% | 59% | 79% | 28.0 | 7.5 |
| **I_2_B_1_** | 29% | 100% | 56% | 100% | 16.1 | 0.0 |
| **SNS** | 10% | 100% | 65% | 100% | 6.5 | 0.0 |
| **Et-APO** | 0.5% | 0% | 68% | 0% | 0.0 | 0.3 |
| **ITX** | 0.3% | 0% | 76% | 0% | 0.0 | 0.2 |
| **BHT** | 0.5% | 0% | 82% | 0% | 0.0 | 0.4 |

Photocurable composition **r1b**

|  |  |  |  |  | **Carbon mass** | |
| --- | --- | --- | --- | --- | --- | --- |
|  |  | **Biobased**  **mass** | **X^TC^** | **Biobased**  **Carbon Content** | **Biobased** | **Non-biobased** |
| **1b** | 60% | 72.6% | 59% | 86% | 30.3 | 5.1 |
| **I2B1** | 29% | 100% | 56% | 100% | 16.1 | 0.0 |
| **SNS** | 10% | 100% | 65% | 100% | 6.5 | 0.0 |
| **Et-APO** | 0.5% | 0% | 68% | 0% | 0.0 | 0.3 |
| **ITX** | 0.3% | 0% | 76% | 0% | 0.0 | 0.2 |
| **BHT** | 0.5% | 0% | 82% | 0% | 0.0 | 0.4 |

Photocurable composition **r1c**

|  |  |  |  |  | **Carbon mass** | |
| --- | --- | --- | --- | --- | --- | --- |
|  |  | **Biobased**  **mass** | **X^TC^** | **Biobased**  **Carbon Content** | **Biobased** | **Non-biobased** |
| **1c** | 60% | 77.1% | 63% | 89% | 33.8 | 4.2 |
| **I2B1** | 29% | 100% | 56% | 100% | 16.1 | 0.0 |
| **SNS** | 10% | 100% | 65% | 100% | 6.5 | 0.0 |
| **Et-APO** | 0.5% | 0% | 68% | 0% | 0.0 | 0.3 |
| **ITX** | 0.3% | 0% | 76% | 0% | 0.0 | 0.2 |
| **BHT** | 0.5% | 0% | 82% | 0% | 0.0 | 0.4 |

Photocurable composition **r2a**

|  |  |  |  |  | **Carbon mass** | |
| --- | --- | --- | --- | --- | --- | --- |
|  |  | **Biobased**  **mass** | **X^TC^** | **Biobased**  **Carbon Content** | **Biobased** | **Non-biobased** |
| **2a** | 60% | 61.4% | 56% | 79% | 26.7 | 7.1 |
| **I2B1** | 29% | 100% | 56% | 100% | 16.1 | 0.0 |
| **SNS** | 10% | 100% | 65% | 100% | 6.5 | 0.0 |
| **Et-APO** | 0.5% | 0% | 68% | 0% | 0.0 | 0.3 |
| **ITX** | 0.3% | 0% | 76% | 0% | 0.0 | 0.2 |
| **BHT** | 0.5% | 0% | 82% | 0% | 0.0 | 0.4 |

Photocurable composition **r2b**

|  |  |  |  |  | **Carbon mass** | |
| --- | --- | --- | --- | --- | --- | --- |
|  |  | **Biobased**  **mass** | **X^TC^** | **Biobased**  **Carbon Content** | **Biobased** | **Non-biobased** |
| **2b** | 60% | 73.5% | 57% | 86% | 29.4 | 4.9 |
| **I2B1** | 29% | 100% | 56% | 100% | 16.1 | 0.0 |
| **SNS** | 10% | 100% | 65% | 100% | 6.5 | 0.0 |
| **Et-APO** | 0.5% | 0% | 68% | 0% | 0.0 | 0.3 |
| **ITX** | 0.3% | 0% | 76% | 0% | 0.0 | 0.2 |
| **BHT** | 0.5% | 0% | 82% | 0% | 0.0 | 0.4 |

Photocurable composition **r2c**

|  |  |  |  |  | **Carbon mass** | |
| --- | --- | --- | --- | --- | --- | --- |
|  |  | **Biobased**  **mass** | **X^TC^** | **Biobased**  **Carbon Content** | **Biobased** | **Non-biobased** |
| **2c** | 60% | 77.7% | 62% | 89% | 32.9 | 4.1 |
| **I2B1** | 29% | 100% | 56% | 100% | 16.1 | 0.0 |
| **SNS** | 10% | 100% | 65% | 100% | 6.5 | 0.0 |
| **Et-APO** | 0.5% | 0% | 68% | 0% | 0.0 | 0.3 |
| **ITX** | 0.3% | 0% | 76% | 0% | 0.0 | 0.2 |
| **BHT** | 0.5% | 0% | 82% | 0% | 0.0 | 0.4 |

Photocurable composition **r3b**

|  |  |  |  |  | **Carbon mass** | |
| --- | --- | --- | --- | --- | --- | --- |
|  |  | **Biobased**  **mass** | **X^TC^** | **Biobased**  **Carbon Content** | **Biobased** | **Non-biobased** |
| **3b** | 60% | 78.5% | 58% | 89% | 30.7 | 4.0 |
| **I2B1** | 29% | 100% | 56% | 100% | 16.1 | 0.0 |
| **SNS** | 10% | 100% | 65% | 100% | 6.5 | 0.0 |
| **Et-APO** | 0.5% | 0% | 68% | 0% | 0.0 | 0.3 |
| **ITX** | 0.3% | 0% | 76% | 0% | 0.0 | 0.2 |
| **BHT** | 0.5% | 0% | 82% | 0% | 0.0 | 0.4 |

Photocurable composition **3c**

|  |  |  |  |  | **Carbon mass** | |
| --- | --- | --- | --- | --- | --- | --- |
|  |  | **Biobased**  **mass** | **X^TC^** | **Biobased**  **Carbon Content** | **Biobased** | **Non-biobased** |
| **3c** | 60% | 82.6% | 63% | 91% | 34.6 | 3.2 |
| **I2B1** | 29% | 100% | 56% | 100% | 16.1 | 0.0 |
| **SNS** | 10% | 100% | 65% | 100% | 6.5 | 0.0 |
| **Et-APO** | 0.5% | 0% | 68% | 0% | 0.0 | 0.3 |
| **ITX** | 0.3% | 0% | 76% | 0% | 0.0 | 0.2 |
| **BHT** | 0.5% | 0% | 82% | 0% | 0.0 | 0.4 |

From this values it is possible to calculate the values for the following descriptors according to the TUV regulation for the OK BIOBASED labelling:

- $\mathbf{m}_{\mathbf{B}}$: bio-based content, expressed as a percentage of the total mass of the sample;
- $\mathbf{X}_{\mathbf{B}}^{\mathbf{TC}}$: bio-based carbon content by total carbon, i.e. the ratio between the total mass of biobased carbon in the formulation and the total carbon content of the formulation;
- $\mathbf{X}^{\mathbf{TC}}$: total carbon content of the formulations, expressed as total weight percentage of the element in the formulation.

| **Formulation #** | $\mathbf{m}_{\mathbf{B}}$ | $\mathbf{X}_{\mathbf{B}}^{\mathbf{TC}}$ | $\mathbf{X}^{\mathbf{TC}}$ |
| --- | --- | --- | --- |
| **r1a** | 74.5% | **85.7%** | 59.0% |
| **r1b** | 82.3% | **89.8%** | 58.9% |
| **r1c** | 85.0% | **91.6%** | 61.6% |
| **r2a** | 75.5% | **85.9%** | 57.4% |
| **r2b** | 82.8% | **89.8%** | 57.9% |
| **r2c** | 85.3% | **91.6%** | 60.6% |
| **r3b** | 85.8% | **91.5%** | 58.2% |
| **r3c** | 88.2% | **93.2%** | 61.4% |

**Table S4**. Calculated parameters for the evaluation of the biobased carbon content and the overall biomass content of the formulated resins.


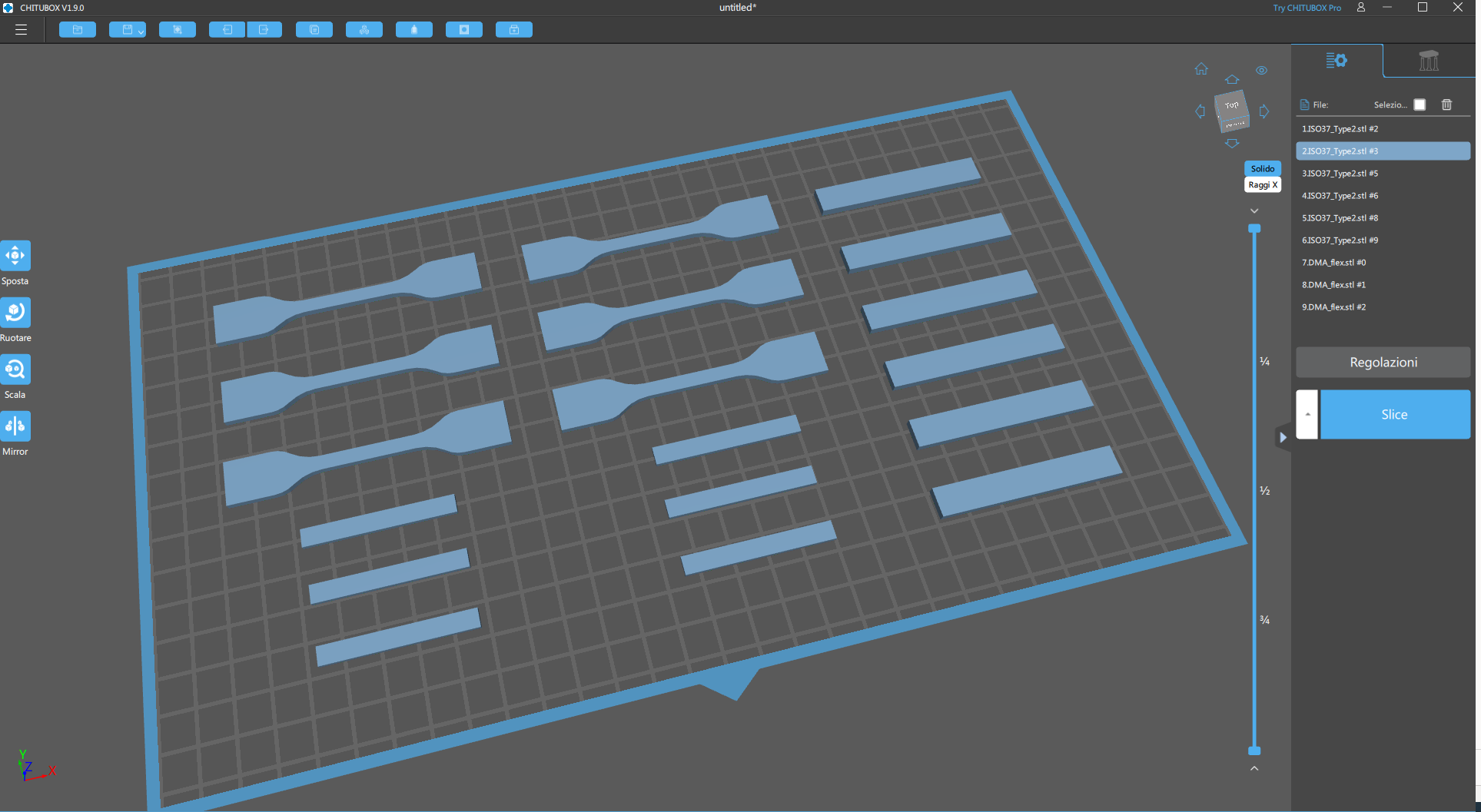


**Figure S40**. Printed objects arrangement and slicing performed using the Chitubox 1.9.4 software for tensile and DMA tests specimen.


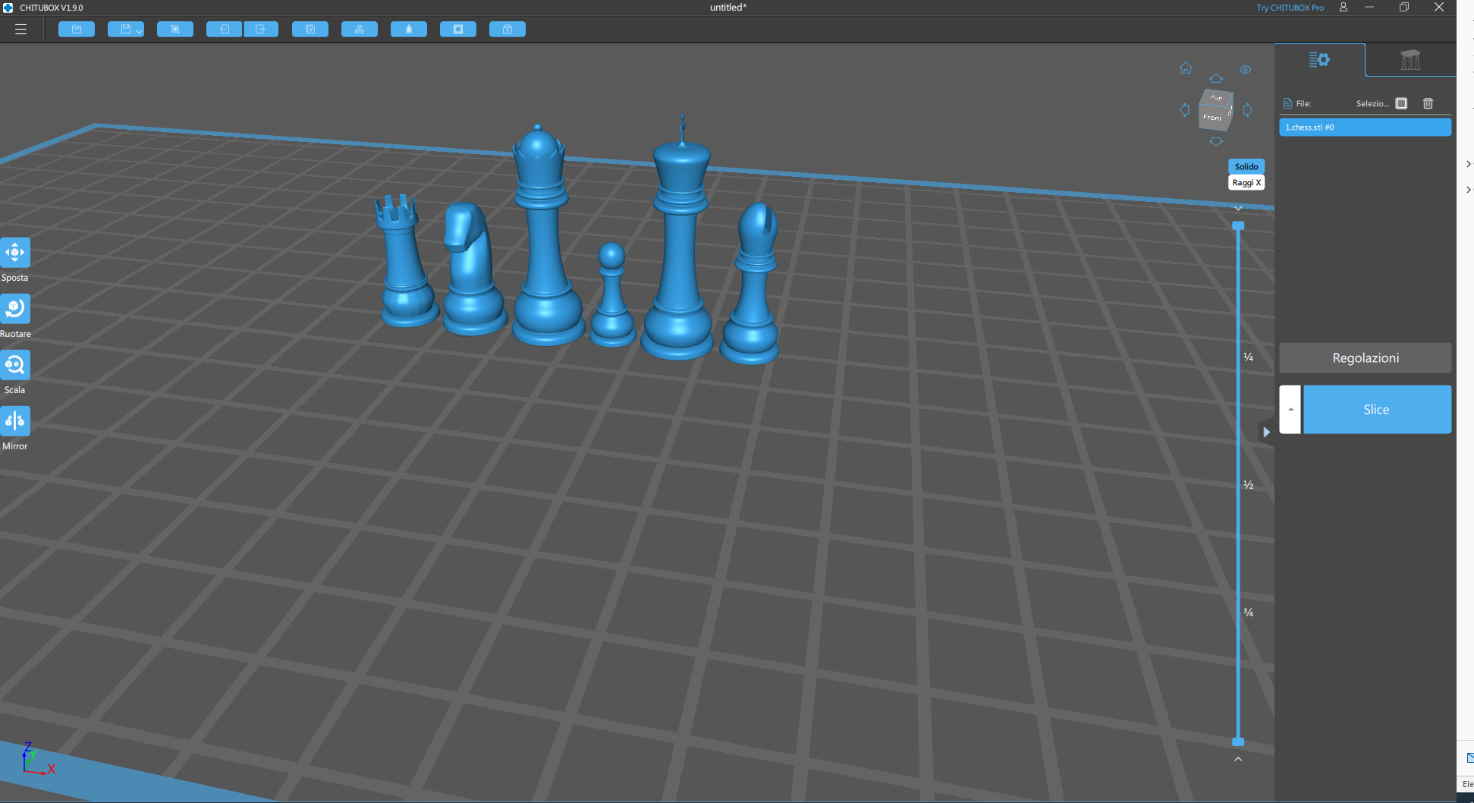


**Figure S41**. Printed objects arrangement and slicing performed using the Chitubox 1.9.4 software for a larger 3D object to demonstrate printing resolution achievable.

|  | **Thickness (mm)** | **Width (mm)** |
| --- | --- | --- |
| **Computer Model** | **2.00** | **5.00** |
| **r1a-3D** | 2.04 ± 0.07 | 5.08 ± 0.09 |
| **r1b-3D** | 2.04 ± 0.05 | 5.07 ± 0.09 |
| **r1c-3D** | 2.06 ± 0.04 | 5.07 ± 0.06 |
| **r2a-3D** | 2.05 ± 0.06 | 5.04 ± 0.06 |
| **r2b-3D** | 2.03 ± 0.04 | 5.03 ± 0.06 |
| **r2c-3D** | 2.06 ± 0.06 | 5.04 ± 0.09 |
| **r3b-3D** | 2.04 ± 0.04 | 5.03 ± 0.06 |
| **r3c-3D** | 2.06 ± 0.06 | 5.03 ± 0.07 |
| Average | 2.05 ± 0.01 | 5.05 ± 0.02 |

**Table S5**. Quantitative evaluation of 3D printing accuracy and resolution, obtained by measuring thickness and with of the narrow section of tensile test specimen compared to the dimensions of the virtual 3D model. Due to the orientation of the 3D models during printing, thickness accuracy is related to the direction perpendicular to the build plate, while the width refers to the accuracy on the layer planes. For 3D printed materials, data are expressed as mean ± SD, obtained by measuring 5 replicates for each sample. The last row reports the mean and SD of the average thickness and width for different resins, revealing no approaciable differences in printing accuracy when the poly(ester-thioether) was changed.


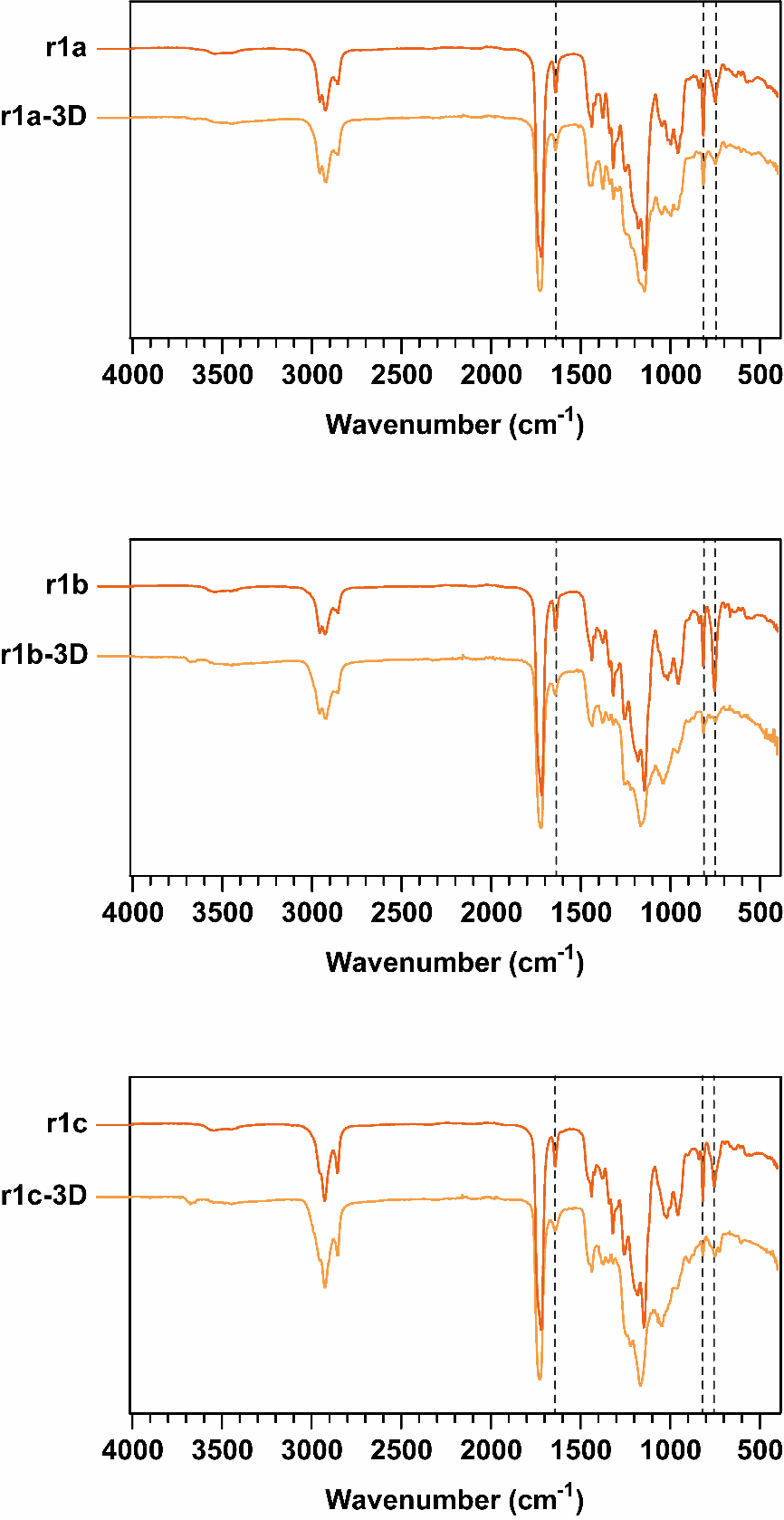


**Figure S42**. ATR-FTIR spectra of formulated photocurable resin before (**r1a**, **r1b** and **r1c**) and after (**r1a-3D**, **r1b-3D** and **r1c-3D**) the 3D printing process. Dashed line correspond to the IR frequencies of 1640 cm^-1^ (C=C stretching), 820 cm^-1^ and 760 cm^-1^ (=C-H bending) in all spectra.


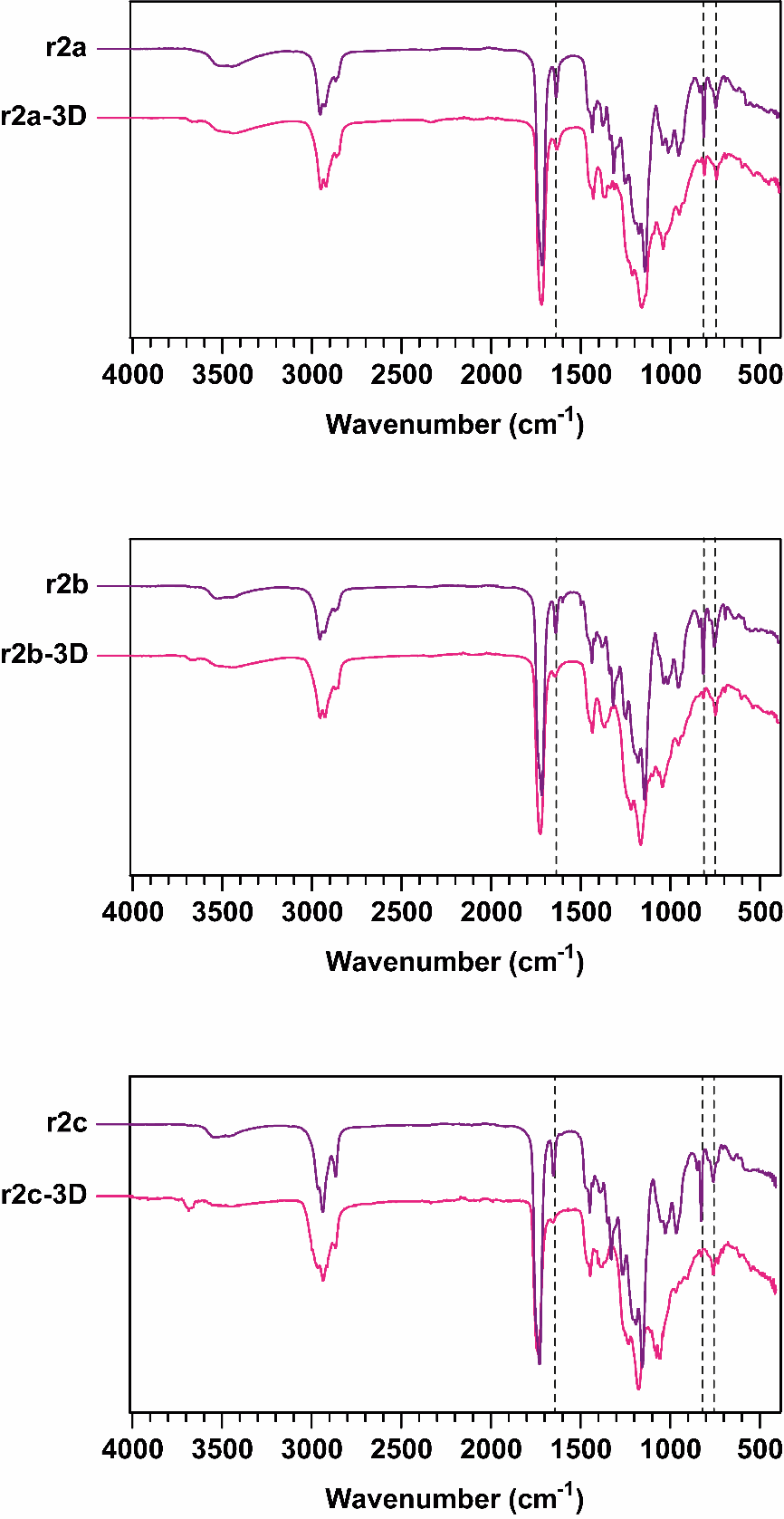


**Figure S43**. ATR-FTIR spectra of formulated photocurable resin before (**r2a**, **r2b** and **r2c**) and after (**r2a-3D**, **r2b-3D** and **r2c-3D**) the 3D printing process. Dashed line correspond to the IR frequencies of 1640 cm^-1^ (C=C stretching), 820 cm^-1^ and 760 cm^-1^ (=C-H bending) in all spectra.

**
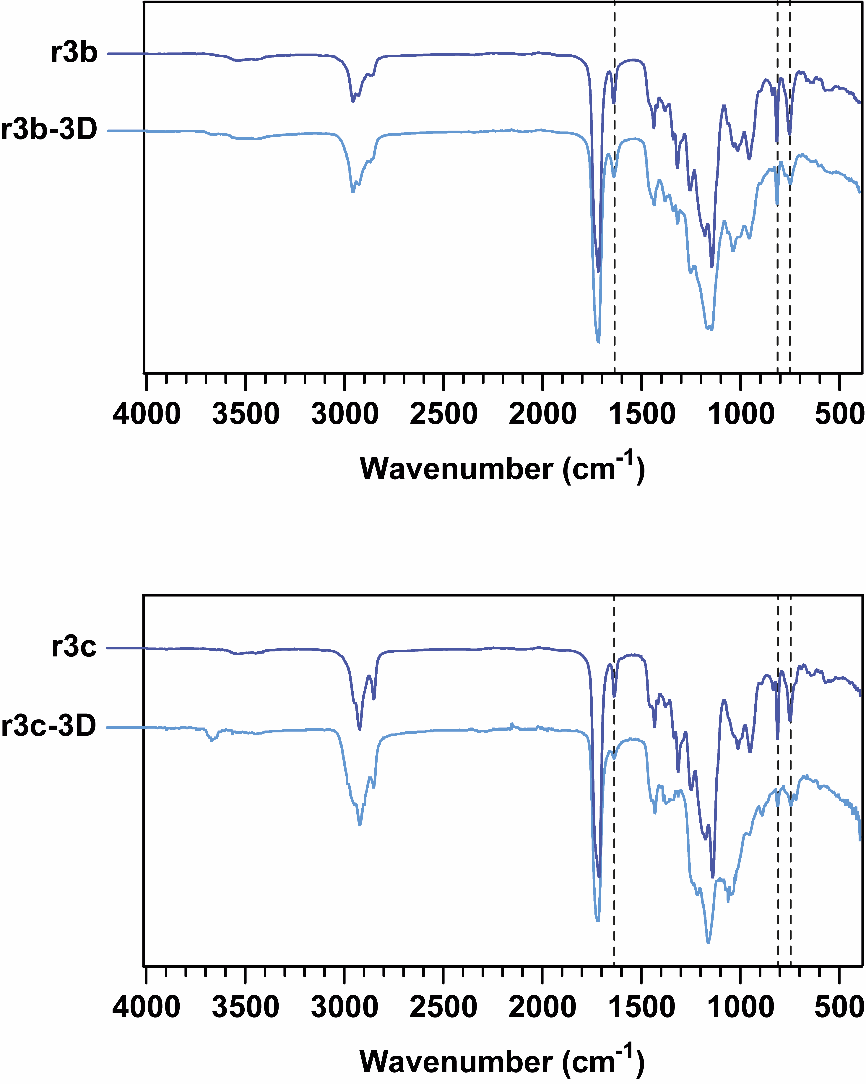
**

**Figure S44**. ATR-FTIR spectra of formulated photocurable resin before (**r3b** and **r3c**) and after (**r3b-3D** and **r3c-3D**) the 3D printing process. Dashed line correspond to the IR frequencies of 1640 cm^-1^ (C=C stretching), 820 cm^-1^ and 760 cm^-1^ (=C-H bending) in all spectra.


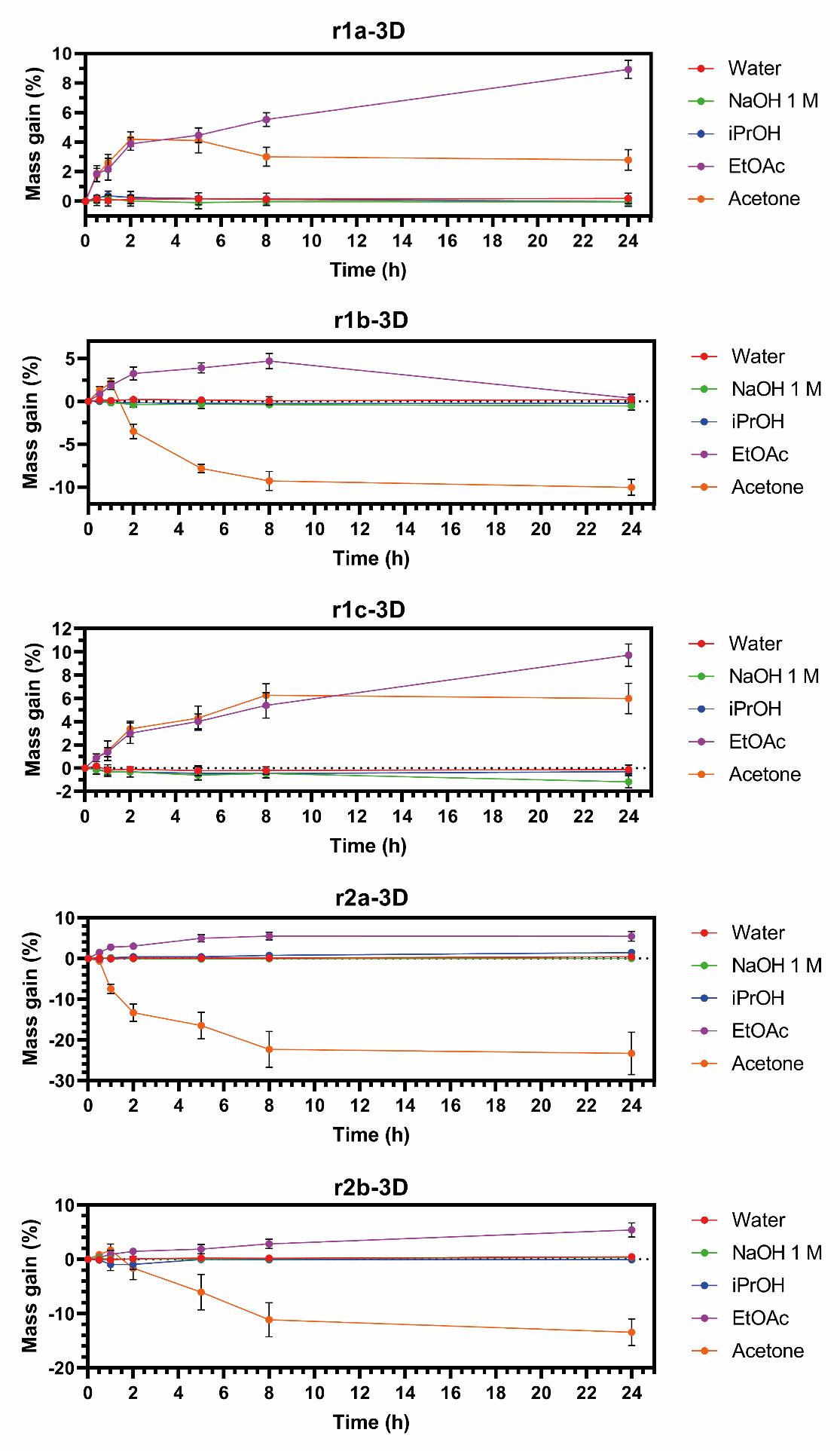


**Figure S45**. Stability of 3D printed materials r1a-3D, r1b-3D, r1c-3D. r2a-3D, and r2b-3D in different solvents over the course of 24 h. Error bars represent the standard deviations obtained with three replicate measurements. Where not present, error bar is smaller than the data point marker.


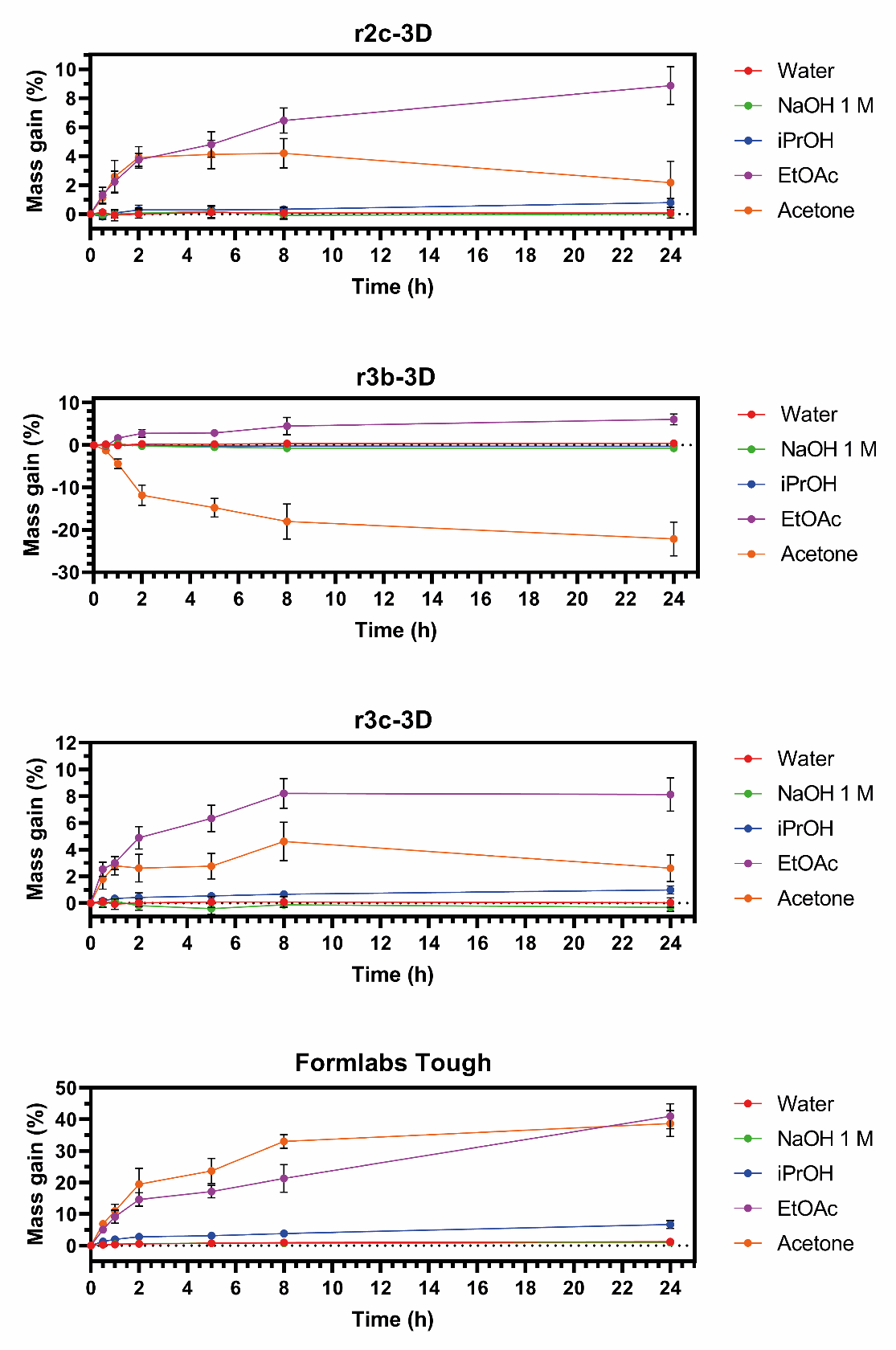


**Figure S46**. Stability of 3D printed materials r2c-3D, r3b-3D, r3c-3D, and Fomlabs Tough in different solvents over the course of 24 h. Error bars represent the standard deviations obtained with three replicate measurements. Where not present, error bar is smaller than the data point marker.


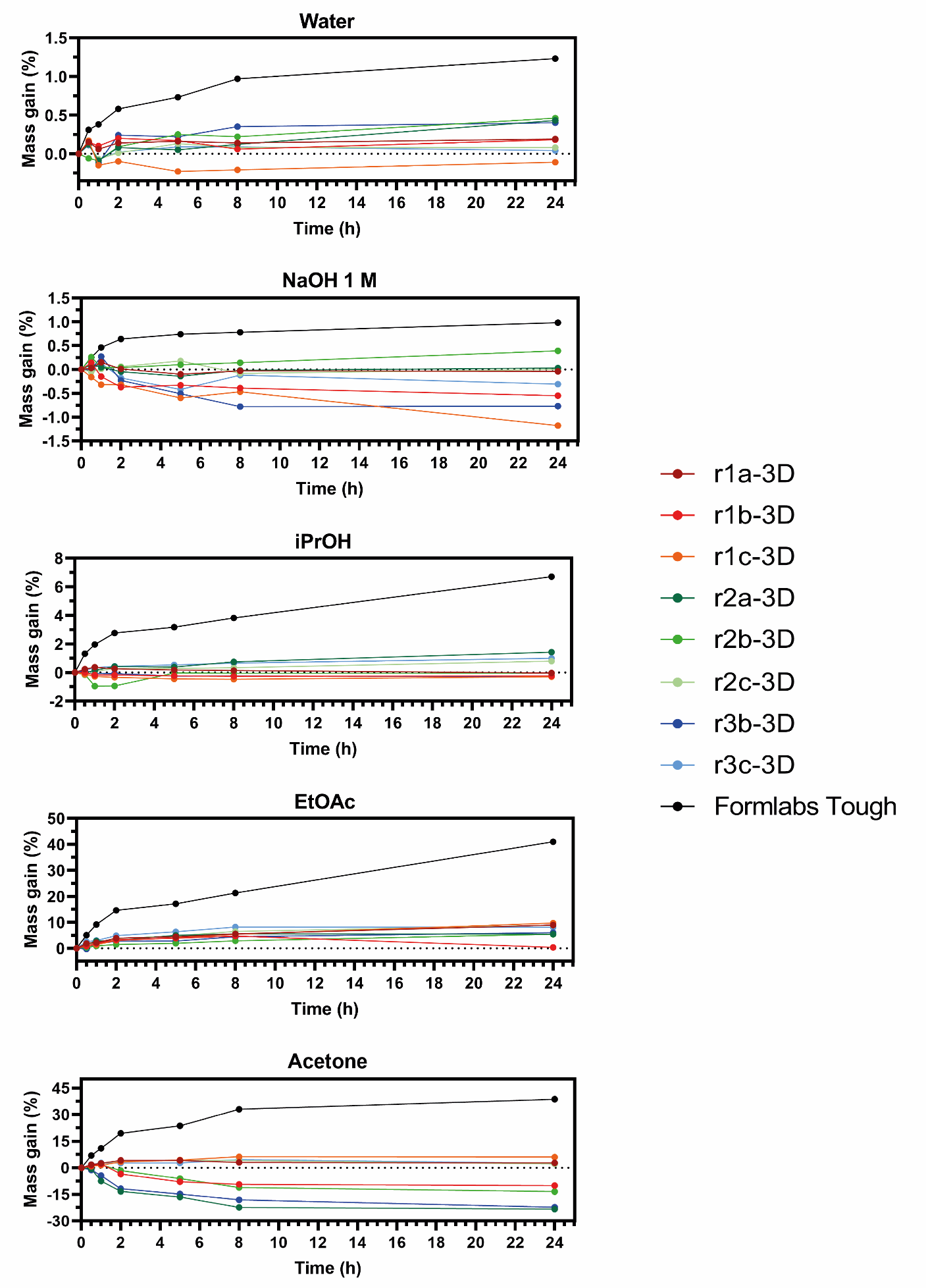


**Figure S47**. Stability of 3D printed materials in different solvents. Plotted data are the same as Figure S45-S46, but grouped by solvent employed for the test. Error bars are not shown for better clarity.

**
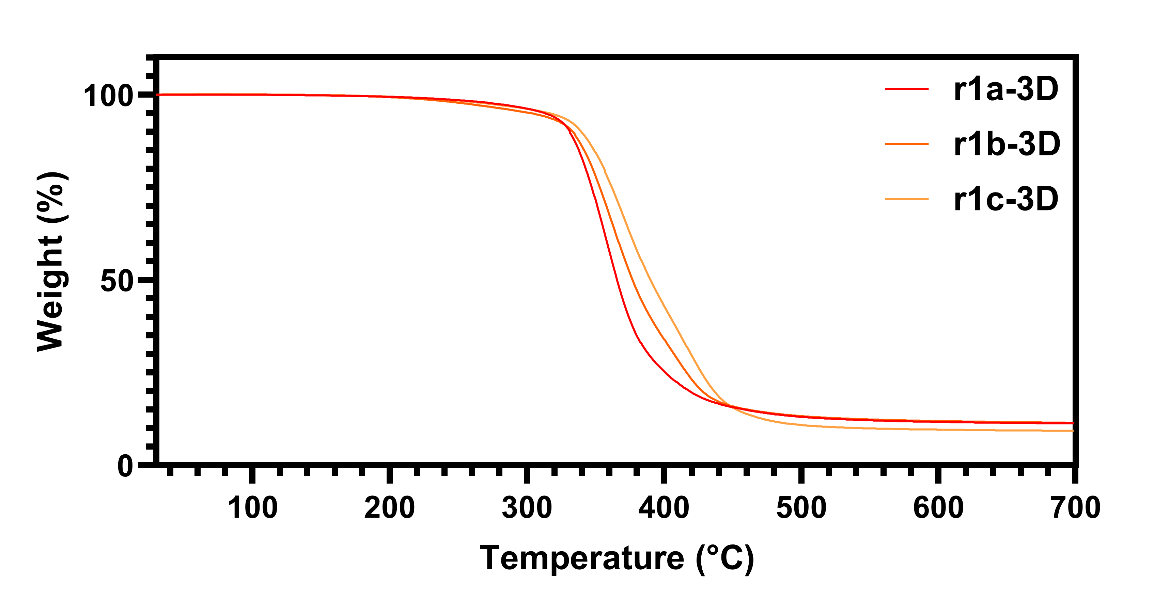

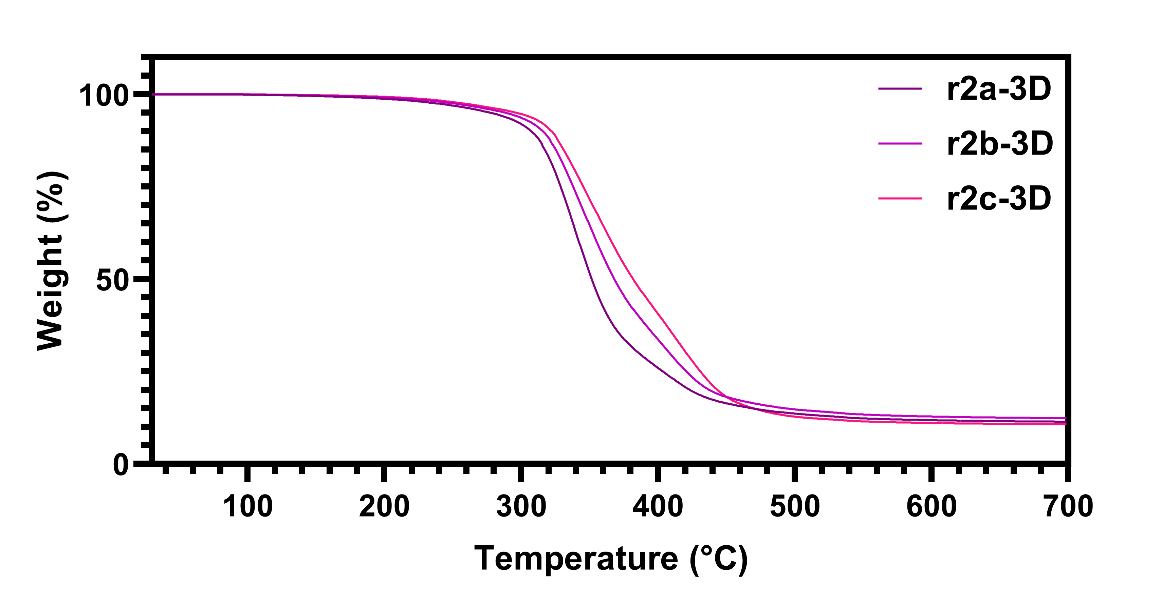

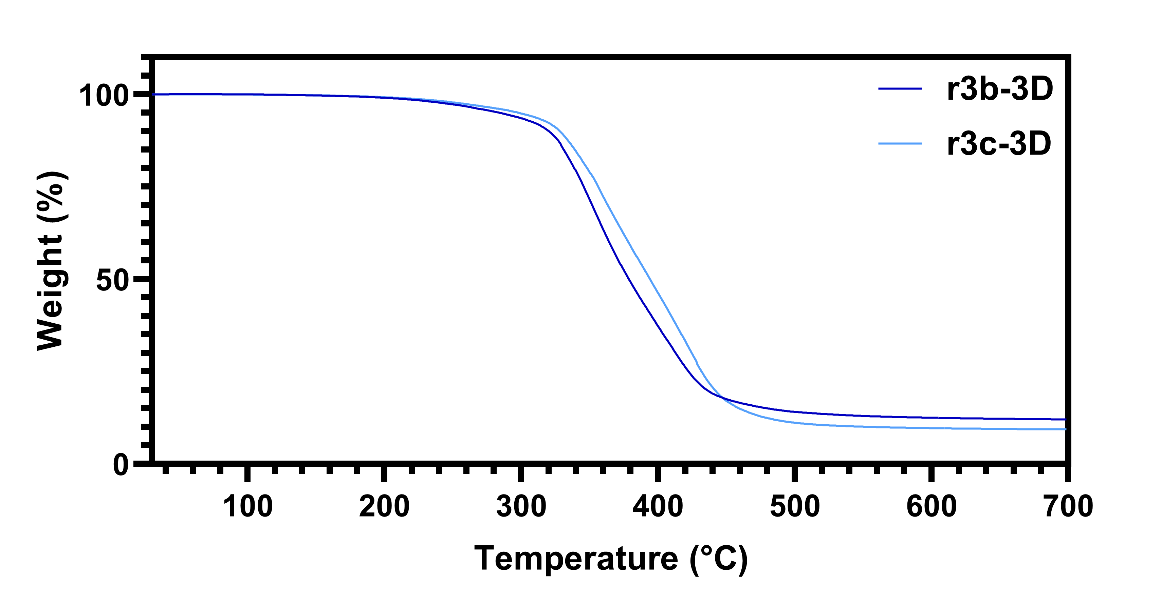
**

**Figure S48**. Thermogravimetric analysis of 3D printed materials

**
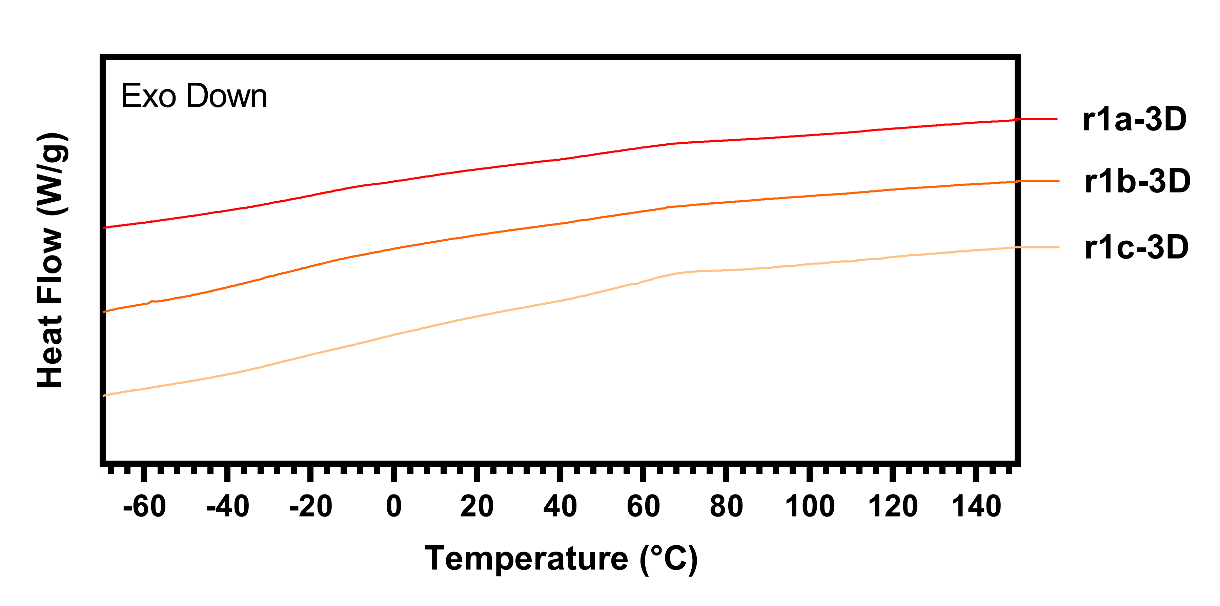

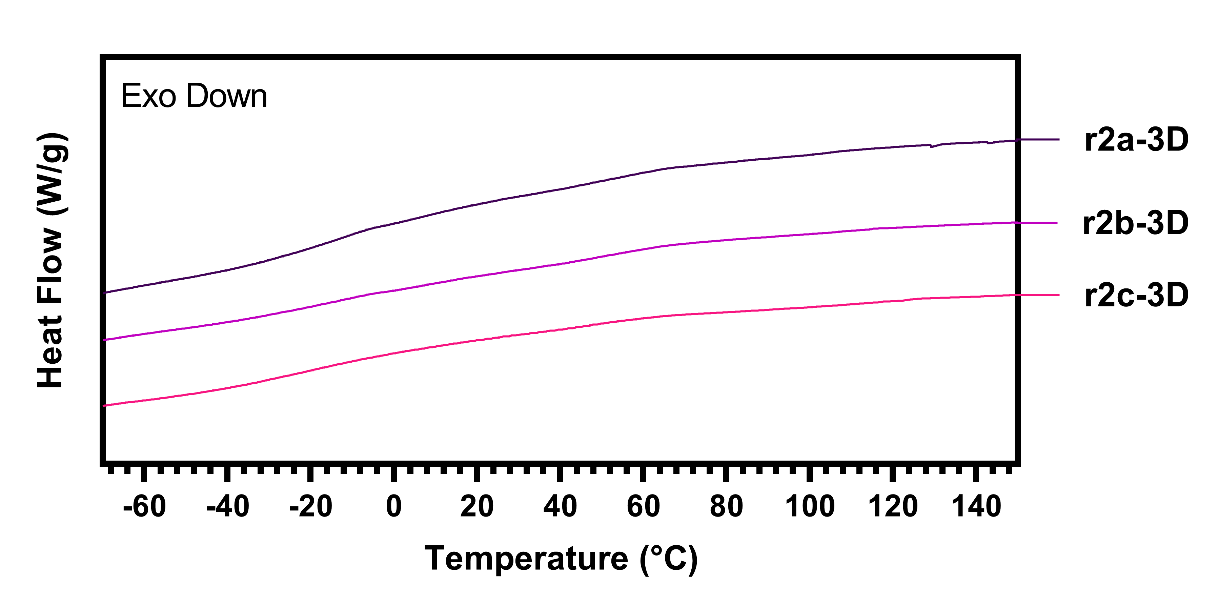

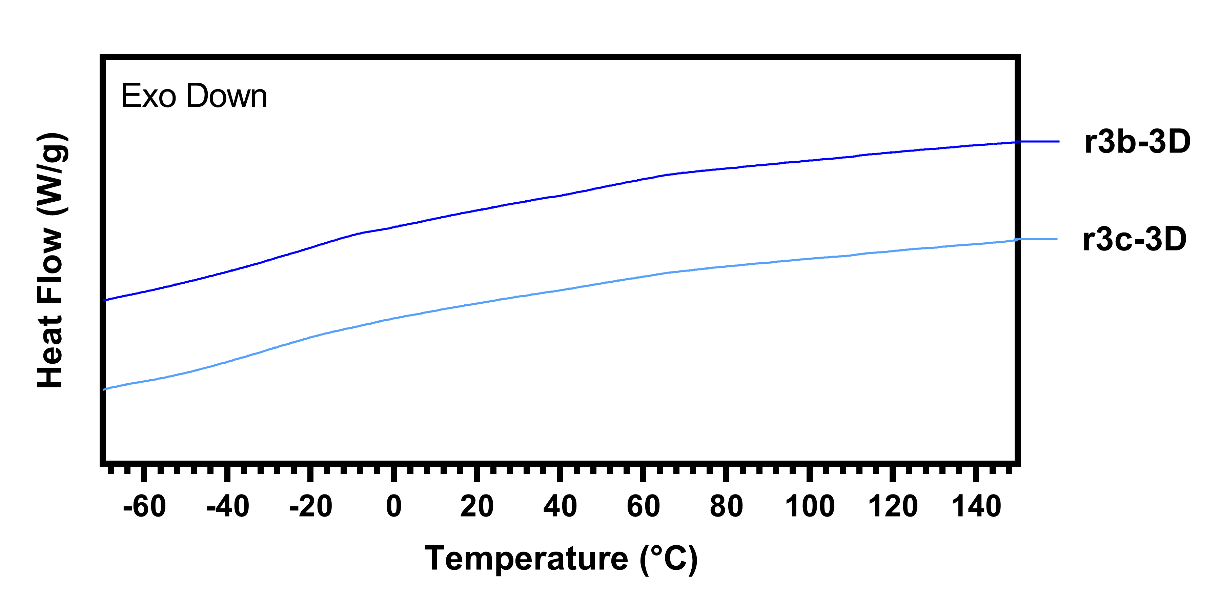
**

**Figure S49**. DSC thermograms of 3D printed materials.

**
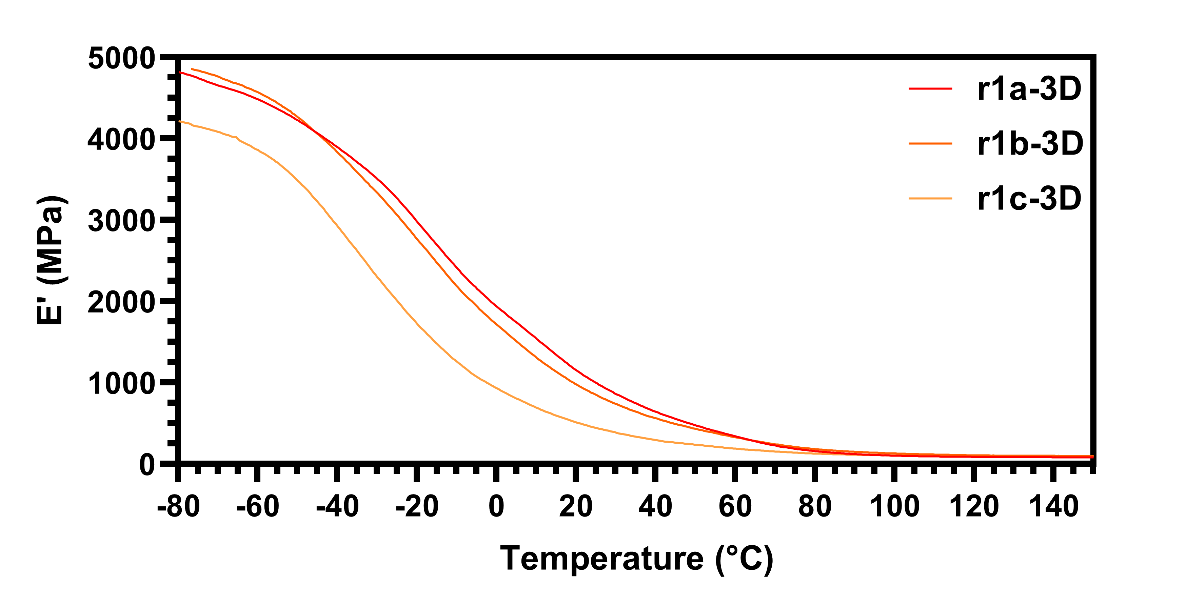

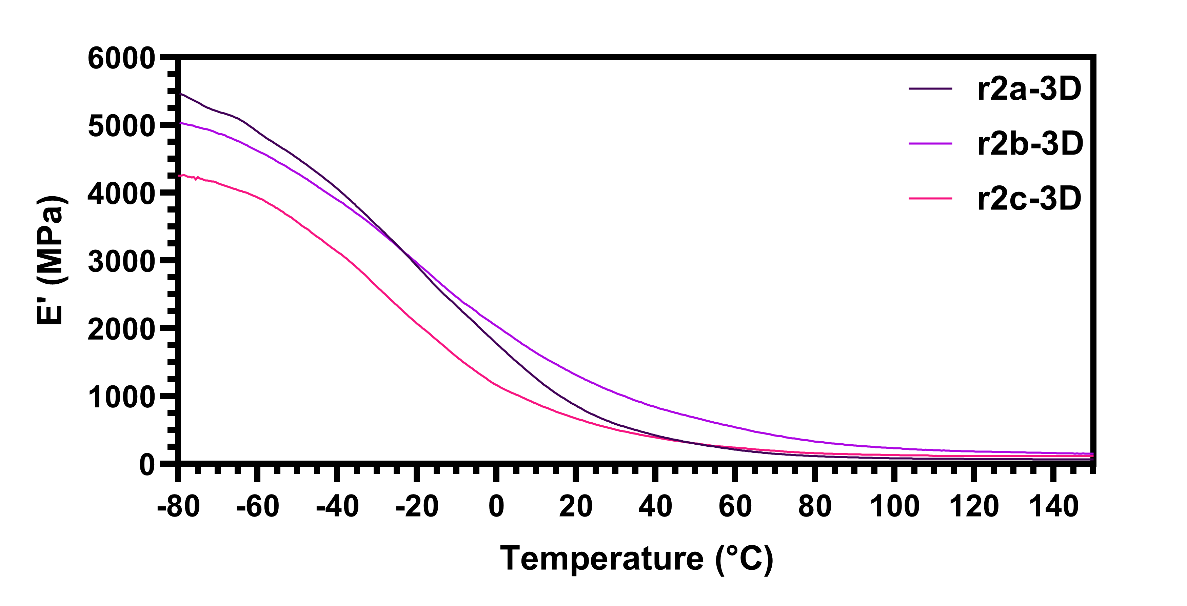

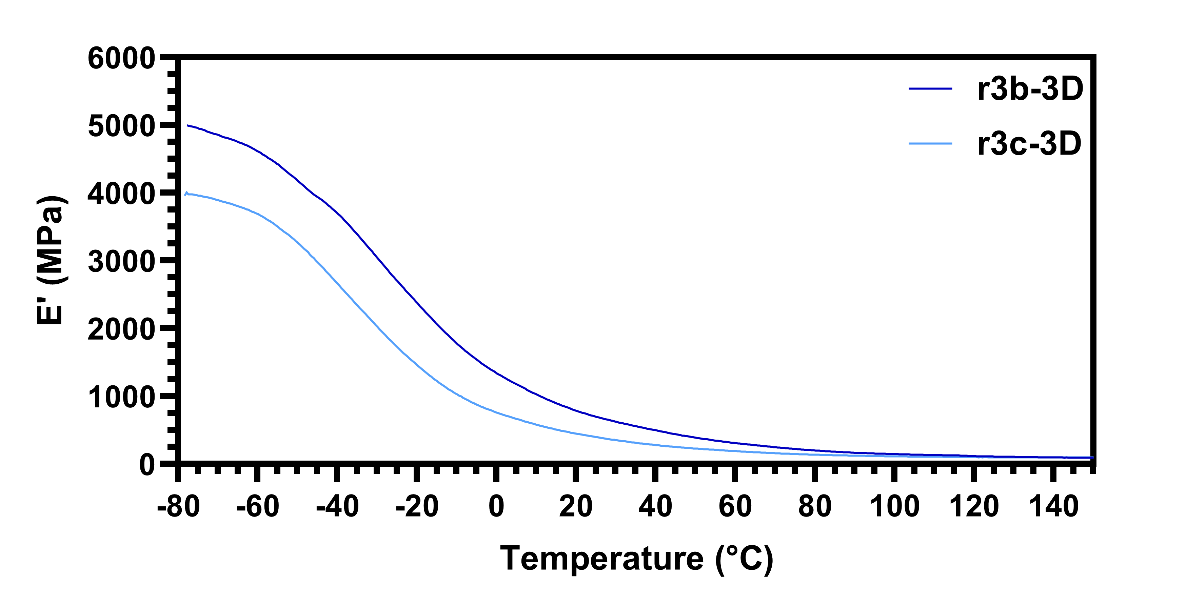
**

**Figure S50.** DMA analysis. Data are grouped by the type of thioether polyol present in the photcurable poly(ester-thioether).


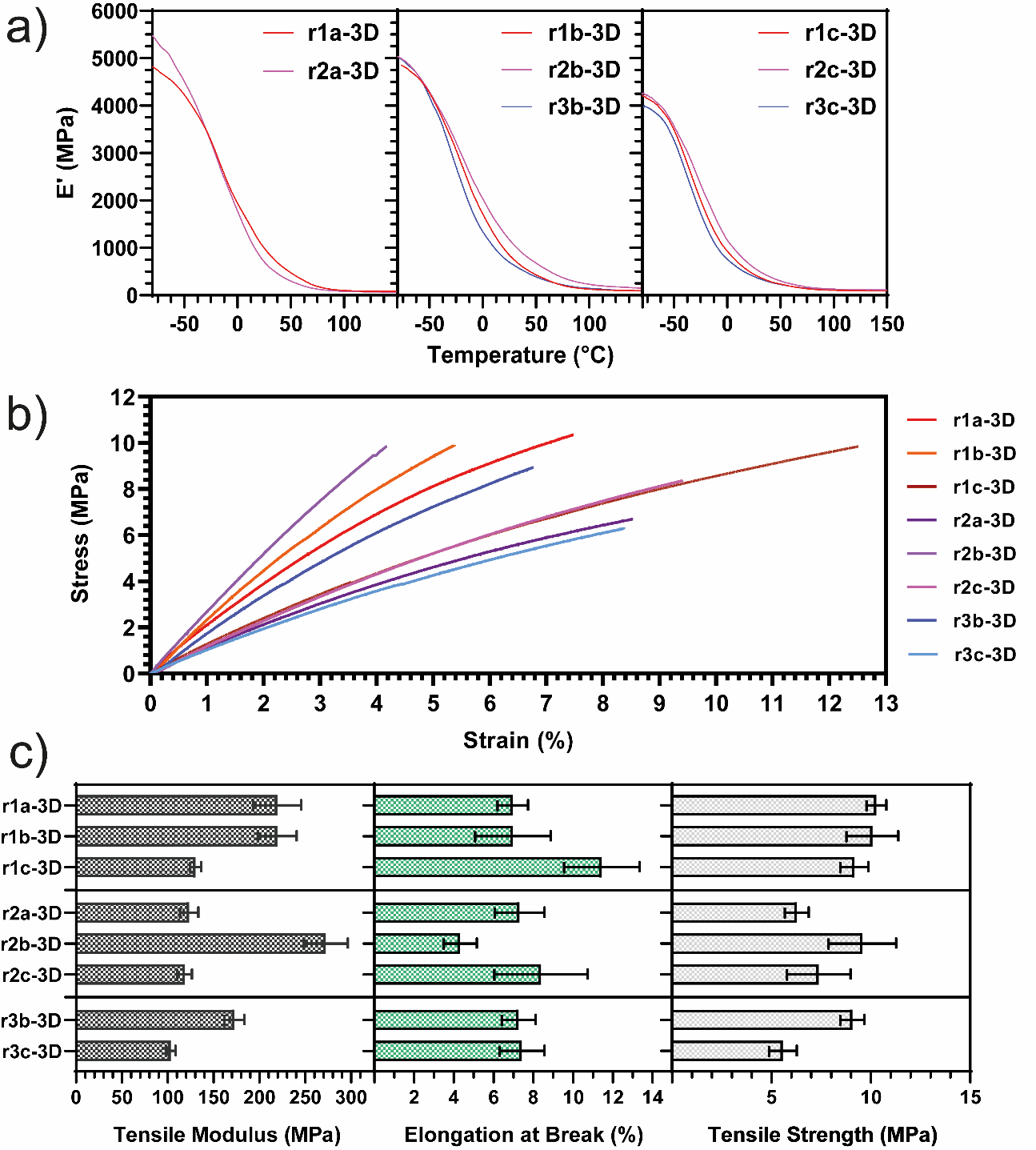


**Figure S51**. Thermomechanical and mechanical characterization of 3D printed materials: a) DMA curves; b) Representative tensile stress-strain curves. The curve closest to the mean behavior of each material was selected amongst the many recorded replicates. c) Comparison of the tensile properties


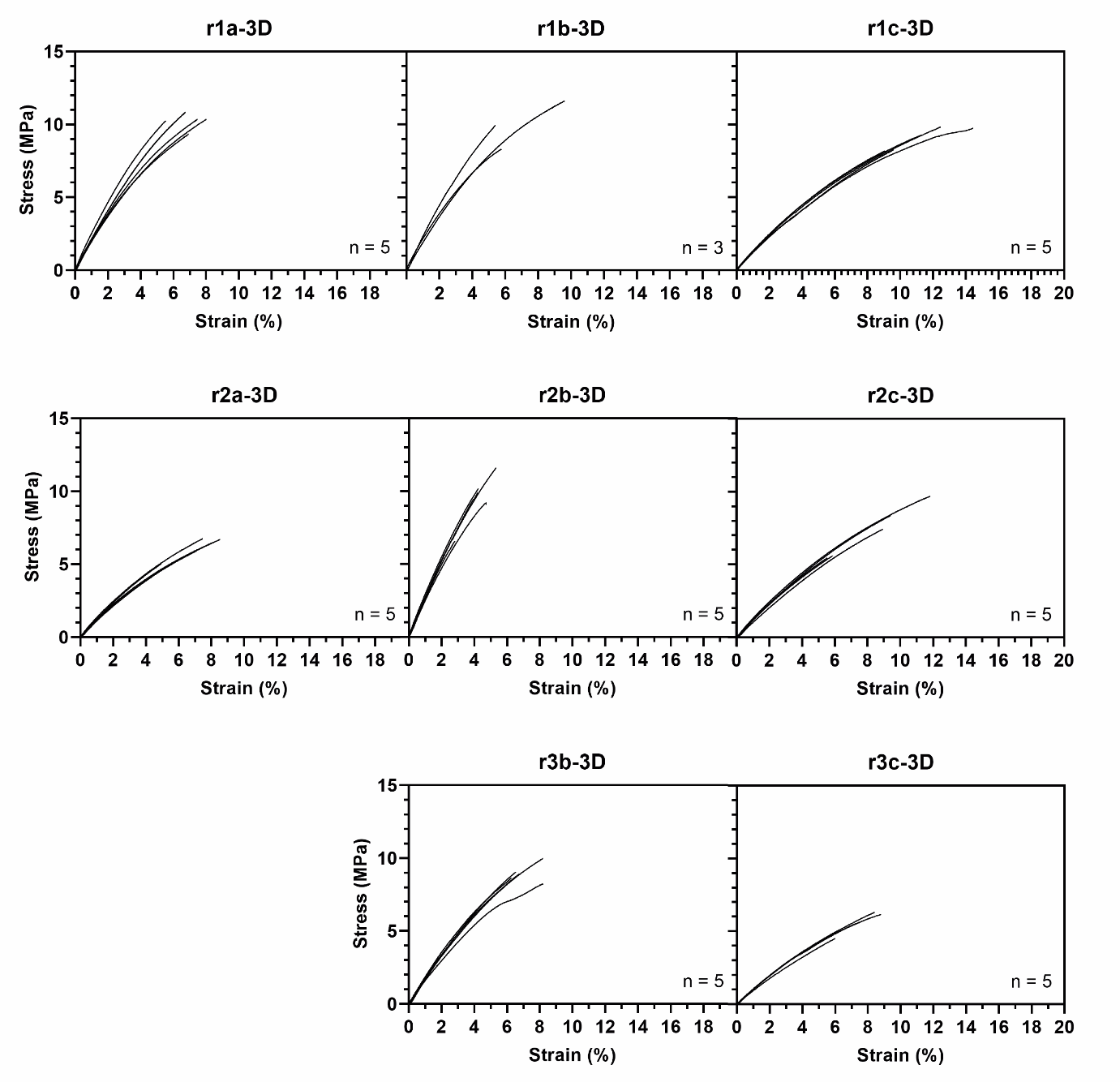


**Figure S52**. Tensile stress-strain curves of 3D printed poly(ester-thioether)-based resins. The number of replicate measurements for each tested sample is reported inside the plot areas.

| Sample | Tensile Modulus^a^  (MPa) | Elongation at Break^a^  (%) | Tensile Strength^a^  (MPa) | Hardness  (Shore D) |
| --- | --- | --- | --- | --- |
| **r1a-3D** | 218 ± 26 | 6.9 ± 0.8 | 10.2 ± 0.5 | 75 ± 1 |
| **r1b-3D** | 218 ± 21 | 6.9 ± 1.9 | 10.0 ± 1.3 | 68 ± 1 |
| **r1c-3D** | 129 ± 6 | 11.4 ± 1.9 | 9.1 ± 0.7 | 70 ± 1 |
| **r2a-3D** | 122 ± 10 | 7.3 ± 1.3 | 6.2 ± 0.6 | 68 ± 1 |
| **r2b-3D** | 271 ± 24 | 4.3 ± 0.8 | 9.5 ± 1.7 | 69 ± 1 |
| **r2c-3D** | 117 ± 8 | 8.3 ± 2.4 | 7.3 ± 1.6 | 66 ± 1 |
| **r3b-3D** | 171 ± 11 | 7.2 ± 0.9 | 9.0 ± 0.6 | 70 ± 1 |
| **r3c-3D** | 102 ± 5 | 7.4 ± 1.1 | 5.5 ± 0.7 | 66 ± 1 |

^a^ Data expressed as mean ± SD

**Table S6**. Tensile and hardness properties of 3D printed materials. The number of replicate measurements is n = 5 for tensile testing and n = 15 for hardness testing.
